# Taxonomic study of the genus *Macquartia* Robineau-Desvoidy (Diptera, Tachinidae) from China

**DOI:** 10.3897/BDJ.11.e106273

**Published:** 2023-10-19

**Authors:** Baihui Zhang, Henan Li, Junjian Li, Dong Zhang, Chuntian Zhang

**Affiliations:** 1 Liaoning Key Laboratory of Evolution and Biodiversity, College of Life Sciences, Shenyang Normal University, Shenyang, China Liaoning Key Laboratory of Evolution and Biodiversity, College of Life Sciences, Shenyang Normal University Shenyang China; 2 School of Ecology and Nature Conservation, Beijing Forestry University, Beijing, China School of Ecology and Nature Conservation, Beijing Forestry University Beijing China

**Keywords:** Calyptratae, Tachininae, East Asia, new species, key

## Abstract

**Background:**

The genus *Macquartia* Robineau-Desvoidy (Diptera, Tachinidae) with 29 known species is a large group of Macquartiini of Tachininae and widely distributed in the Old World and the Nearctic Region.

**New information:**

In this study, Chinese specimens of *Macquartia* were collected and examined, sixteen species are recognised: thirteen previously described, *M.brunneisqua* Zhang et Li, *M.chinensis* Zhang et Li, *M.flavifemorata* Zhang et Li, *M.flavipedicel* Zhang et Li, *M.chalconota* (Meigen), *M.dispar* (Fallén), *M.grisea* (Fallén), *M.macularis* Villeneuve, *M.nudigena* Mesnil, *M.picta* (Meigen), *M.pubiceps* (Zetterstedt), *M.tenebricosa* (Meigen) and *M.viridana* Robineau-Desvoidy and three species new to science, *M.barkamensis* sp. n. (Sichuan), *M.setifacies* sp. n. (Qinghai) and *M.sichuanensis* sp. n. (Sichuan). An identification key to the sixteen species of *Macquartia* known from China is included, along with 80 anatomical figures.

## Introduction

The genus *Macquartia* Robineau-Desvoidy (Diptera, Tachinidae, Tachininae) comprising 29 known species in the tribe Macquartiini is widely distributed in the Old World and the Nearctic Region. *Macquartia* represents one of the most ancient evolutionary lineages of tachinids, parasitising Chrysomelidae larvae (O’Hara et al. 2020, Li et al. 2022). Herting (1960) reported the biology of seven *Macquartia* species in the western Palaearctic Region. Mesnil (1972) reported three genera of Macquartiina (= Macquartiini) in Die fliegen in the Palaearctic Region, two of which (*Bebricia* and *Macquartia*) included 11 species now recognised in the *Macquartia*. Chao et al. (1998) reported six species from China with the key and O’ Hara et al. (2009) catalogued six species of *Macquartia* from China. In Europe, Tschorsnig and Herting (1994) provided a detailed key to the ten Central European species. Cerretti (2006) reported nine species of *Macquartia* in the west Palaearctic Region. Stireman III et al. (2019) reported tribes Macquartiini (*M.tenebricosa*, *M.tessellum*) and Myiophasiini form a clade sister to all other Tachinidae. We follow Mesnil (1972), Tschorsnig and Herting (1994) and Tschorsnig and Richter (1998) in our treatment of *Macquartia* and characterise the genus with generic definition. Li et al. (2022) recently reported four new species of *Macquartia* from Dalaoling Nature Reserve, Hubei, China and phylogenetic implications of Tachinidae. In this study, we examined more than 1130 specimens of *Macquartia*, mainly from China, three new species are described and compared (Table 1) and the diagnoses of thirteen known species are given, based on examined material and related data. Additionally, we provide a key to the sixteen species known from China, along with 80 anatomical figures and the known distributions of 12 species of *Macquartia* in China.

## Materials and methods

The material examined and types during this study are housed in the following collections:

- **BFU** Insect Collection, Beijing Forestry University, Beijing, China;
- **BLKU** Biosystematics Laboratory, Kyushu University, Fukuoka, Japan;
- **IZCAS** National Zoological Museum, Institute of Zoology, Chinese Academy of Sciences, Beijing, China;
- **SYNU** Insect Collection of Shenyang, Normal University, Shenyang, China.

The morphological terminology and measurements used in the descriptions follow Cumming and Wood (2017) and additional terms with restricted application to the taxa are used by the authors (i.e. terms used following Tschorsnig and Richter (1998). The specimens were examined with Zeiss Stemi SV11 stereomicroscopes. Consecutive digital images of heads, abdomens and bodies of male adults were taken with a Leica 205A microscope and images were blended with Leica Application Suite Version 4.12.0. Dissections of male terminalia were carried out following the method described by O’Hara (2002) and dissected terminalia were stored in glycerine in plastic microvials pinned together with the source specimen. The tachinid specimens of this study are deposited in the Insect Collection of Shenyang Normal University, Shenyang (SYNU).

## Taxon treatments

### Macquartia

Robineau-Desvoidy 1830

2D039964-D1A3-57F6-9479-3C2D4FB37E85

>*Macquartia* Robineau-Desvoidy, 1830: 204. Type species: *Macquartiarubripes* Robineau-Desvoidy, 1830 (by designation of Townsend, 1916) [= *Tachinadispar* Fallén, 1820]. References: Mesnil (1972): 1093; Crosskey (1976): 195; Herting (1984): 112; Herting and Dely-Draskovits (1993): 312; Tschorsnig and Herting (1994): 70; Tschorsnig and Richter (1998): 729, 757, 790; Chao et al. (1998): 2043; O’Hara and Wood (2004): 268; Cerretti (2006): 347; O’ Hara et al. (2009): 155; O’Hara and Cerretti (2016): 210; Zhang et al. (2016): 587; O’Hara et al. (2020): 703; Li et al. (2022): 4. Synonyms: see O’Hara et al. (2020): 703-704.
Maquartia . Incorrect subsequent spelling of *Macquartia* Robineau-Desvoidy, 1830 (Rondani (1862: 162)).
Minella Robineau-Desvoidy, 1830: 209. Type species: *Minellanitida* Robineau-Desvoidy, 1830 (= *Tachinatenebricosa* Meigen, 1824), by monotypy [France].
Ptylops Rondani, 1859: 85. Type species: *Macquartiacelebs* Rondani, 1859 (= *Tachinapraefica* Meigen, 1824), by monotypy [Italy].
Gymnopsis Rondani, 1859: 90 (junior homonym of *Gymnopsis* Rafinesque, 1815). Type species: *Tachinachalconota* Meigen, 1824, by monotypy.
Bebricia Robineau-Desvoidy, 1863: 1112. Type species: *Macquartiamicrocera* Robineau- Desvoidy, 1830 (= *Tachinapraefica* Meigen, 1824), by original designation [France].
Javetia Robineau-Desvoidy, 1863: 1115. Type species: *Macquartiagermanica* Robineau- Desvoidy, 1830 (= *Tachinachalconota* Meigen, 1824), by subsequent designation of Townsend (1916: 7) [Germany].
Pherecida Robineau-Desvoidy, 1863: 1118. Type species: *Tachinaegens* Meigen, 1824 (= *Tachinagrisea* Fallén, 1810), by original designation [Europe].
Hesione Robineau-Desvoidy, 1863: 199 (junior homonym of *Hesione* Rafinesque, 1815). Type species: *Hesionemicrocera* Robineau-Desvoidy, 1863 (= *Tachinatessellum* Meigen, 1824), by original designation [France].
Olbya Robineau-Desvoidy, 1863: 170. Type species: *Olbyabrunisquamis* Robineau-Desvoidy, 1863 (= *Tachinatessellum* Meigen, 1824), by monotypy [France].
Arraltia Robineau-Desvoidy, 1863: 72. Type species: *Arraltiaatra* Robineau-Desvoidy, 1863 (= *Tachinapraefica* Meigen, 1824), by monotypy [France].
Rondanimyia Townsend, 1908: 67 (*nomen novum* for *Gymnopsis* Rondani, 1859).
Alaskophyto Townsend, 1915: 285. Type species: *Muscopteryxobscura* Coquillett, 1902, by original designation [United States].
Proteremoplax Enderlein, 1936: 240. Type species: *Tachinachalconota* Meigen, 1824, by subsequent designation of Herting (1984: 113).
Myioclonia Reinhard, 1945: 28. Type species: *Myiocloniaerythrocera* Reinhard, 1945, by original designation [United States].
Hesionella Mesnil, 1972: 1093 (as subgenus of *Macquartia* Robineau-Desvoidy, 1830; junior homonym of *Hesionella* Hartman, 1939). Type species: *Tachinatessellum* Meigen, 1824, by original designation [Europe].
Albiniola Mesnil, 1972: 1094 (as subgenus of *Macquartia* Robineau-Desvoidy, 1830). Type species: Macquartia (Albiniola) nudigena Mesnil, 1972, by original designation [France].
Macquartia Macquartia rubripes Robineau-Desvoidy 1830

#### Description

Body length more than 4.0 mm. Head with eye densely covered with long hairs. Frons of male at most one-fourth eye width in dorsal view, parafacial bare or hairy; proclinate orbital setae absent in male or with two proclinate orbital setae in female. Ocellar setae developed, proclinate. Lower margin of face usually not visible in profile. Facial ridge with setae on lower half or less. Hairs or setulae on posteroventral half of head all black, without pale hairs. Pedicel and basal half of postpedicel dark brown or reddish-yellow, postpedicel less than two times as long as pedicel. Arista thickened on basal one-fourth or less, first aristomere distinctly shorter, arista bare or short pubescent, the longest hairs longer than basal diameter of arista or plumose. Palpus dark brown or at least reddish-yellow at apex. Thorax with scutum before suture without dark stripes, 2-3 presutural and 3-4 postsutural dorsocentral setae and 2 or 3 postsutural intra-alar setae. Scutellum usually black, at most reddish-yellow at apical half, with three or more pairs of marginal setae, with strongly crossed apical scutellar setae, but weaker than subapical scutellar setae, lateral scutellar setae absent or present. Prosternum and proepisternum bare. Postpronotal lobe with 2 or 3 setae, arranged in a straight line. Katepisternum with 2 or 3 setae, anepimeral seta well-developed, anatergite almost always with a group of minute hairs or setulae below lower calypter. Basicosta dark brown or reddish-yellow. Second costal section of wing with fine hairs ventrally. Only base of vein R_4+5_ with hairs. Wing cell r_4+5_ open or with a very short petiole, bend of vein M without continuation or with a very short stub. Dorsal surface of lower calypter bare, divergent from scutellum or not divergent from scutellum. Legs usually black, sometimes reddish-yellow. Inner anterior surface of fore coxa bare or predominantly bare. Mid-tibia with 1 or 2-5 anterodorsal setae, hind tibia with 2 or 3 pre-apical dorsal setae. Pre-apical posteroventral seta on hind tibia distinctly shorter than pre-apical anteroventral seta. Abdomen black, syntergite 1+2 medially excavated to its hind margin or not, without or with 2 median marginal setae, 3^rd^ tergite with 2 or a row of median marginal and 2 (weak) median discal setae in male and absent in female and 1-3 pairs of lateral discal setae, 4^th^ tergite with a row of marginal, 2 to a row of discal setae in male and 2 setae in female or 3^rd^ and 4^th^ tergites without median discal setae in both sexes. 5^th^ tergite with a row of or two rows of median discal and a row of marginal setae. Male terminalia very long, middle part of cerci narrowed and pointed, surstyli more or less narrowed.

#### Diagnosis

Eye densely covered with long hairs, ocellar setae developed, proclinate, hairs or setulae on posteroventral half of head all black, without pale hairs. Antenna with postpedicel at most two times as long as pedicel, arista thickened on basal one-fourth or less. Body black, prosternum bare, scutellum with strong crossed apical setae, anepimeral seta well-developed, anatergite almost always with a group of minute hairs or setulae below lower calypter. Second costal section of wing with fine hairs ventrally. Pre-apical posteroventral seta on hind tibia distinctly shorter than pre-apical anteroventral seta.

### Macquartia barkamensis

Zhang & Zhang sp. nov.

513EABDE-49C3-5479-96B4-DF0CF6CC6513

9BF85DCE-00D4-4966-852A-3A2D9E27A63B

#### Materials

- **Type status:** Holotype. **Occurrence:** recordedBy: Zhang Chun-Tian; individualCount: 1; sex: male; lifeStage: adult; occurrenceID: 726CAD2F-64CF-540D-9878-C1CF8FE2D1C7; **Taxon:** scientificName: Macquartiabarkamensis; **Location:** country: China; stateProvince: Sichuan; locality: Songgang County, Maerkang; verbatimElevation: 2480 m; verbatimCoordinates: 31.9°N, 102.06°E; decimalLatitude: 31.9; decimalLongitude: 102.06; **Identification:** identifiedBy: Zhang Chun-tian; dateIdentified: 2022; **Event:** samplingProtocol: sweeping; eventDate: 13/08/2017; **Record Level:** collectionCode: Insects

#### Description

Body length 6.5 mm (Fig. 1a, b).

**Male**. Head black（Fig. 1c, d, with greyish-white pruinosity on fronto-orbital plate, parafacial and face; lower face, lower occiput and genal dilation with dark greyish pruinosity; frontal vitta dark brown; lunule dark brown; upper occiput black. Antenna dark brown, except for base of 1^st^ postpedicel brown; palpus reddish-yellow, apex and base brown. Frons strongly narrowed above, at the narrowest point slightly wider than anterior ocellus width, in profile about 1.5 times as long as face; frontal vitta almost linear in front of ocellar triangle and strongly widened anteriorly; parafacial nearly parallel-sided, 1.5-2 times as wide as (nearly as wide as in profile) 1^st^ postpedicel; face weakly concave, very weakly carinate on upper median portion between base of antenna, lower portion very weakly, but warped forward; gena about 3/10 (about 1/4 in profile) as high as eye height; occiput flattened, not bulged. Inner vertical setae fine and long, hair-like, not different from postocular setae row; ocellar seta slender, hair-like, about as long as upper frontal setae; 9-10 frontal setae, 2 uppermost setae finer and shorter, lowest seta arising nearly with middle level of pedicel; inner fronto-orbital plate with dense hairs, parafacial almost bare, only with 5-6 hairs below lowest fronto-orbital seta; facial ridge with 1 long and 1-2 short and fine setae on lower 1/5; 7-8 strong subvibrissal setae; postocular setae close to posterior eye margin, long and directed forward on basal 1/3-1/4. Base of antenna nearly level with middle of eye; antenna short, at most 2/3 as long as face; pedicel with a long seta which is nearly as long as 1^st^ postpedicel; 1^st^ postpedicel about 2.5 times as long as wide and about 2 times as long as pedicel; arista short pubescent, about 1.3 times as long as pedicel and 1st postpedicel together, thickened on basal 1/5-1/6, 2^nd^ aristomere as long as wide. Prementum about 2.7 times as long as wide, about as long as genal height; palpus about slightly longer than antenna.

Thorax black in ground colour, with very thin greyish-white pruinosity on postpronotal lobe, presutural area of scutum and proepimeron. Hairs black, rather sparse short and erect or suberect on scutum and scutellum and slender on pleura; 1 presutural and 1 postsutural acrostichal setae; 2 presutural and 3 postsutural dorsocentral setae; 2 postsutural intra-alar seta; pre-alar seta about as long as notopleural seta and 2^nd^ supra-alar seta; 3 pairs of reclinate and strong marginal scutellar setae; apical scutellar setae crossed, about twice as long as scutellum; with some erect slender seta-like hairs on scutellum. Two katepisternal setae and anterior one weak; 1 anepimeron seta; 1 upper anterior and a row of (5) posterior anepisternal setae; 1 anepimeronal seta, thin and long; anatergite hairy. Wing hyaline, weakly tinged with pale brown, more strongly tinged on basal portion; tegula dark brown; basicosta reddish-yellow; lower calypter yellowish, fringe yellow; halters mostly yellow, except for base brown. Lower calypter divergent from scutellum. Costal spine short, at most 1/2 length of crossvein r-m, base of vein R with 2-3 fine setulae dorsally and ventrally, relative lengths of costal sectors 2^nd^, 3^rd^ and 4^th^ approximately as 1:3:1.2; vein M from dm-cu crossvein to its bend about 4 times distance between the bend and wing hind margin. Legs black or dark brown, pulvilli yellowish, claws and pulvilli nearly as long as or longer than 5^th^ tarsomere. Fore tibia with a row of 6 short anterodorsal setae, lowest one strong, about twice as long as others, 2 posterodorsal setae, pre-apical anterodorsal seta about as long as pre-apical dorsal seta; mid-tibia with 1 anterodorsal seta, 2 posterodorsal setae, 1 ventral seta; hind tibia with 3-4 anterodorsal, 3 posterodorsal and 1-2 ventral setae, if 2, upper one short; 1 pre-apical anterodorsal seta slightly shorter than 1 pre-apical dorsal seta; pre-apical anteroventral seta distinctly longer than pre-apical posteroventral seta.

Abdomen oblong shiny black, covered with sparse grey pruinosity. Mid-dorsal excavation of syntergite 1+2 extending to base, with 2 median marginal and 2 pairs of lateral marginal setae and with some lateral discal setae; 3^rd^ tergite with 4 median marginal and a pair of lateral marginal, 2 median discal and a pair of lateral discal setae; each 4^th^ and 5^th^ tergite separately with a row of marginal and discal setae. Male terminalia as in Fig. 2. Sternite 5 triangular, distinctly prominent at base,

Sternite 5 with deep V-shaped median cleft, about 2/5 of the sternite length, posterior lobe bluntly rounded apically and inner margin slightly pointed at apex. Pregonite short and bluntly rounded, postgonite hook-like pointed at apex, distiphallus fork-like with some minute spinules on surface. In caudal view, cerci slender and apex pointed, surstylus slightly blunt at apex. In lateral view, cerci and surstylus slender and slightly bent backwards.

**Female**. Unknown.

#### Diagnosis

Parafacial hairy only on upper half. Palpi reddish-yellow to brown. Two katepisternal setae. Lower calyptrae divergent from scutellum. Mid-tibia with 1 anterodorsal seta. Abdominal syntergite 1+2 not medially excavate to its posterior margin, separately with two pairs of lateral marginal and lateral discal setae, 3^rd^ tergite with 4 median marginal and a pair of lateral marginal setae and 2 median discal and a pair of lateral discal setae, 4^th^ tergite with a row of discal setae.

#### Etymology

The specific epithet is taken from a locality of this species, i.e. Barkam = Maerkang City, Aba Prefecture, Sichuan, China.

#### Distribution

Oriental China (collected once from Maerkang City, Aba Prefecture, Sichuan).

#### Remarks

This species is similar to *M.grisea* (Fallén), but it is distinguished from the latter in having 2 katepisternal setae, 3^rd^ tergite with 4 median marginal and 2 median discal setae and a pair of lateral discal setae, 4^th^ tergite separately with a row of marginal and discal setae.

### Macquartia setifacies

Zhang & Li sp. nov.

3F169912-2066-5207-A721-707A4663A504

F040568D-FC7E-453A-AD2F-DE96EE7E7BBC

#### Materials

- **Type status:** Holotype. **Occurrence:** recordedBy: Hao Bo; individualCount: 1; sex: male; lifeStage: adult; occurrenceID: 7D45121D-ABEC-58C4-9B2F-FA6FFCDF7234; **Taxon:** scientificName: Macquartiasetifacies; **Location:** country: China; stateProvince: Qinghai; locality: Mt. Kongdaban, Qilian Mountains; verbatimElevation: 3384 m; verbatimCoordinates: 38. 90°N, 100.38°E; decimalLatitude: 38.9; decimalLongitude: 100.38; **Identification:** identifiedBy: Zhang Chun-tian; dateIdentified: 2022; **Event:** samplingProtocol: sweeping; eventDate: 19/07/2019; **Record Level:** collectionCode: Insects
- **Type status:** Paratype. **Occurrence:** recordedBy: Hao Bo; individualCount: 1; sex: male; lifeStage: adult; occurrenceID: F241DD22-5919-55F7-A6B2-D16B8335C2EF; **Taxon:** scientificName: Macquartiasetifacies; **Location:** country: China; stateProvince: Qinghai; locality: Mt. Kongdaban, Qilian Mountains; verbatimElevation: 3384 m; verbatimCoordinates: 38. 90°N, 100.38°E; decimalLatitude: 38.9; decimalLongitude: 100.38; **Identification:** identifiedBy: Zhang Chun-tian; dateIdentified: 2022; **Event:** samplingProtocol: sweeping; eventDate: 19/07/2019; **Record Level:** collectionCode: Insects
- **Type status:** Paratype. **Occurrence:** recordedBy: Hao Bo; individualCount: 1; sex: male; lifeStage: adult; occurrenceID: 1818F814-31B3-5A94-A4EB-D0348AA18FA4; **Taxon:** scientificName: Macquartiasetifacies; **Location:** country: China; stateProvince: Qinghai; locality: Guxiangsigou, Menyuan County; verbatimElevation: 2408 m; verbatimCoordinates: 37. 60°N, 102.20°E; decimalLatitude: 37.6; decimalLongitude: 102.2; **Identification:** identifiedBy: Zhang Chun-tian; dateIdentified: 2022; **Event:** samplingProtocol: sweeping; eventDate: 14-15/07/2019; **Record Level:** collectionCode: Insects
- **Type status:** Paratype. **Occurrence:** recordedBy: Hao Bo; individualCount: 2; sex: female; lifeStage: adult; occurrenceID: 3765F655-FA53-520B-8B39-89CA8DA4D5BD; **Taxon:** scientificName: Macquartiasetifacies; **Location:** country: China; stateProvince: Qinghai; locality: Guxiangsigou, Menyuan County; verbatimElevation: 2408 m; verbatimCoordinates: 37. 60°N, 102.20°E; decimalLatitude: 37.6; decimalLongitude: 102.2; **Identification:** identifiedBy: Zhang Chun-tian; dateIdentified: 2022; **Event:** samplingProtocol: sweeping; eventDate: 14-15/07/2019; **Record Level:** collectionCode: Insects
- **Type status:** Paratype. **Occurrence:** recordedBy: Hao Bo; individualCount: 1; sex: female; lifeStage: adult; occurrenceID: F146813F-D7C5-500E-AFDA-D9718FCBDC0B; **Taxon:** scientificName: Macquartiasetifacies; **Location:** country: China; stateProvince: Qinghai; locality: Qihankai, Xianmi, Menyuan County; verbatimElevation: 2671 m; verbatimCoordinates: 37.90°N, 102.10°E; decimalLatitude: 37.9; decimalLongitude: 102.1; **Identification:** identifiedBy: Zhang Chun-tian; dateIdentified: 2022; **Event:** samplingProtocol: sweeping; eventDate: 16/07/2019; **Record Level:** collectionCode: Insects
- **Type status:** Paratype. **Occurrence:** recordedBy: Hao Bo; individualCount: 2; sex: male; lifeStage: adult; occurrenceID: 6E568ABE-63CB-50C5-9848-E4CF14FAAFBE; **Taxon:** scientificName: Macquartiasetifacies; **Location:** country: China; stateProvince: Qinghai; locality: Deqin, Xianmi, Menyuan County; verbatimElevation: 3384 m; verbatimCoordinates: 37.18°N, 102.80°E; decimalLatitude: 37.18; decimalLongitude: 102.8; **Identification:** identifiedBy: Zhang Chun-tian; dateIdentified: 2022; **Event:** samplingProtocol: sweeping; eventDate: 18/07/2019; **Record Level:** collectionCode: Insects
- **Type status:** Paratype. **Occurrence:** recordedBy: Hao Bo; individualCount: 1; sex: male; lifeStage: adult; occurrenceID: AE271F1D-DE0E-5C50-96FD-FD8F8C7F5DC4; **Taxon:** scientificName: Macquartiasetifacies; **Location:** country: China; stateProvince: Qinghai; locality: Youhlu, Qilian Mountains; verbatimElevation: 2990- 3140 m; verbatimCoordinates: 38.15°N, 99.47°E; decimalLatitude: 38.15; decimalLongitude: 99.47; **Identification:** identifiedBy: Zhang Chun-tian; dateIdentified: 2022; **Event:** samplingProtocol: sweeping; eventDate: 24/07/2019; **Record Level:** collectionCode: Insects
- **Type status:** Paratype. **Occurrence:** recordedBy: Li Jun-Jian; individualCount: 2; sex: male; lifeStage: adult; occurrenceID: 4BF6A1E0-98ED-5C67-9BEF-6F4CE642D879; **Taxon:** scientificName: Macquartiasetifacies; **Location:** country: China; stateProvince: Qinghai; locality: Mt. Kongdaban, Qilian Mountains; verbatimElevation: 3196 m; verbatimCoordinates: 38. 20°N, 100.38°E; decimalLatitude: 38.2; decimalLongitude: 100.38; **Identification:** identifiedBy: Zhang Chun-tian; dateIdentified: 2022; **Event:** samplingProtocol: sweeping; eventDate: 15/08/2019; **Record Level:** collectionCode: Insects
- **Type status:** Paratype. **Occurrence:** recordedBy: Li Jun-Jian; individualCount: 1; sex: female; lifeStage: adult; occurrenceID: EAE46C1E-BE8A-51F9-8B5C-17903F257D90; **Taxon:** scientificName: Macquartiasetifacies; **Location:** country: China; stateProvince: Qinghai; locality: Shenxiangou, Maixiu, Zeku County; verbatimElevation: 3196 m; verbatimCoordinates: 35.16°N, 101.55°E; decimalLatitude: 35.16; decimalLongitude: 101.55; **Identification:** identifiedBy: Zhang Chun-tian; dateIdentified: 2022; **Event:** samplingProtocol: sweeping; eventDate: 26/08/2019; **Record Level:** collectionCode: Insects

#### Description

Body length 6.0-8.1 mm (Fig. 3a, b).

**Male**. Head black（Fig. 3c, d）, with dense greyish-white pruinosity on fronto-orbital plate, parafacial and face; lower face, lower occiput and genal dilation with dark greyish pruinosity; frontal vitta dark brown; ocelli reddish; lunule dark brown; upper occiput black. Eye with dense yellow long hairs. Antenna dark brown, except for basal of 1^st^ postpedicel brown; palpus black. Frons strongly narrowed above, at the narrowest point as wide as 1^st^ postpedicel or twice as wide as anterior ocelli, in profile two times as long as face; frontal vitta almost linear in front of ocellar triangle and strongly widened anteriorly; parafacial nearly parallel-sided, about three times as wide as (nearly about 2.5 as wide as in profile) 1^st^ postpedicel; face weakly concave, very weakly carinate on upper median portion between base of antenna, lower margin of face warped forwards; gena about 2/7 (about 2/7 in profile) of eye height; occiput flattened on upper half, slightly bulged on lower half. Inner vertical setae fine and long, hair-like, slightly longer, not different from postocular setae row; ocellar seta hair-like and fine 1/5-2/7 times as long as eye height; 7-9 frontal setae, 2 upper setae finer and shorter, lowest seta nearly up to upper level of scape; fronto-orbital and parafacial plate with dense and long hairs and with a row of setae on inner side of parafacial; facial ridge with 1-2 setae on lower 1/5; vibrissal inserted at level of lower margin; 9-10 subvibrissal setae, which is at most 1/2 as long as vibrissal; postocular setae close to posterior eye margin, long and directed forward on upper 1/3. Base of antenna nearly level with middle of eye; antenna short, 0.6-0.66 times as long as face; pedicel with a seta which is nearly as long as 1^st^ postpedicel; 1^st^ postpedicel about twice as long as wide and 1.5 times as long as pedicel; arista short pubescent, about 1.5 times as long as pedicel and 1^st^ postpedicel together, thickened on basal 1/5–1/6, 2^nd^ aristomere at most 1.5 times as long as wide. Prementum 4-4.5 times as long as wide, about twice as long as genal height; palpus about twice as long as 1^st^ postpedicel.

Thorax shiny black in ground colour, with very thin greyish pruinosity on postpronotal lobe, presutural area of scutum and pro-epimeron. Presutural area of scutum with 3 narrow black median vittae and a pair of black lateral triangular markings. Hairs black, rather dense and erect on scutum and scutellum and dense and long on pleura; 2-3 presutural and 2-3 postsutural acrostichal setae; 2 presutural and 3 postsutural dorsocentral setae; 2 postsutural intra-alar seta; pre-alar seta strong, longer than second supra-alar seta and notopleural seta, but shorter than first supra-alar seta; 3-4 pairs of reclinate and strong marginal scutellar setae, a pair of weak lateral scutellar setae or absent; apical scutellar setae strong and crossed, 1.75-2 times as long as scutellum; a pairs of discal scutellar setae near apex, about as long as scutellum. Two katepisteral setae and anterior one weak；1 upper anterior and a row of (8-9) posterior anepisternal setae; 1 weak anepisternal seta, thin and long; anatergite hairy.

Wing hyaline, weakly tinged with pale brown, more strongly tinged on basal portion; tegula dark brown, basicosta reddish-yellow; lower calypter yellowish-white, fringe yellowish; halters brown to dark, except apex of halters reddish-yellow. Costal spine as long as crossvein r-m, base of vein R_4+5_ with 3-4 fine setulae dorsally and ventrally. Relative lengths of costal sectors 2^nd^, 3^rd^ and 4^th^ approximately as 0.75:2:1; vein M from dM-Cu crossvein to its bend 2.5-3 times distance between the bend and wing hind margin.

Legs black or dark brown with pulvilli yellowish, claws and pulvilli longer than 5^th^ tarsomere. Fore tibia with 4-5 anteriodorsal setae, upper 3-4 short and weak, pre-apical anterodorsal seta longer than pre-apical dorsal seta; mid-tibia with 3 anterodorsal seta, upper one short, 3-4 posterior and 1 ventral seta; hind tibia with a row of irregular anterodorsal, 3 or 4 of them strong, 2 posterodorsal and 2-3 ventral setae; 1 pre-apical anterodorsal seta slightly shorter than 1 pre-apical dorsal seta; pre-apical anteroventral seta distinct longer than pre-apical posteroventral seta.

Abdomen long ovate, shiny black without pruinosity. Mid-dorsal excavation of syntergite 1+2 only extending to its basal half, with 2 median marginal setae and 3-4 lateral marginal setae and with some lateral discal setae; 3^rd^ tergite usually with 2 median marginal setae and 3-4 lateral marginal setae, 2 median discal setae, without lateral discal seta; 4^th^ tergite with a row of marginal setae, 2 median discal setae; 5^th^ tergite separately with a row of marginal and discal setae and 2 anterior median discal setae. Male terminalia as Fig. 4. Sternite 5 nearly triangular, the depth of V-shaped median cleft of sternite 5 about 1/2 of the sternite length, posterior lobe bluntly rounded apically and inner margin slightly pointed at apex. Aedeagal apodeme fork-like at apex, pregonite long, thin at middle, postgonite pointed and hook-like bent downwards, distiphallus long sclerotised at middle and membranous with some rows of minute spinules on surface. In caudal view, cerci slender and apex pointed, surstylus short and thin, slightly blunt at apex. In lateral view, cerci thin and its apex pointed and slightly bent backwards, surstylus slender and slightly rounded at apex.

**Female**. Differing from male as follows: Frons wide, vertex 0.38-0.4 of head width; frontal vitta at middle 1.3 times as wide as fronto-orbital plate; 6 frontal setae and with 2 proclinate orbital setae; 1 outward prevertical seta, about as long as occellar, but weaker than orbital seta; inner vertical setae strong, distinct different from postocular setae row; claws and pulvilli shorter than 5^th^ tarsomere.

#### Diagnosis

Frons about as wide as antenna. Parafacial hairy on whole length. Palpi dark brown, pedicel and legs black, 3 postsutural dorsocentral setae. Pre-alar seta about as long as hind supra-alar seta, 2 katepisternal setae. Basicosta reddish-yellow. Mid-tibia with 2-5 anterodorsal setae. Abdomen without pruinosity and marking, mid-dorsal excavation of syntergite 1+2 not extending to its posterior margin, tergite 3 with 2-4 marginal setae.

#### Etymology

Specific epithet from the Latin adjective setal (= setose) plus noun face, in reference to one of the diagnostic characters of this species, in reference to the haired parafacial, which is a diagnostic feature of this species.

#### Distribution

Palaearctic China (Qinghai)

#### Remarks.

This species is similar to *M.dispar* (Fallén), but it is distinguished from the latter in having wider frons, as wide as antenna, reddish-yellow basicosta, pre-alar seta about as long as hind supra-alar seta, 2 katepisternal setae, abdomen without pruinosity and marking.

### Macquartia sichuanensis

Zhang & Zhang sp. nov.

811E3DF0-08CE-52E0-81FE-C3A5AEEAA0E6

C8C63DA3-99DF-4A08-929B-546A5EE9128E

#### Materials

- **Type status:** Holotype. **Occurrence:** recordedBy: Zhang XZ; individualCount: 1; sex: male; lifeStage: adult; occurrenceID: 2F135E9F-254F-5C35-8229-FF89E906B01E; **Taxon:** scientificName: Macquartiasichuanensis; **Location:** country: China; stateProvince: Sichuan; locality: Wolong Nature Reserve, Wenchuan County; verbatimElevation: 1920 m; verbatimCoordinates: 30.33°N, 103.42°E; decimalLatitude: 30.33; decimalLongitude: 103.42; **Identification:** identifiedBy: Zhang Chun-tian; dateIdentified: 2022; **Event:** samplingProtocol: sweeping; eventDate: 30/07/1983; **Record Level:** collectionCode: Insects
- **Type status:** Paratype. **Occurrence:** recordedBy: Huang KR; individualCount: 1; sex: male; lifeStage: adult; occurrenceID: 1C86DC03-7A85-5516-AD88-F12FB8251AFA; **Taxon:** scientificName: Macquartiasichuanensis; **Location:** country: China; stateProvince: Sichuan; locality: Jiulao cave, Emei Mountains; verbatimElevation: 1800-1900 m; verbatimCoordinates: 29.36°N, 103.29°E; decimalLatitude: 29.36; decimalLongitude: 103.29; **Identification:** identifiedBy: Zhang Chun-tian; dateIdentified: 2022; **Event:** samplingProtocol: sweeping; eventDate: 08/05/1957; **Record Level:** collectionCode: Insects
- **Type status:** Paratype. **Occurrence:** recordedBy: Zhang Chun-tian; individualCount: 1; sex: male; lifeStage: adult; occurrenceID: 665968BB-6DCF-5620-8B66-2229924FE0D9; **Taxon:** scientificName: Macquartiasichuanensis; **Location:** country: China; stateProvince: Chongqing; locality: Yintiaoling Nature Reserve, Wuxi County; verbatimElevation: 1780-2070 m; verbatimCoordinates: 31.40°N, 109.88°E; decimalLatitude: 31.4; decimalLongitude: 109.88; **Identification:** identifiedBy: Zhang Chun-tian; dateIdentified: 2022; **Event:** samplingProtocol: sweeping; eventDate: 14-16/08/2022; **Record Level:** collectionCode: Insects

#### Description

Body length 7.3-8.0 mm (Fig. 5a, b).

**Male**. This species is similar to *M.barkamensis* sp. n. in appearance, but it is different from the latter in head (Fig. 5c, d) having dark brown antenna, except for base of 1^st^ postpedicel brown, palpus dark brown. Frons about as wide as anterior ocellar width, parafacial nearly as wide as 1^st^ postpedicel, genal height about 1/7-1/8 of eye height; 8-9 frontal setae, lowest seta nearly up to upper level of pedicel; parafacial bare; facial ridge with short seta-like hairs on lower 1/5-1/3. Base of antenna nearly level with 1/3 lower of eye; antenna at least 4/5 as long as face, 1^st^ postpedicel about 3-3.5 times as long as wide and about 3 times as long as pedicel; arista long plumose, aristal hairs as long as 1^st^ postpedicel width. Prementum short, about twice as long as wide.

Thorax covered with brownish-grey white pruinosity on lateral surfaces of presutural scutum, anepisternum and katepisternum. Two presutural and 3 postsutural dorsocentral setae; pre-alar seta absent, 2 supra-alar setae longer than notopleural seta; apical scutellar setae crossed, about 1.5 times as long as scutellum. Proepisternum hairy, 1 upper anterior and a row of (6-7) posterior anepisternal setae, 3 katepisteral setae. Wing with tegula and basicosta dark brown; lower calypter brownish to brown. Costal spine at least as long as, or nearly, crossvein r-m, base of vein R_4+5_ with 3-7 fine setulae dorsally and ventrally, relative lengths of costal sectors 2^nd^, 3^rd^ and 4^th^ approximately as 2:6:3; vein M from dM-Cu crossvein to its bend about three times distance between the bend and hind margin of wing. Legs dark brown. Fore tibia with a row of 7-9 short anteriodorsal setae (sometimes 3-4 of them stronger), pre-apical anterodorsal seta distinctly weaker than pre-apical dorsal seta; mid-tibia with 4 posterodorsal setae, ventral seta absent; hind tibia with 6-8 anterodorsal, 3-4 of them long, 2-4 ventral setae, lower two stronger; 3 pre-apical dorsal setae equal in length.

Abdomen ovate with shiny black, Mid-dorsal excavation of syntergite 1+2 only extending to its basal half, antero-lateral suraces of 3^rd^ and 4^th^ tergite covered with greyish-white pruinosity. Syntergite 1+2 with two pairs of lateral marginal and with 2 lateral discal setae, median marginal seta absent; 3^rd^ tergite with a row of (8-10) marginal setae, a pair of median discal setae and 1-2 pairs of lateral discal setae. Male terminalia as Fig. 6.

**Female**. Unknown.

#### Diagnosis

Parafacial bare, palpi dark. Two presutural and 3 postsutural dorsocentral setae, 3 katepisternal setae. Lower calyptrae divergent from scutellum. Mid-tibia with 1 anterodorsal seta. Abdomen with sparse pruinosity, syntergite 1+2 medially excavate to base, with a pair of lateral marginal setae, without median marginal and discal seta, 3^rd^ tergite with a complete row of marginal and 2 median discal setae, 4^th^ tergite separately with a row of discal and marginal setae.

#### Etymology

The specific epithet is taken from a locality of this species, i.e. Sichuan, China.

#### Distribution

Oriental China (Chongqing, Sichuan).

#### Remarks.

This species is similar with *M.barkamensis* sp. n. in appearance, but it is distinguished from the latter in having bare parafacial, dark brown palpi, 3 katepisternal setae, syntergite 1+2 with two pairs of lateral marginal setae, 3rd tergite with a complete row of marginal setae.

### Macquartia brunneisquama

Zhang & Li 2022

2C176C04-E4C9-5F04-B416-A883FF478BF0

#### Materials

- **Type status:** Holotype. **Occurrence:** individualCount: 1; sex: male; lifeStage: adult; occurrenceID: 6D630FAF-2473-59B0-8815-C7794B38DF76; **Taxon:** scientificName: Macquartiabrunneisquama; **Location:** country: China; stateProvince: Hubei; locality: Dalaoling, Yichang County; **Identification:** identifiedBy: Zhang Chun-Tian; dateIdentified: 2022; **Event:** samplingProtocol: Malaise traps; **Record Level:** collectionCode: Insects
- **Type status:** Paratype. **Occurrence:** individualCount: 5; sex: male; lifeStage: adult; occurrenceID: 5F2D44EB-B1E6-5FF8-92BA-7DB6AA3CBFD3; **Taxon:** scientificName: Macquartiabrunneisquama; **Location:** country: China; stateProvince: Hubei; locality: Dalaoling, Yichang County; **Identification:** identifiedBy: Zhang Chun-Tian; dateIdentified: 2022; **Event:** samplingProtocol: Malaise traps; **Record Level:** collectionCode: Insects
- **Type status:** Paratype. **Occurrence:** recordedBy: Li Jun-Jian; individualCount: 1; sex: female; lifeStage: adult; occurrenceID: C9B32DA3-AE7B-5701-B872-39B8CAB4D59C; **Taxon:** scientificName: Macquartiabrunneisquama; **Location:** country: China; stateProvince: Qinghai; locality: Zhamashi, Qilian Mountains; verbatimElevation: 3050 m; verbatimCoordinates: 38.90°N, 99.59°E; decimalLatitude: 38.9; decimalLongitude: 99.59; **Identification:** identifiedBy: Zhang Chun-Tian; dateIdentified: 2022; **Event:** samplingProtocol: sweeping; eventDate: 19/08/2019; **Record Level:** collectionCode: Insects

#### Diagnosis

Male frons as wide as anterior ocellus, parafacial usually bare, ocelli yellow. Gena about 2/5 in profile as high as eye height. Palpi reddish-yellow. Two presutural and 3 postsutural dorsocentral setae. Legs black, mid-tibia with 2 anterodorsal setae. Lower calyptrae yellowish, divergent from scutellum. Mid-dorsal excavation of abdominal syntergite 1+2 not extending to its posterior margin, with a pair of lateral marginal setae, without median marginal seta; 3^rd^ tergite with 2 median marginal and 2 pairs of lateral marginal setae, without median discal seta.

#### Distribution

Oriental China (Hubei), Palaearctic China (Qinghai).

### Macquartia chinensis

Zhang & Li 2022

A58C1BBD-4436-51FE-A8D6-AF57D65481B0

#### Materials

- **Type status:** Holotype. **Occurrence:** individualCount: 1; sex: male; lifeStage: adult; occurrenceID: 1C696E57-0F96-5CD1-B136-53B1A9273A32; **Taxon:** scientificName: Macquartiachinensis; **Location:** country: China; stateProvince: Hubei; locality: Dalaoling, Yichang County; **Identification:** identifiedBy: Zhang Chun-Tian; dateIdentified: 2022; **Event:** samplingProtocol: Malaise traps; **Record Level:** collectionCode: Insects
- **Type status:** Paratype. **Occurrence:** individualCount: 6; sex: male; lifeStage: adult; occurrenceID: CB2ABF7F-0A3A-5ABF-8C62-28485D65F775; **Taxon:** scientificName: Macquartiachinensis; **Location:** country: China; stateProvince: Hubei; locality: Dalaoling, Yichang County; **Identification:** identifiedBy: Zhang Chun-Tian; dateIdentified: 2022; **Event:** samplingProtocol: Malaise traps; **Record Level:** collectionCode: Insects
- **Type status:** Paratype. **Occurrence:** individualCount: 2; sex: female; lifeStage: adult; occurrenceID: 63C2DA60-D585-5BF6-90BD-7068C941D4D3; **Taxon:** scientificName: Macquartiachinensis; **Location:** country: China; stateProvince: Hubei; locality: Dalaoling, Yichang County; **Identification:** identifiedBy: Zhang Chun-Tian; dateIdentified: 2022; **Event:** samplingProtocol: Malaise traps; **Record Level:** collectionCode: Insects
- **Type status:** Paratype. **Occurrence:** recordedBy: Li Xin-Yi, Liang Hou-Can; individualCount: 3; sex: male; lifeStage: adult; occurrenceID: D958EFA1-9F97-5A0D-9E32-5F1081AA19B9; **Taxon:** scientificName: Macquartiachinensis; **Location:** country: China; stateProvince: Liaoning; locality: Wandianzi, Qingyuan County; verbatimElevation: 600-720 m; verbatimCoordinates: 41.97°N, 115.29°E; verbatimLatitude: 41.97; verbatimLongitude: 115.29; **Identification:** identifiedBy: Zhang Chun-Tian; dateIdentified: 2022; **Event:** samplingProtocol: sweeping; eventDate: 21/07/2016; **Record Level:** collectionCode: Insects
- **Type status:** Paratype. **Occurrence:** recordedBy: Liang Hou-Can; individualCount: 1; sex: female; lifeStage: adult; occurrenceID: CCB80120-A547-5601-98E1-DFBD8E59C9E5; **Taxon:** scientificName: Macquartiachinensis; **Location:** country: China; stateProvince: Yunnan; locality: Yulong Snow Mountains, Lijiang; verbatimElevation: 2685-3107 m; verbatimCoordinates: 26. 90°N, 100.17°E; verbatimLatitude: 26.9; verbatimLongitude: 100.17; **Identification:** identifiedBy: Zhang Chun-Tian; dateIdentified: 2022; **Event:** samplingProtocol: sweeping; eventDate: 20/07/2017; **Record Level:** collectionCode: Insects
- **Type status:** Paratype. **Occurrence:** occurrenceID: A784CBD7-1077-5ECF-BE7E-36C7F8505DD0

#### Diagnosis

Parafacial bare. Lower calyptrae divergent from scutellum. Frons of male 1/3 as wide as postpedicel or as wide as anterior ocellus. Pedicel reddish-yellow, 3 (seldom 2) presutural and 3 postsutural dorsocentral setae, mid-tibia with 1 anterodorsal seta; abdominal 3rd and 4^th^ tergites without median discal seta. Mid-tibia with 2-5 anterodorsal setae. Abdominal 3^rd^ tergite with 2-4 marginal setae.

#### Distribution

Oriental China (Hubei, Yunnan), Palaearctic China (Liaoning).

### Macquartia flavifemorata

Zhang & Li 2022

6A3E0439-9620-590F-A816-CEB44D688B7B

#### Materials

- **Type status:** Holotype. **Occurrence:** individualCount: 1; sex: male; lifeStage: adult; occurrenceID: 3324FB15-028E-5781-B82C-E961FC73FC87; **Taxon:** scientificName: Macquartiaflavifemorata; **Location:** country: China; stateProvince: Hubei; locality: Dalaoling, Yichang County; **Identification:** identifiedBy: Zhang Chun-Tian; dateIdentified: 2022; **Event:** samplingProtocol: Malaise traps; **Record Level:** collectionCode: Insects
- **Type status:** Paratype. **Occurrence:** individualCount: 9; sex: female; lifeStage: adult; occurrenceID: AEBC09E7-4C8B-5727-B34F-268B82B8C829; **Taxon:** scientificName: Macquartiaflavifemorata; **Location:** country: China; stateProvince: Hubei; locality: Dalaoling, Yichang County; **Identification:** identifiedBy: Zhang Chun-Tian; dateIdentified: 2022; **Event:** samplingProtocol: Malaise traps; **Record Level:** collectionCode: Insects

#### Diagnosis

Parafacial bare. Pedicel and basal half of postpedicel yellow, palpi, basicosta and legs reddish-yellow, aristal hairs at least more than diameter of aristal base. Thoracic scutum with grey pruinosity, 3 presutural and 3 postsutural dorsocentral setae, 2 katepisternal setae. Lower calyptrae divergent from scutellum. Abdomen black, with sparse pruinosity or absent, mid-dorsal excavation of syntergite 1+2 extending or nearly to its posterior margin, without median marginal seta, 3^rd^ tergite with 2 median marginal setae, 3^rd^ and 4^th^ tergites without median discal setae in both sexes.

#### Distribution

Oriental China (Hubei), Palaearctic China (Liaoning).

### Macquartia flavipedicel

Zhang & Li 2022

2E7C3080-1D3F-5375-A931-9ED99F87B3FE

#### Materials

- **Type status:** Holotype. **Occurrence:** individualCount: 1; sex: male; lifeStage: adult; occurrenceID: 0104B732-47EB-541A-8135-A9041A568827; **Taxon:** scientificName: Macquartiaflavipedicel; **Location:** country: China; stateProvince: Hubei; locality: Dalaoling, Yichang County; **Identification:** identifiedBy: Zhang Chun-Tian; dateIdentified: 2022; **Event:** samplingProtocol: Malaise traps; **Record Level:** collectionCode: Insects
- **Type status:** Paratype. **Occurrence:** individualCount: 18; sex: male; lifeStage: adult; occurrenceID: 6B11EB90-2668-5A27-BA4F-98A9C9441943; **Taxon:** scientificName: Macquartiaflavipedicel; **Location:** country: China; stateProvince: Hubei; locality: Dalaoling, Yichang County; **Identification:** identifiedBy: Zhang Chun-Tian; dateIdentified: 2022; **Event:** samplingProtocol: Malaise traps; **Record Level:** collectionCode: Insects
- **Type status:** Paratype. **Occurrence:** individualCount: 1; sex: female; lifeStage: adult; occurrenceID: D1D95639-439B-5B0C-9A31-6AF44BF5B25B; **Taxon:** scientificName: Macquartiaflavipedicel; **Location:** country: China; stateProvince: Hubei; locality: Dalaoling, Yichang County; **Identification:** identifiedBy: Zhang Chun-Tian; dateIdentified: 2022; **Event:** samplingProtocol: Malaise traps; **Record Level:** collectionCode: Insects

#### Diagnosis

Parafacial bare, at most with 3-4 hairs below lowest frontal seta. Ocelli whitish-yellow. Pedicel and basal half of postpedicel yellow, aristal hairs at least more than diameter of aristal base. Palpi reddish-yellow. Thoracic scutum with grey pruinosity, 3 postsutural dorsocentral setae, 2 katepisternal setae. Lower calyptrae divergent from scutellum, basicosta of wing dark brown, legs black. Abdomen black, mid-dorsal excavation of syntergite 1+2 extending or nearly to its posterior margin, with a pair of lateral marginal setae, without median marginal seta, 3^rd^ and 4^th^ tergites without median discal seta in both sexes.

#### Distribution

Oriental China (Hubei).

### Macquartia chalconota

(Meigen 1824)

CF4BE1A3-0E4F-5A6A-8BF0-2C7E06F11FF0

#### Diagnosis

Frons of male 1/3-2/3 as wide as postpedicel, Parafacial bare, at most hairy on upper half. Palpi reddish-yellow, seldom dark at apex. Thoxax with 3 postsutural dorsocentral setae. Lower calyptrae not divergent from scutellum. Mid-tibia with 2-5 anterodorsal setae, hind tibia with 2 pre-apical dorsal setae. Abdomen of female covered with covered greyish pruinosity, with gleaming markings. Mid-dorsal excavation of abdominal syntergite 1+2 not extending to its posterior margin, without median marginal seta, 3rd tergite with 2-4 marginal setae.

#### Distribution

Palaearctic China (Heilongjiang, Inner Mongolia, Ningxia, Qinghai), Europe, Russia, Transcaucasus.

#### Hosts

Coleoptera, Chrysomelidae: *Chrysolinaherbacea* Duftschmid, *Entomoscelisadonidis* Pallas, Chrysomelidae sp. (Tschorsnig 2017: 264).

### Macquartia dispar

(Fallén 1820)

4808D0C3-314D-5DC2-9BA1-96CE3466A249

#### Materials

- **Type status:** Other material. **Occurrence:** recordedBy: Xue Wan-Qi; individualCount: 16; sex: male; lifeStage: adult; occurrenceID: EAC5B11A-AE2B-526A-BC54-9C84ED48B9D5; **Taxon:** scientificName: Macquartiadispar; **Location:** country: China; stateProvince: Liaoning; locality: Mt. Tiecha, Benxi; **Identification:** identifiedBy: Zhang Chun-Tian; dateIdentified: 2022; **Event:** samplingProtocol: sweeping; eventDate: 05/09/1980; **Record Level:** collectionCode: Insects
- **Type status:** Other material. **Occurrence:** recordedBy: Zhang Chun-Tian; individualCount: 1; sex: male; lifeStage: adult; occurrenceID: 3BA88ACF-9F86-5174-A6A1-2F1038DA8425; **Taxon:** scientificName: Macquartiadispar; **Location:** country: China; stateProvince: Liaoning; locality: Nandian, Benxi; verbatimElevation: 915 m; verbatimCoordinates: 41.29°N, 124.33°E; decimalLatitude: 41.29; decimalLongitude: 124.33; **Identification:** identifiedBy: Zhang Chun-Tian; dateIdentified: 2022; **Event:** samplingProtocol: sweeping; eventDate: 16/05/2012; **Record Level:** collectionCode: Insects
- **Type status:** Other material. **Occurrence:** recordedBy: Xue Wan-Qi; individualCount: 2; sex: male; lifeStage: adult; occurrenceID: D6225F79-7CA6-51C8-845E-C711F9D733A9; **Taxon:** scientificName: Macquartiadispar; **Location:** country: China; stateProvince: Liaoning; locality: Hupu, Caohezhang, Benxi; verbatimElevation: 1100 m; **Identification:** identifiedBy: Zhang Chun-Tian; dateIdentified: 2022; **Event:** samplingProtocol: sweeping; eventDate: 29/04/2004; **Record Level:** collectionCode: Insects
- **Type status:** Other material. **Occurrence:** recordedBy: Yang FY; individualCount: 1; sex: male; lifeStage: adult; occurrenceID: 04881D69-76EB-5122-AA96-D74796AFC4AC; **Taxon:** scientificName: Macquartiadispar; **Location:** country: China; stateProvince: Nei Mongolia; locality: Yikezhao Pref; verbatimCoordinates: 37.60°N, 102.20°E; decimalLatitude: 37.6; decimalLongitude: 102.2; **Identification:** identifiedBy: Zhang Chun-Tian; dateIdentified: 2022; **Event:** samplingProtocol: sweeping; eventDate: 19/04/2006; **Record Level:** collectionCode: Insects
- **Type status:** Other material. **Occurrence:** recordedBy: Hao Bo; individualCount: 1; sex: female; lifeStage: adult; occurrenceID: 3DADFDAE-4B21-53C8-947D-F3A151641BC8; **Taxon:** scientificName: Macquartiadispar; **Location:** country: China; stateProvince: Qinghai; locality: Guxiangsigou, Menyuan County; verbatimElevation: 2408 m; **Identification:** identifiedBy: Zhang Chun-Tian; dateIdentified: 2022; **Event:** samplingProtocol: sweeping; eventDate: 14/15-07-2019; **Record Level:** collectionCode: Insects
- **Type status:** Other material. **Occurrence:** recordedBy: H. Shima coll. ＆ det; individualCount: 1; sex: male; lifeStage: adult; occurrenceID: 2CA9BEC9-7733-523F-AEA1-DDF1E90C510A; **Taxon:** scientificName: Macquartiadispar; **Location:** country: Japan; stateProvince: Hokkaido; locality: Mts. Daisetsu, Yukomanbetsu; **Identification:** identifiedBy: Zhang Chun-Tian; dateIdentified: 2022; **Event:** samplingProtocol: sweeping; eventDate: 10/13/07/1986; **Record Level:** collectionCode: Insects

#### Diagnosis

Frons twice as wide as aristal base. Parafacial hairy on whole length (Fig. 7). Pedicel and leg black. Three postsutural dorsocentral setae. Pre-alar seta weaker than hind supra-alar seta in famale. Three katepisternal setae. Basicosta dark brown. Mid-tibia with 2-5 anterodorsal setae. Abdomen covered with pruinosity, with trapezoid dark marking, mid-dorsal excavation of syntergite 1+2 not extending to its posterior margin, 3^rd^ tergite with 2-4 marginal setae. Male terminalia (Fig. 8). Sternite 5 nearly square-shaped, base slightly prominent, the depth of V-shaped median cleft of sternite 5 about 1/2 of the sternite, inner margin of posterior lobe short and pointed apically. Pregonite smoothly curved and long, blunt at apex, postgonite parallel-sided with pointed apex. Distiphallus slender. In caudal view, cerci slender, apex pointed, surstylus bent outwards and bluntly rounded at apex. In lateral view, apex of cerci bent backwards, surstylus narrowed and bluntly rounded at apex.

#### Distribution

Palaearctic: China (Liaoning, Ningxia, Qinghai), Mongolia, Iran, Russia (E. Siberia, S. Far East, W. Russia), Europe (Belarus, Czechia, Hungary, Moldova, Poland, Romania, Slovakia, Ukraine, Denmark, Finland, Sweden, Andorra, Bulgaria, Croatia, Greece, Italy, Portugal, Serbia, Spain, Austria, Belgium, France, Germany, Netherlands), Transcaucasia.

#### Hosts

Coleoptera, Chrysomelidae: *Chrysolinaamericana* Linnaeus, *Chrysolinasanguinolenta* Linnaeus, *Colaphussophiae* Schaller, *Timarchagoettingensis* Linnaeus, *Timarcha* sp. (Tschorsnig 2017: 265).

### Macquartia grisea

(Fallén 1810)

C1938822-7CF2-5EA3-BDB7-C1D609911B8A

#### Materials

- **Type status:** Other material. **Occurrence:** recordedBy: Zhang Chun-Tian; individualCount: 1; sex: male; lifeStage: adult; occurrenceID: 5911C7E7-6D43-5990-A05F-5499B1326B21; **Taxon:** scientificName: Macquartiagrisea; **Location:** country: China; stateProvince: Sichuan; locality: Maoniu Valley, Bamei to Danba; verbatimElevation: 2888 m; verbatimCoordinates: 30.61°N, 101.69°E; decimalLatitude: 30.61; decimalLongitude: 101.69; **Identification:** identifiedBy: Zhang Chun-Tian; dateIdentified: 2022; **Event:** samplingProtocol: sweeping; eventDate: 11/08/2017; **Record Level:** collectionCode: Insects

#### Diagnosis

Parafacial hairy only on upper half. Palpi reddish-yellow. Three postsutural dorsocentral setae. Three katepisternal setae. Mid-tibia with 1 anterodorsal seta. Lower calyptrae divergent from scutellum. Abdomen with grey or yellowish-grey pruinosity, without black marking, mid-dorsal excavation of syntergite 1+2 not extending to its posterior margin, with 2 median marginal setae, 3^rd^ tergite with 2-4 median discal setae and a complete row of marginal setae.

#### Distribution

Palaearctic: China (Sichuan), Iran, Europe (British Isles, Czechia, Hungary, Poland, Romania, Slovakia, Ukraine, Denmark, Sweden, Bosnia & Herzegovina, Bulgaria, Italy, Portugal, Serbia, Spain, Austria, Belgium, France, Germany, Netherlands, Switzerland), Russia (W. Russia), Transcaucasia (Georgia).

#### Hosts

Coleoptera, Chrysomelidae: *Chrysolinafastuosa* Scopoli, *Chrysolinaoricalcia* Müller, *Chrysolinasanguinolenta* Linnaeus (Tschorsnig, 2017: 265).

### Macquartia macularis

Villeneuve 1926

73F5204D-AE88-559B-B57B-51F6B5418B0F

#### Materials

- **Type status:** Other material. **Occurrence:** recordedBy: Zhi Yan; individualCount: 1; sex: female; lifeStage: adult; occurrenceID: E2501BD7-27CC-54DB-90FF-FBA34D48C03C; **Taxon:** scientificName: Macquartiamacularis; **Location:** country: China; stateProvince: Liaoning; locality: Laotuding, Huanren; verbatimElevation: 500-1320 m; verbatimCoordinates: 41.19°N, 124.68°E; decimalLatitude: 41.19; decimalLongitude: 124.68; **Identification:** identifiedBy: Zhang Chun-Tian; dateIdentified: 2022, missing; **Event:** samplingProtocol: sweeping; eventDate: 01/05/2005; **Record Level:** collectionCode: Insects

#### Diagnosis

Parafacial hairy on whole length. Palpi reddish-yellow. Three postsutural dorsocentral setae, 2 katepisternal setae. Mid-tibia with 1 anterodorsal seta. Lower calyptrae divergent from scutellum. Abdomen covered with grey pruinosity, syntergite 1+2, 3^rd^ and 4^th^ tergites each with a pair of black markings, mid-dorsal excavation of syntergite 1+2 not extending to its posterior margin, without median marginal seta, with 1-2 pairs of lateral marginal setae, 3^rd^ and 4^th^ tergites each with 2 (weak) median discal and 2-3 pairs of lateral discal setae, 3^rd^ tergite with a complete row of marginal setae.

#### Distribution

Palaearctic: China (Liaoning, Ningxia, Shanxi, Sichuan), Mongolia, Europe (Albania，Czechia, Slovakia, Ukraine, Switzerland), North Africa (Morocco, Tunisia).

### Macquartia nudigena

Mesnil 1972

2AB6D6D0-1277-56CB-919C-2792F8E9379A

#### Materials

- **Type status:** Other material. **Occurrence:** recordedBy: Xue Wan-Qi; individualCount: 1; sex: male; lifeStage: adult; occurrenceID: B6DECDCF-B521-5AF0-A22C-EA88C7BDBBA2; **Taxon:** scientificName: Macquartianudigena; **Location:** country: China; stateProvince: Liaoning; locality: Nandian, Benxi; **Identification:** identifiedBy: Zhang Chun-Tian; dateIdentified: 2022; **Event:** samplingProtocol: sweeping; eventDate: 01/04/1977; **Record Level:** collectionCode: Insects
- **Type status:** Other material. **Occurrence:** recordedBy: Xue Wan-Qi; individualCount: 2; sex: male; lifeStage: adult; occurrenceID: 52BFBA08-93B7-5220-BFFA-5987DDEAB5EE; **Taxon:** scientificName: Macquartianudigena; **Location:** country: China; stateProvince: Liaoning; locality: Caohekou, Benxi; **Identification:** identifiedBy: Zhang Chun-Tian; dateIdentified: 2022; **Event:** samplingProtocol: sweeping; eventDate: 19/05/1980; **Record Level:** collectionCode: Insects
- **Type status:** Other material. **Occurrence:** recordedBy: Xue Wan-Qi; individualCount: 1; sex: female; lifeStage: adult; occurrenceID: 14642B21-B534-5D44-8D8F-B5FE79228D5B; **Taxon:** scientificName: Macquartianudigena; **Location:** country: China; stateProvince: Liaoning; locality: Caohekou, Benxi; **Identification:** identifiedBy: Zhang Chun-Tian; dateIdentified: 2022; **Event:** samplingProtocol: sweeping; eventDate: 19/05/1980; **Record Level:** collectionCode: Insects
- **Type status:** Other material. **Occurrence:** recordedBy: Zhang Chun-Tian, Yao Zhi-Yuan, Ge Zhen-Ping, Zhi Yan; individualCount: 7; sex: male; lifeStage: adult; occurrenceID: B979C29F-D9A6-5292-88FE-7130958E5608; **Taxon:** scientificName: Macquartianudigena; **Location:** country: China; stateProvince: Liaoning; locality: Sanjiazi, Xiuyan; verbatimElevation: 400 m; **Identification:** identifiedBy: Zhang Chun-Tian; dateIdentified: 2022; **Event:** samplingProtocol: sweeping; eventDate: 17/05/2007; **Record Level:** collectionCode: Insects
- **Type status:** Other material. **Occurrence:** recordedBy: Zhang Chun-Tian, Yao Zhi-Yuan, Ge Zhen-Ping, Zhi Yan; individualCount: 1; sex: female; lifeStage: adult; occurrenceID: 61ABB377-CD86-5EB5-8521-04F4C5F6DF63; **Taxon:** scientificName: Macquartianudigena; **Location:** country: China; stateProvince: Liaoning; locality: Mt. Yaoshan, Xiuyan; verbatimElevation: 400 m; **Identification:** identifiedBy: Zhang Chun-Tian; dateIdentified: 2022; **Event:** samplingProtocol: sweeping; eventDate: 17/05/2007; **Record Level:** collectionCode: Insects
- **Type status:** Other material. **Occurrence:** recordedBy: Hao Jing; individualCount: 1; sex: female; lifeStage: adult; occurrenceID: B86E0DDB-4060-5500-9D67-B13674E80765; **Taxon:** scientificName: Macquartianudigena; **Location:** country: China; stateProvince: Liaoning; locality: Sanjiazi, Xiuyan; verbatimElevation: 400-800 m; **Identification:** identifiedBy: Zhang Chun-Tian; dateIdentified: 2022; **Event:** samplingProtocol: sweeping; eventDate: 18/05/2007; **Record Level:** collectionCode: Insects
- **Type status:** Other material. **Occurrence:** recordedBy: Xue Wan-Qi; individualCount: 1; sex: female; lifeStage: adult; occurrenceID: 3C330B96-6616-5D0F-80BF-89448538B3AD; **Taxon:** scientificName: Macquartianudigena; **Location:** country: China; stateProvince: Liaoning; locality: Mt. Qipan, Shenyang; **Identification:** identifiedBy: Zhang Chun-Tian; dateIdentified: 2022; **Event:** samplingProtocol: sweeping; eventDate: 29/04/2008; **Record Level:** collectionCode: Insects
- **Type status:** Other material. **Occurrence:** recordedBy: Yang ZQ; individualCount: 1; sex: female; lifeStage: adult; occurrenceID: E02638C9-DF62-550E-9AFE-92D4BE0C7C4B; **Taxon:** scientificName: Macquartianudigena; **Location:** country: China; stateProvince: Liaoning; locality: Beiling, Shenyang; **Identification:** identifiedBy: Zhang Chun-Tian; dateIdentified: 2022; **Event:** samplingProtocol: sweeping; eventDate: 19/05/2006; **Record Level:** collectionCode: Insects
- **Type status:** Other material. **Occurrence:** recordedBy: Zhang Chun-Tian, Yao Zhi-Yuan; individualCount: 15; sex: male; lifeStage: adult; occurrenceID: 2E28212A-C749-5E85-BAD5-0D8C8D398F8E; **Taxon:** scientificName: Macquartianudigena; **Location:** country: China; stateProvince: Liaoning; locality: Wenquansi to Mt. Guanyin, Benxi; verbatimElevation: 340-530 m; **Identification:** identifiedBy: Zhang Chun-Tian; dateIdentified: 2022; **Event:** samplingProtocol: sweeping; eventDate: 01/05/2008; **Record Level:** collectionCode: Insects
- **Type status:** Other material. **Occurrence:** recordedBy: Zhang Chun-Tian, Yao Zhi-Yuan; individualCount: 1; sex: female; lifeStage: adult; occurrenceID: A8102AEA-BFE4-5978-9CD5-16CFF083EDF0; **Taxon:** scientificName: Macquartianudigena; **Location:** country: China; stateProvince: Liaoning; locality: Wenquansi to Mt. Guanyin, Benxi; verbatimElevation: 340-530 m; **Identification:** identifiedBy: Zhang Chun-Tian; dateIdentified: 2022; **Event:** samplingProtocol: sweeping; eventDate: 01/05/2008; **Record Level:** collectionCode: Insects
- **Type status:** Other material. **Occurrence:** recordedBy: Zhang Chun-Tian, Fan Hong-Ye, Cui Le; individualCount: 32; sex: male; lifeStage: adult; occurrenceID: FC2B3686-4718-513F-B406-BA6B402E751D; **Taxon:** scientificName: Macquartianudigena; **Location:** country: China; stateProvince: Liaoning; locality: Mt.Tiecha, Benxi; verbatimElevation: 915 m; **Identification:** identifiedBy: Zhang Chun-Tian; dateIdentified: 2022; **Event:** samplingProtocol: sweeping; eventDate: 15-16/05/2012; **Record Level:** collectionCode: Insects
- **Type status:** Other material. **Occurrence:** recordedBy: Zhang Chun-Tian, Fan Hong-Ye, Cui Le; individualCount: 1; sex: female; lifeStage: adult; occurrenceID: 39E1487D-5864-5258-9A14-50845ECBBA1E; **Taxon:** scientificName: Macquartianudigena; **Location:** country: China; stateProvince: Liaoning; locality: Mt.Tiecha, Benxi; verbatimElevation: 915 m; **Identification:** identifiedBy: Zhang Chun-Tian; dateIdentified: 2022; **Event:** samplingProtocol: sweeping; eventDate: 15-16/05/2012; **Record Level:** collectionCode: Insects
- **Type status:** Other material. **Occurrence:** recordedBy: Wang XL; individualCount: 1; sex: male; lifeStage: adult; occurrenceID: 36A7CD88-20FB-5471-98C5-D6ABF67EA131; **Taxon:** scientificName: Macquartianudigena; **Location:** country: China; stateProvince: Liaoning; locality: Mt. Wunv, Huanren; verbatimElevation: 800 m; **Identification:** identifiedBy: Zhang Chun-Tian; dateIdentified: 2022; **Event:** samplingProtocol: sweeping; eventDate: 31/05/2013; **Record Level:** collectionCode: Insects

#### Diagnosis

Frons of male about 2/3 as wide as postpedicel (Fig. 9). Parafacial bare. Palpi reddish-yellow. Thoracic scutum and abdomen with grey pruinosity, 3 postsutural dorsocentral setae. Lower calyptrae divergent from scutellum. Fore claw of male longer than or as long as 5^th^ tarsomere. Mid-tibia with 2-5 anterodorsal setae, hind tibia with 2 pre-apical dorsal setae. Mid-dorsal excavation of abdominal syntergite 1+2 not extending to its posterior margin, with 2 strong median marginal setae in male and weak setae in female, 4^th^ tergite with 2-4 median discal setae. Male terminalia (Fig. 10). Sternite 5 nearly square-shaped, base flattened, the depth of V-shaped median cleft about 2/3 of the sternite, inner margin of posterior lobe long and pointed apically. Pregonite sclerotised and smoothly curved and short, postgonite parallel-sided, wide and short with slightly rounded tip, distiphallus narrowed posteriorly and membranous apical part. In caudal view, cerci thick and slightly shortened, apex slightly pointed, surstylus slightly bent inwards and bluntly rounded at apex. In lateral view, apex of cerci slightly bent backwards, surstylus bluntly rounded at apex

#### Distribution

Palaearctic: China (Liaoning, Inner Mongolia, Ningxia), Europe (British Isles, Czechia, Estonia, Hungary, Poland, Slovakia, Denmark, Finland, Norway, Sweden, Italy, Serbia, Austria, Belgium, France, Germany, Switzerland), Russia (S. Far East, W. Russia).

### Macquartia pubiceps

(Zetterstedt 1845)

E2FEB2D7-F21C-53BE-9760-7683CB233484

#### Materials

- **Type status:** Other material. **Occurrence:** recordedBy: Wang Qiang; individualCount: 1; sex: female; lifeStage: adult; occurrenceID: E40DA88F-C1A2-55FD-8FA4-44BAC8B861C8; **Taxon:** scientificName: Macquartiapubiceps; **Location:** country: China; stateProvince: Anhui; locality: Huangshan; verbatimElevation: 150-500 m; **Identification:** identifiedBy: Zhang Chun-Tian; dateIdentified: 2022; **Event:** samplingProtocol: sweeping; eventDate: 07/05/2015; **Record Level:** collectionCode: Insects
- **Type status:** Other material. **Occurrence:** recordedBy: Dong Rui-Qing; individualCount: 1; sex: male; lifeStage: adult; occurrenceID: 871CCD42-EDB3-5BD7-AEB2-78DDCE5695D4; **Taxon:** scientificName: Macquartiapubiceps; **Location:** country: China; stateProvince: Chongqing; locality: Guanshan Forestry Station, Wuxi; verbatimElevation: 1832 m; verbatimCoordinates: 31.29˚N, 109.43˚E; decimalLatitude: 21.39; decimalLongitude: 109.43; **Identification:** identifiedBy: Zhang Chun-Tian; dateIdentified: 2022; **Event:** samplingProtocol: sweeping; eventDate: 21/06/2022; **Record Level:** collectionCode: Insects
- **Type status:** Other material. **Occurrence:** recordedBy: Zhang Chun-Tian; individualCount: 1; sex: female; lifeStage: adult; occurrenceID: D1A1E402-F561-564C-8298-5E6532E3E6BE; **Taxon:** scientificName: Macquartiapubiceps; **Location:** country: China; stateProvince: Fujian; locality: Wuyi Mountains, Xizhou Village; verbatimElevation: 650 m; **Identification:** identifiedBy: Zhang Chun-Tian; dateIdentified: 2022; **Event:** samplingProtocol: sweeping; eventDate: 28/05/2021; **Record Level:** collectionCode: Insects
- **Type status:** Other material. **Occurrence:** recordedBy: Zhang Chun-Tian, Fu Chao; individualCount: 3; sex: male; lifeStage: adult; occurrenceID: FCC47162-06BB-5377-9D2A-FB569379D89B; **Taxon:** scientificName: Macquartiapubiceps; **Location:** country: China; stateProvince: Guangxi; locality: Dayao Mountains, Jinxiu; verbatimElevation: 850-1300 m; **Identification:** identifiedBy: Zhang Chun-Tian; dateIdentified: 2022; **Event:** samplingProtocol: sweeping; eventDate: 11-18/05/2009, 14-17/05/2011; **Record Level:** collectionCode: Insects
- **Type status:** Other material. **Occurrence:** recordedBy: Zhang Chun-Tian, Fu Chao; individualCount: 1; sex: female; lifeStage: adult; occurrenceID: 82F8A76E-1521-52BC-BF2F-51ED20F2F577; **Taxon:** scientificName: Macquartiapubiceps; **Location:** country: China; stateProvince: Guangxi; locality: Dayao Mountains, Jinxiu; verbatimElevation: 850-1300 m; **Identification:** identifiedBy: Zhang Chun-Tian; dateIdentified: 2022; **Event:** samplingProtocol: sweeping; eventDate: 11-18/05/2009, 14-18/05/2011; **Record Level:** collectionCode: Insects
- **Type status:** Other material. **Occurrence:** recordedBy: Wang Min; individualCount: 1; sex: male; lifeStage: adult; occurrenceID: 769D9B50-5D6F-58B2-A2E4-F466C9CF7A88; **Taxon:** scientificName: Macquartiapubiceps; **Location:** country: China; stateProvince: Guangdong; locality: Nanling Mountains; verbatimElevation: 950-1040 m; **Identification:** identifiedBy: Zhang Chun-Tian; dateIdentified: 2022; **Event:** samplingProtocol: sweeping; eventDate: 31/05/2007; **Record Level:** collectionCode: Insects
- **Type status:** Other material. **Occurrence:** recordedBy: Li Xin-Yi; individualCount: 1; sex: male; lifeStage: adult; occurrenceID: DF199AF7-35AF-5918-938D-A4237D26A975; **Taxon:** scientificName: Macquartiapubiceps; **Location:** country: China; stateProvince: Guangdong; locality: Protection and Management Station; verbatimCoordinates: 24.55˚N, 113.10˚E; decimalLatitude: 24.55; decimalLongitude: 113.1; **Identification:** identifiedBy: Zhang Chun-Tian; dateIdentified: 2022; **Event:** samplingProtocol: sweeping; eventDate: 11/05/2021; **Record Level:** collectionCode: Insects
- **Type status:** Other material. **Occurrence:** recordedBy: Zhang Chun-Tian, Wang Qiang; individualCount: 4; sex: male; lifeStage: adult; occurrenceID: 2956C939-ED59-5171-A3A2-FD04BED7EACA; **Taxon:** scientificName: Macquartiapubiceps; **Location:** country: China; stateProvince: Guizhou; locality: Maolan, Libo; verbatimElevation: 850-1080m; **Identification:** identifiedBy: Zhang Chun-Tian; dateIdentified: 2022; **Event:** samplingProtocol: sweeping; eventDate: 20-21/05/2011; **Record Level:** collectionCode: Insects
- **Type status:** Other material. **Occurrence:** recordedBy: Zhang Chun-Tian, Wang Qiang; individualCount: 1; sex: female; lifeStage: adult; occurrenceID: 2994EC53-CB6A-53DD-8694-F6AD3F1FB0AB; **Taxon:** scientificName: Macquartiapubiceps; **Location:** country: China; stateProvince: Guizhou; locality: Maolan, Libo; verbatimElevation: 850-1080m; **Identification:** identifiedBy: Zhang Chun-Tian; dateIdentified: 2022; **Event:** samplingProtocol: sweeping; eventDate: 20-21/05/2011; **Record Level:** collectionCode: Insects
- **Type status:** Other material. **Occurrence:** recordedBy: Wang Qiang; individualCount: 3; sex: female; lifeStage: adult; occurrenceID: DFDF20D5-6BF3-5678-8D49-4188589770C6; **Taxon:** scientificName: Macquartiapubiceps; **Location:** country: China; stateProvince: Guizhou; locality: Leigong Mountains, Kaili; verbatimElevation: 1200-2100m; **Identification:** identifiedBy: Zhang Chun-Tian; dateIdentified: 2022; **Event:** samplingProtocol: sweeping; eventDate: 24/05/2011; **Record Level:** collectionCode: Insects
- **Type status:** Other material. **Occurrence:** recordedBy: Fu Chao; individualCount: 1; sex: male; lifeStage: adult; occurrenceID: 69A39C92-D51E-52DD-802A-BA74BAE3ED68; **Taxon:** scientificName: Macquartiapubiceps; **Location:** country: China; stateProvince: Hainan; locality: Yinggezui; verbatimElevation: 630 m; **Identification:** identifiedBy: Zhang Chun-Tian; dateIdentified: 2022; **Event:** samplingProtocol: sweeping; eventDate: 08/05/2011; **Record Level:** collectionCode: Insects
- **Type status:** Other material. **Occurrence:** recordedBy: Zhang Chun-Tian; individualCount: 1; sex: male; lifeStage: adult; occurrenceID: 38C83ABA-4231-5F42-B7DE-10D74A46DDD4; **Taxon:** scientificName: Macquartiapubiceps; **Location:** country: China; stateProvince: Hubei; locality: Tongshan, Xianning; verbatimElevation: 400-1300 m; **Identification:** identifiedBy: Zhang Chun-Tian; dateIdentified: 2022; **Event:** samplingProtocol: sweeping; eventDate: 16/06/2014; **Record Level:** collectionCode: Insects
- **Type status:** Other material. **Occurrence:** recordedBy: Zhang Chun-Tian; individualCount: 1; sex: male; lifeStage: adult; occurrenceID: 5A7608ED-FC6E-5721-A502-E8E49702BD90; **Taxon:** scientificName: Macquartiapubiceps; **Location:** country: China; stateProvince: Liaoning; locality: Mt. Tiecha, Benxi; verbatimElevation: 915 m; **Identification:** identifiedBy: Zhang Chun-Tian; dateIdentified: 2022; **Event:** samplingProtocol: sweeping; eventDate: 16/05/2012; **Record Level:** collectionCode: Insects
- **Type status:** Other material. **Occurrence:** recordedBy: Zhao Zhe; individualCount: 1; sex: male; lifeStage: adult; occurrenceID: 456B59CA-933A-5C2A-874C-151474090761; **Taxon:** scientificName: Macquartiapubiceps; **Location:** country: China; stateProvince: Liaoning; locality: Mt. Tiecha, Benxi; verbatimElevation: 912 m; **Identification:** identifiedBy: Zhang Chun-Tian; dateIdentified: 2022; **Event:** samplingProtocol: sweeping; eventDate: 03/07/2010; **Record Level:** collectionCode: Insects
- **Type status:** Other material. **Occurrence:** recordedBy: Liu Jia-Yu, Zhao Zhe; individualCount: 2; sex: male; lifeStage: adult; occurrenceID: 44EFE8BC-AD95-595F-9FA2-88EFEF545C2D; **Taxon:** scientificName: Macquartiapubiceps; **Location:** country: China; stateProvince: Liaoning; locality: Laotudingzi, Huanren; verbatimElevation: 500-1350 m; **Identification:** identifiedBy: Zhang Chun-Tian; dateIdentified: 2022; **Event:** samplingProtocol: sweeping; eventDate: 02/04/2009, 31/05/2006; **Record Level:** collectionCode: Insects

#### Diagnosis

Parafacial bare, at most with 3-4 hairs below lowest frontal seta, ocelli red (Fig. 11). Pedicel and basal half of postpedicel black. Palpi reddish-yellow. Aristal hairs at least more than diameter of aristal base. Thoracic scutum with grey pruinosity, 4 postsutural dorsocentral setae. Two katepisternal setae. Basicosta reddish-yellow. Lower calyptrae divergent from scutellum. Abdomen black, with sparse pruinosity or without pruinosity, syntergite 1+2 mid-dorsally excavated extending or nearly to its posterior margin, with a pair of lateral marginal setae, without median marginal seta; 3^rd^ tergite with 2 median marginal setae and 2 median discal setae in male and female, 4^th^ tergite with a row of marginal setae and 2 to a row of discal setae in male and 2 setae in female. Male terminalia (Fig. 12). Base of sternite 5 distinctly convex, the depth of V-shaped median cleft of sternite 5 about 2/5 of the sternite, posterior lobe bluntly rounded apically and inner margin slightly pointed at apex. Pregonite short, postgonite long and thin, bluntly rounded at apex, distiphallus membranous apical part. In caudal view, cerci slender and apex pointed, surstylus slender and slightly blunt at apex. In lateral view, cerci slender and its apex slightly bent backwards, surstylus slender and slightly pointed at apex.

#### Distribution

Oriental: China (Anhui, Chongqing, Fujian, Guizhou, Guangdong, Guangxi, Hainan, Hubei). Palaearctic: China (Hebei, Liaoning, Inner Mongolia, Ningxia, Shaanxi, Shanxi), Japan (Honshū), Russia (S. Far East, W. Russia), Europe (British Isles, Czechia, Hungary, Lithuania, Poland, Romania, Slovakia, Ukraine, Sweden, Italy, Portugal, Spain, Austria, Belgium, France, Germany, Netherlands), Transcaucasia.

#### Hosts

Coleoptera, Chrysomelidae: *Chrysomelabifrons* (Chao et al. 1998: 2044).

### Macquartia tenebricosa

(Meigen 1824)

9CDD5C02-FE12-5572-8965-76B3FD25AA7E

#### Materials

- **Type status:** Other material. **Occurrence:** recordedBy: Zhou Yuan-Ye; individualCount: 7; sex: male; lifeStage: adult; occurrenceID: E86080E0-6654-56A8-A5F5-535DC5BE21C2; **Taxon:** scientificName: Macquartiatenebricosa; **Location:** country: China; stateProvince: Gansu; locality: Shifogou, Lanzhou; verbatimElevation: 2100-2300 m; **Identification:** identifiedBy: Zhang Chun-Tian; dateIdentified: 2022; **Event:** samplingProtocol: sweeping; eventDate: 18/07/2009; **Record Level:** collectionCode: Insects
- **Type status:** Other material. **Occurrence:** recordedBy: Zhou Yuan-Ye, Zhao Zhe; individualCount: 3; sex: male; lifeStage: adult; occurrenceID: 41690CCA-E5BA-5410-8389-5A6AB44B4F55; **Taxon:** scientificName: Macquartiatenebricosa; **Location:** country: China; stateProvince: Gansu; locality: Xiaotulugou, Yongdeng; verbatimElevation: 2300-3100 m; **Identification:** identifiedBy: Zhang Chun-Tian; dateIdentified: 2022; **Event:** samplingProtocol: sweeping; eventDate: 21/07/2009; **Record Level:** collectionCode: Insects
- **Type status:** Other material. **Occurrence:** recordedBy: Zhou Yuan-Ye, Zhao Zhe; individualCount: 1; sex: female; lifeStage: adult; occurrenceID: 9511C27D-3F2D-5AD2-A819-C1E591D931B0; **Taxon:** scientificName: Macquartiatenebricosa; **Location:** country: China; stateProvince: Gansu; locality: Xiaotulugou, Yongdeng; verbatimElevation: 2300-3101 m; **Identification:** identifiedBy: Zhang Chun-Tian; dateIdentified: 2022; **Event:** samplingProtocol: sweeping; eventDate: 21/07/2009; **Record Level:** collectionCode: Insects
- **Type status:** Other material. **Occurrence:** recordedBy: Wang Shi-Di; individualCount: 1; sex: male; lifeStage: adult; occurrenceID: BD05BFBB-4CFD-5531-A7C9-D062EA65BFCB; **Taxon:** scientificName: Macquartiatenebricosa; **Location:** country: China; stateProvince: Gansu; locality: Datulugou, Yongdeng; verbatimElevation: 2300-3000 m; **Identification:** identifiedBy: Zhang Chun-Tian; dateIdentified: 2022; **Event:** samplingProtocol: sweeping; eventDate: 22/07/2009; **Record Level:** collectionCode: Insects
- **Type status:** Other material. **Occurrence:** recordedBy: Wang Shi-Di, Zhao Zhe; individualCount: 2; sex: male; lifeStage: adult; occurrenceID: 31A84FBD-F79A-591A-A7EF-0A94418E0E48; **Taxon:** scientificName: Macquartiatenebricosa; **Location:** country: China; stateProvince: Gansu; locality: Guanegou, Dangchang; verbatimElevation: 2200-2525 m; **Identification:** identifiedBy: Zhang Chun-Tian; dateIdentified: 2022; **Event:** samplingProtocol: sweeping; eventDate: 31/07/2009; **Record Level:** collectionCode: Insects
- **Type status:** Other material. **Occurrence:** recordedBy: Zhou Yuan-Ye; individualCount: 1; sex: male; lifeStage: adult; occurrenceID: F4F44036-3CD3-58A3-A2A9-A299EF56112D; **Taxon:** scientificName: Macquartiatenebricosa; **Location:** country: China; stateProvince: Hebei; locality: Xiaowutai Mountains; verbatimElevation: 1600 m; **Identification:** identifiedBy: Zhang Chun-Tian; dateIdentified: 2022; **Event:** samplingProtocol: sweeping; eventDate: 22/04/2009; **Record Level:** collectionCode: Insects
- **Type status:** Other material. **Occurrence:** recordedBy: Sun Qi; individualCount: 1; sex: male; lifeStage: adult; occurrenceID: 2A38EAFE-6B94-5C89-9650-E4CD09485711; **Taxon:** scientificName: Macquartiatenebricosa; **Location:** country: China; stateProvince: Hebei; locality: Mt. Heilong, Zhangjiakou; verbatimElevation: 1331-1526 m; **Identification:** identifiedBy: Zhang Chun-Tian; dateIdentified: 2022; **Event:** samplingProtocol: sweeping; eventDate: 07/07/2016; **Record Level:** collectionCode: Insects
- **Type status:** Other material. **Occurrence:** recordedBy: Sun Qi; individualCount: 2; sex: male; lifeStage: adult; occurrenceID: 01DEB666-7508-5DC3-986D-8256765F6FCB; **Taxon:** scientificName: Macquartiatenebricosa; **Location:** country: China; stateProvince: Hebei; locality: Liaoheyuan; verbatimElevation: 1247 m; **Identification:** identifiedBy: Zhang Chun-Tian; dateIdentified: 2022; **Event:** samplingProtocol: sweeping; eventDate: 14/07/2016; **Record Level:** collectionCode: Insects
- **Type status:** Other material. **Occurrence:** recordedBy: Zhang Chun-Tian, Hou Peng; individualCount: 6; sex: male; lifeStage: adult; occurrenceID: 28B07F30-6DD2-5D81-B4FA-C754803C292A; **Taxon:** scientificName: Macquartiatenebricosa; **Location:** country: China; stateProvince: Heilongjiang; locality: Mudanfeng Forest Park, Mudanjiang; verbatimElevation: 250-1100 m; **Identification:** identifiedBy: Zhang Chun-Tian; dateIdentified: 2022; **Event:** samplingProtocol: sweeping; eventDate: 19-20/07/2013; **Record Level:** collectionCode: Insects
- **Type status:** Other material. **Occurrence:** recordedBy: Xue Wan-Qi; individualCount: 1; sex: male; lifeStage: adult; occurrenceID: 9052869C-28CC-5AEE-92CA-4F8BC18F2E86; **Taxon:** scientificName: Macquartiatenebricosa; **Location:** country: China; stateProvince: Jilin; locality: Tonghua, Hani; **Identification:** identifiedBy: Zhang Chun-Tian; dateIdentified: 2022; **Event:** samplingProtocol: sweeping; eventDate: 01/07/1973; **Record Level:** collectionCode: Insects
- **Type status:** Other material. **Occurrence:** recordedBy: Xue Wan-Qi; individualCount: 1; sex: male; lifeStage: adult; occurrenceID: 774DF5AB-04D4-560C-BA39-5B3E578D5C90; **Taxon:** scientificName: Macquartiatenebricosa; **Location:** country: China; stateProvince: Liaoning; locality: Shuangshuidong, Shajianzi, Huanren; **Identification:** identifiedBy: Zhang Chun-Tian; dateIdentified: 2022; **Event:** samplingProtocol: sweeping; eventDate: 23/07/1974; **Record Level:** collectionCode: Insects
- **Type status:** Other material. **Occurrence:** recordedBy: Ge Zhen-Ping; individualCount: 1; sex: male; lifeStage: adult; occurrenceID: 486AFEAE-7254-513C-8368-42A3B751B8DB; **Taxon:** scientificName: Macquartiatenebricosa; **Location:** country: China; stateProvince: Liaoning; locality: Baishilazi, Kuandian; verbatimElevation: 400-500 m; **Identification:** identifiedBy: Zhang Chun-Tian; dateIdentified: 2022; **Event:** samplingProtocol: sweeping; eventDate: 18/08/2007; **Record Level:** collectionCode: Insects
- **Type status:** Other material. **Occurrence:** recordedBy: Zhang Chun-Tian; individualCount: 12; sex: male; lifeStage: adult; occurrenceID: BE54FC0E-0C6E-50CC-91F9-DC273200D3B0; **Taxon:** scientificName: Macquartiatenebricosa; **Location:** country: China; stateProvince: Liaoning; locality: Mt. Daheishan, Jianchang; verbatimElevation: 600-1100 m; **Identification:** identifiedBy: Zhang Chun-Tian; dateIdentified: 2022; **Event:** samplingProtocol: sweeping; eventDate: 25-26/04/2010; **Record Level:** collectionCode: Insects
- **Type status:** Other material. **Occurrence:** recordedBy: Zhang Chun-Tian; individualCount: 1; sex: female; lifeStage: adult; occurrenceID: E1CD69EA-F502-5DF1-AB0E-D682959D0493; **Taxon:** scientificName: Macquartiatenebricosa; **Location:** country: China; stateProvince: Liaoning; locality: Mt. Daheishan, Jianchang; verbatimElevation: 600-1100 m; **Identification:** identifiedBy: Zhang Chun-Tian; dateIdentified: 2022; **Event:** samplingProtocol: sweeping; eventDate: 25-26/04/2010; **Record Level:** collectionCode: Insects
- **Type status:** Other material. **Occurrence:** recordedBy: Chi Yu; individualCount: 3; sex: male; lifeStage: adult; occurrenceID: 07A0F74F-5AF1-5D2B-A50E-62A07188DA53; **Taxon:** scientificName: Macquartiatenebricosa; **Location:** country: China; stateProvince: Liaoning; locality: Mt. Tiecha, Benxi; verbatimElevation: 912 m; **Identification:** identifiedBy: Zhang Chun-Tian; dateIdentified: 2022; **Event:** samplingProtocol: sweeping; eventDate: 03/07/2010; **Record Level:** collectionCode: Insects
- **Type status:** Other material. **Occurrence:** recordedBy: Zhang Chun-Tian; individualCount: 1; sex: male; lifeStage: adult; occurrenceID: 1609448D-A69B-5FC0-9C69-809FCAFDE2FA; **Taxon:** scientificName: Macquartiatenebricosa; **Location:** country: China; stateProvince: Nei Mongolia; locality: Daxinganling Mountains, Jiagedaqi; verbatimElevation: 480-550 m; **Identification:** identifiedBy: Zhang Chun-Tian; dateIdentified: 2022; **Event:** samplingProtocol: sweeping; eventDate: 22/07/2013; **Record Level:** collectionCode: Insects
- **Type status:** Other material. **Occurrence:** recordedBy: Wang Ming-Fu; individualCount: 1; sex: male; lifeStage: adult; occurrenceID: 9A993BC8-E757-5E9A-8715-CCCA532E686E; **Taxon:** scientificName: Macquartiatenebricosa; **Location:** country: China; stateProvince: Ningxia; locality: Liupan Mountains, Heshangpu, Jingyuan; verbatimElevation: 2000 m; **Identification:** identifiedBy: Zhang Chun-Tian; dateIdentified: 2022; **Event:** samplingProtocol: sweeping; eventDate: 23/04/2008; **Record Level:** collectionCode: Insects

#### Diagnosis

Frons 1/7-1/8 of eye in wide, frontal vitta narrower than parafrontalia, parafacial bare. Palpi reddish-yellow seldom dark at apex (Fig. 13). Three katepisternal setae. Lower calyptrae not divergent from scutellum. Mid-tibia with 2-5 anterodorsal setae, hind tibia with 3 pre-apical dorsal setae, middle one weaker. Abdomen with sparse greyish pruinosity in male and female or female black, without pruinosity, mid-dorsal excavation of syntergite 1+2 not extending to its posterior margin, with 2 median marginal and 1-3 lateral marginal setae in both sexes, 3^rd^ and 4^th^ tergites each with 2 median discal and 1-3 pairs of lateral discal setae in both sexes. Male terminalia (Fig. 14). Base of sternite 5 slightly flat, the depth of V-shaped and broad median cleft of sternite 5 about 2/3 of the sternite, posterior lobe bluntly rounded apically. Pregonite short and protruding posteriorly, postgonite long and bluntly rounded, distiphallus distinctly long and sclerotised, membranous apical part. Epiphallus bent upwards and membranous apical part. In caudal view, apical half of cerci narrowed and pointed apically, surstylus slender and slightly bent outwards, slightly blunt at apex. In lateral view, cerci slender and basal half distinctly widened, its apex narrowed and slightly bent backwards, surstylus slender and slightly blunted at apex.

#### Distribution

Palaearctic: China China (Beijing, Gansu, Hebei, Heilongjiang, Jilin, Liaoning, Inner Mongolia, Ningxia, Qinghai, Shanxi, Xizang), Mongolia, Iran, Israel, Russia (E. Siberia, W. Russia), Europe (British Isles, Czechia, Estonia, Hungary, Lithuania, Moldova, Poland, Romania, Slovakia, Ukraine, Denmark, Finland, Norway, Sweden, Albania, Andorra, Bosnia & Herzegovina, Bulgaria, Croatia, Italy, Portugal, Serbia, Slovenia, Spain, Turkey, Austria, Belgium, France, Germany, Netherlands, Switzerland), Transcaucasia.

#### Hosts

Coleoptera, Chrysomelidae: *Ambrostomaquadriimpressum* (Motschulsky), *Chrysolinaamericana* Linnaeus, *C.didymata* Scriba, *C.difficilis* Motschulsky, *C.fastuosa* Scopoli, *C.geminata* Paykull, *C.graminis* Linnaeus, *C.herbacea* Duftschmid, *C.hyperici* Forster, *C.polita* Linnaeus, *C.varians* Schaller, *Chrysolina* sp., Chrysomelidae sp. (Chao et al. 1998: 2044; Tschorsnig 2017: 266).

### Macquartia tessellum

(Meigen 1824)

39EFA79F-1E91-554F-B642-7431837A81AE

#### Diagnosis

Parafacial hairy. Arista almost bare. Palpi reddish-yellow. Four postsutural dorsocentral setae; 3 katepisternal setae. Lower calyptrae not divergent from scutellum. Abdomen black, with grey pruinosity and markings, mid-dorsal excavation of abdominal syntergite 1+2 extending or nearly to its posterior margin, 3^rd^ tergite with a large black marking.

#### Distribution

Oriental: India (Northwest). Palaearctic: Central Asia (Kyrgyzstan, Tajikistan, Turkmenistan), China (Beijing, Liaoning, Qinghai, Xinjiang, Xizang), Europe (British Isles, Andorra, Bosnia & Herzegovina, Bulgaria, Corsica, Croatia, Cyprus, Greece, Italy, Malta, Portugal, Serbia, Spain, Turkey, Austria, France, Germany, Netherlands, Switzerland), Middle East (Iran, Israel, Palestine), North Africa (Canary Islands), Transcaucasia (Armenia).

#### Hosts

Coleoptera, Chrysomelidae: *Chrysolinaamericana* Linnaeus, *C.didymata* Scriba, *C.geminata* Paykull, *C.hyperici* Forster, *C.varians* Schaller, *Colaphuspalaestinus* Achard, *Colaphussophiae* Schaller, *Entomoscelisadonidis* Pallas, *Gonioctenaolivacea* Forster, *Phytodectaolivacea* (Forster) (Chao et al. 1998: 2044; Tschorsnig 2017: 266).

### Macquartia viridana

Robineau-Desvoidy 1863

1F3EFD80-FE64-5AD5-BC17-93F0417EA000

#### Materials

- **Type status:** Other material. **Occurrence:** recordedBy: Cui Le; individualCount: 3; sex: male; lifeStage: adult; occurrenceID: C354605E-91B6-5474-8DA3-C82CA7E251AC; **Taxon:** scientificName: Macquartiaviridana; **Location:** country: China; stateProvince: Liaoning; locality: Tanggou, Benxi; verbatimElevation: 350-450 m; **Identification:** identifiedBy: Zhang Chun-Tian; dateIdentified: 2022; **Event:** samplingProtocol: sweeping; eventDate: 18/07/2009; **Record Level:** collectionCode: Insects
- **Type status:** Other material. **Occurrence:** recordedBy: Li Bing; individualCount: 1; sex: female; lifeStage: adult; occurrenceID: 5A6917D2-7933-54C4-998E-B5196641CF19; **Taxon:** scientificName: Macquartiaviridana; **Location:** country: China; stateProvince: Liaoning; locality: Huangdaigou, Qingyuan; verbatimElevation: 600-800 m; **Identification:** identifiedBy: Zhang Chun-Tian; dateIdentified: 2022; **Event:** samplingProtocol: sweeping; eventDate: 26-31/05/2015; **Record Level:** collectionCode: Insects
- **Type status:** Other material. **Occurrence:** recordedBy: Xue Wan-Qi, Zhou Yuan-Ye; individualCount: 2; sex: male; lifeStage: adult; occurrenceID: 87A059C6-9CB1-5802-A20C-BB4A36D784B6; **Taxon:** scientificName: Macquartiaviridana; **Location:** country: China; stateProvince: Liaoning; locality: Mt. Qipan, Shenyang; verbatimElevation: 230-370 m; **Identification:** identifiedBy: Zhang Chun-Tian; dateIdentified: 2022; **Event:** samplingProtocol: sweeping; eventDate: 29/04/2008, 07/05/2009; **Record Level:** collectionCode: Insects

#### Diagnosis

Frons width at least 1/4 of eye width, frontal vitta about twice as wide as parafrontalia (Fig. 15). Parafacial bare, at most hairy on upper half. Palpi reddish-yellow. Pedicel and tibiae at least reddish-yellow. Femora at least reddish-yellow on ventral apex. Two katepisternal setae. Three postsutural dorsocentral setae. Pre-alar seta about as long as hind supra-alar seta in famale. Mid-tibia with 2-5 anterodorsal setae. Abdomen covered with dense greyish pruinosity and regular gleaming marking, mid-dorsal excavation of syntergite 1+2 not extending to its posterior margin, 3^rd^ tergite with 2-4 marginal setae. Male terminalia (Fig. 16). Base of sternite 5 slightly prominent, the depth of V-shaped median cleft of sternite 5 about 1/2 of the sternite, posterior lobe bluntly rounded apically. Pregonite short and bluntly rounded at apex, postgonite distinctly sclerotised, thin and long, distiphallus tubiform, sclerotised at basal half and membranous on apical half with spinules on surface. In caudal view, cerci narrowed and pointed apically, surstylus thick and slightly bent inwards, slightly blunt at apex. In lateral view, cerci slightly slender and short, apex slightly bent backwards, surstylus thick and slightly blunted at apex.

#### Distribution

Oriental: China (Zhejiang). Palaearctic: China (Liaoning, Inner Mongolia), Europe (British Isles, Czechia, Hungary, Poland, Romania, Slovakia, Ukraine, Bulgaria, Italy, Serbia, Spain, Austria, Belgium, France, Germany, Netherlands, Switzerland), Russia (S. Far East).

#### Hosts

Coleoptera, Chrysomelidae: *Timarcha* sp. (Tschorsnig 2017: 266).

## Identification Keys

### Key to species of *Macquartia* from China

**Table:** 

1	Mid-dorsal excavation of abdominal syntergite 1+2 extending or nearly to its posterior margin. Thorax with 3– 4 postsutural dorsocentral setae. Palpi reddish-yellow.	2
–	Mid-dorsal excavation of abdominal syntergite 1+2 not extending to its posterior margin. Thorax with 3 postsutural dorsocentral setae. Palpi dark brown or reddish-yellow.	5
2	Parafacial bare, at most with 3-4 hairs below lowest frontal seta. 4 or 3 postsutural dorsocentral setae. 2 katepisternal setae. Lower calyptrae divergent from scutellum. Abdomen black, with sparse pruinosity or without pruinosity, syntergite 1+2 with a pair of lateral marginal seta; 3^rd^ tergite with 2 median marginal setae, 4^th^ tergite with a row of marginal setae.	3
–	Parafacial hairy. Arista almost bare; 4 postsutural dorsocentral setae; 3 katepisternal setae. Lower calyptrae not divergent from scutellum. Abdomen black, with grey pruinosity and markings; tergite 3 with large black marking.	M.tessellum (Meigen)
3	Pedicel and basal half of postpedicel dark brown. Thorax with 4 postsutural dorsocentral setae. Basicosta reddish-yellow. 3^rd^ tergite with 2 median discal setae in male and absent in female, 4^th^ tergite with 2 to a row of discal setae in male and 2 setae in female, ocelli red.	*M.pubiceps* (Zetterstedt)
–	Pedicel and basal half of postpedicel yellow. Thorax with 3 postsutural dorsocentral setae. Basicosta reddish-yellow. 3^rd^ and 4^th^ tergites without median discal setae in both sexes, ocelli yellow.	4
4	Basicosta dark brown. Legs black.	*M.flavipedicel* Zhang & Li
–	Basicosta and legs reddish-yellow.	*M.flavifemorata* Zhang & Li
5	Mid-tibia with one anterodorsal seta. Lower calyptrae divergent from scutellum.	6
–	Mid-tibia with 2-5 anterodorsal setae. Abdominal 3^rd^ tergite with 2-4 marginal setae.	10
6	Parafacial bare, at most with 3-4 hairs below lowest frontal seta. Legs black. Syntergite 1+2 without median marginal and with 1-2 lateral marginal setae.	7
–	Parafacial hairy only on upper half or hairy on whole length.	8
7	Pedicel reddish-yellow, 3 presutural and 3 postsutural dorsocentral setae. Abdomen without pruinosity, 3^rd^ tergite with 2 median marginal, 1-2 lateral marginal setae, without discal seta; 4^th^ tergite with a row of marginal setae, without discal seta, 5^th^ tergite with a row of discal and marginal setae.	*M.chinensis* Zhang & Li
–	Pedicel dark brown, 2 presutural and 3 postsutural dorsocentral setae. Abdomen with greyish-white pruinosity anterolateral surfaces on 3^rd^ and 4^th^ tergites, 3^rd^ tergite with a row of marginal and 2 median discal setae; 4^th^ tergite separately with a row of marginal and median discal setae, 5^th^ tergite separately with two rows of discal and a row of marginal setae.	*M.sichuanensis* sp. n.
8	Abdomen without or with sparse pruinosity, 3^rd^ tergite with 4 median marginal and 1 lateral marginal setae and 2 median discal setae; 4^th^ and 5^th^ tergite separately with a row of discal and marginal setae.	*M.barkamensis* sp. n.
–	Abdomen with grey or yellowish-grey pruinosity, 3^rd^ tergite with a complete row of marginal setae.	9
9	Parafacial hairy only on upper half. 3 katepisternal setae. Abdomen without black marking, syntergite 1+2 with 2 median marginal setae, 3^rd^ tergite with 2-4 median discal setae.	*M.grisea* (Fallén)
–	Parafacial hairy on whole length. 2 katepisternal setae. Abdomen covered with grey pruinosity, syntergite 1+2, 3^rd^ and 4^th^ tergites each with a pair of black markings, syntergite 1+2 without median marginal seta, 3^rd^ and 4^th^ tergites each with 2 (weak) median discal setae.	*M.macularis* Villeneuve
10	Parafacial hairy on whole length	11
–	Parafacial bare, at most hairy on upper half.	13
11	Pedicel and legs black.	12
–	Pedicel and tibiae at least reddish-yellow. Femora at least reddish-yellow on ventral apex. 2 katepisternal setae. Frons width at least 1/4 of eye width.	M.viridana Robineau-Desvoidy
12	Basicosta dark brown. 3 katepisternal setae. Frons twice as wide as aristal base. Pre-alar seta weaker than hind supra-alar seta in female. Abdomen covered with pruinosity, with trapezoid dark marking.	*M.dispar* (Fallén)
–	Basicosta reddish-yellow. 2 katepisternal setae. Frons about 4 times as wide as aristal base. Pre-alar seta about as long as hind supra-alar seta. Abdomen without pruinosity and marking.	*Macquartiasetifacies* sp. n.
13	Lower calypters not divergent from scutellum.	14
–	Lower calyptrae divergent from scutellum.	15
14	Hind tibia with 3 pre-apical dorsal setae, middle one weaker; 3 katepisternal setae. Frons 1/7-1/8 of eye width. Parafacial hairy on upper half. Syntergite 1+2 with 2 median marginal and 1-3 lateral marginal setae in both sexes, 3^rd^ and 4^th^ tergites separately with 2 median discal and 1-3 pairs of lateral discal setae in both sexes.	*M.tenebricosa* (Meigen)
–	Hind tibia with 2 pre-apical dorsal setae; syntergite 1+2 without median marginal seta. Abdomen of female covered with greyish pruinosity, with gleaming markings.	*M.chalconota* (Meigen)
15	Hind tibia with 2 preapical dorsal setae. Subapical scutellar seta longer than apical scutellar seta. Costal spine strong. Wing vein r_4+5_ only with setulae on base. Halters yellow. Abdomen with grey pruinosity.	16
–	Hind tibia with 3 pre-apical dorsal setae. Subapical scutellar setae shorter than apical scutellar setae. Wing vein r_4+5_ with setulae at least halfway to crossvein R-M. Halters black or dark brown. Abdomen of female wholly black, without pruinosity, 4^th^ tergite with a complete row of discal setae.	*M.praefica* (Meigen)
16	Abdominal syntergite 1+2 with 2 strong median marginal setae in male and weak setae in female. 4^th^ tergite with 2-4 median discal setae.	*M.nudigena* Mesnil
–	Abdominal syntergite 1+2 without median marginal setae in both sexes; 4^th^ tergites without median discal seta	*M.brunneisqua* Zhang & Li

## Supplementary Material

XML Treatment for Macquartia

XML Treatment for Macquartia barkamensis

XML Treatment for Macquartia setifacies

XML Treatment for Macquartia sichuanensis

XML Treatment for Macquartia brunneisquama

XML Treatment for Macquartia chinensis

XML Treatment for Macquartia flavifemorata

XML Treatment for Macquartia flavipedicel

XML Treatment for Macquartia chalconota

XML Treatment for Macquartia dispar

XML Treatment for Macquartia grisea

XML Treatment for Macquartia macularis

XML Treatment for Macquartia nudigena

XML Treatment for Macquartia pubiceps

XML Treatment for Macquartia tenebricosa

XML Treatment for Macquartia tessellum

XML Treatment for Macquartia viridana

## Figures and Tables

**Figure 1a.**
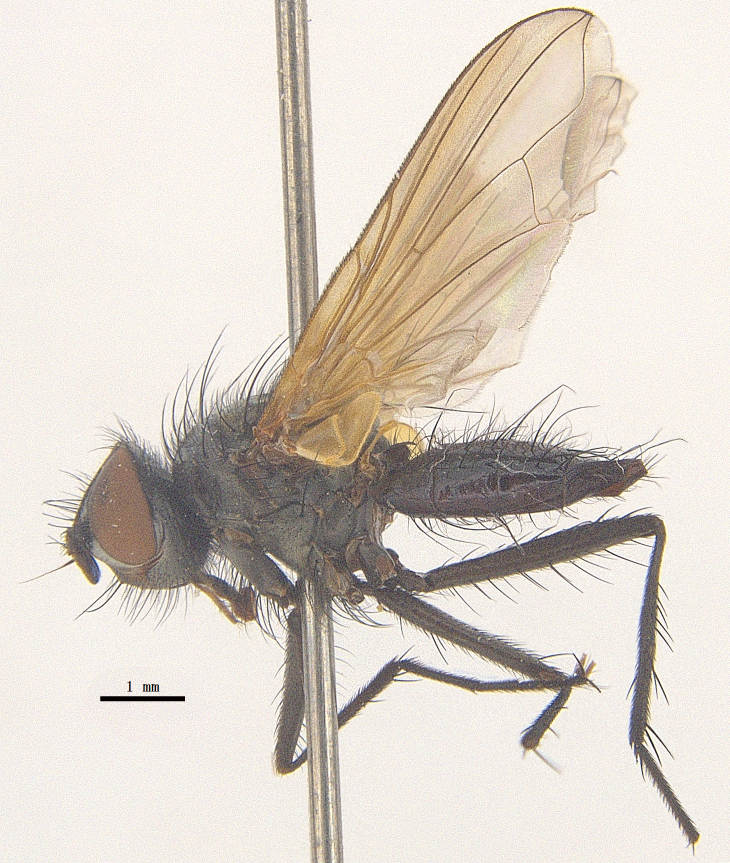
male body in lateral view.

**Figure 1b.**
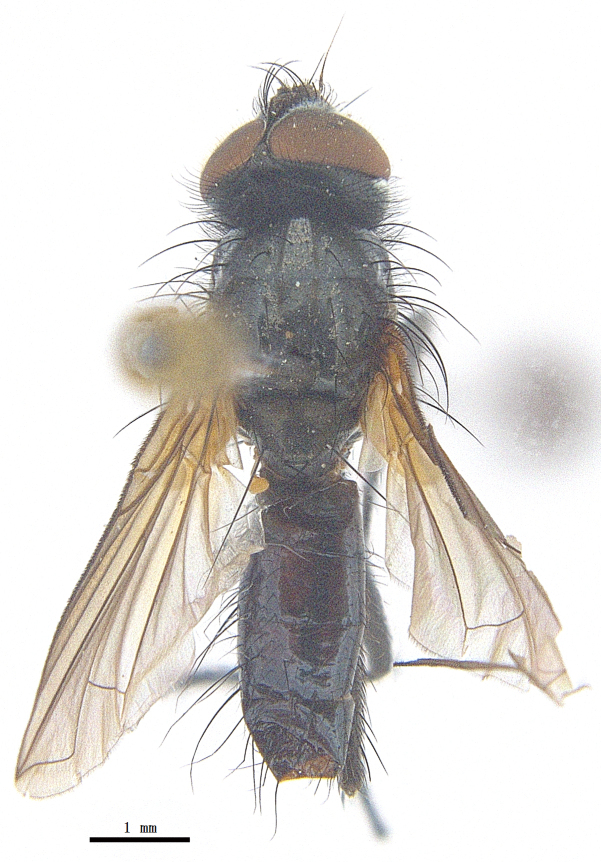
male body in dorsal view.

**Figure 1c.**
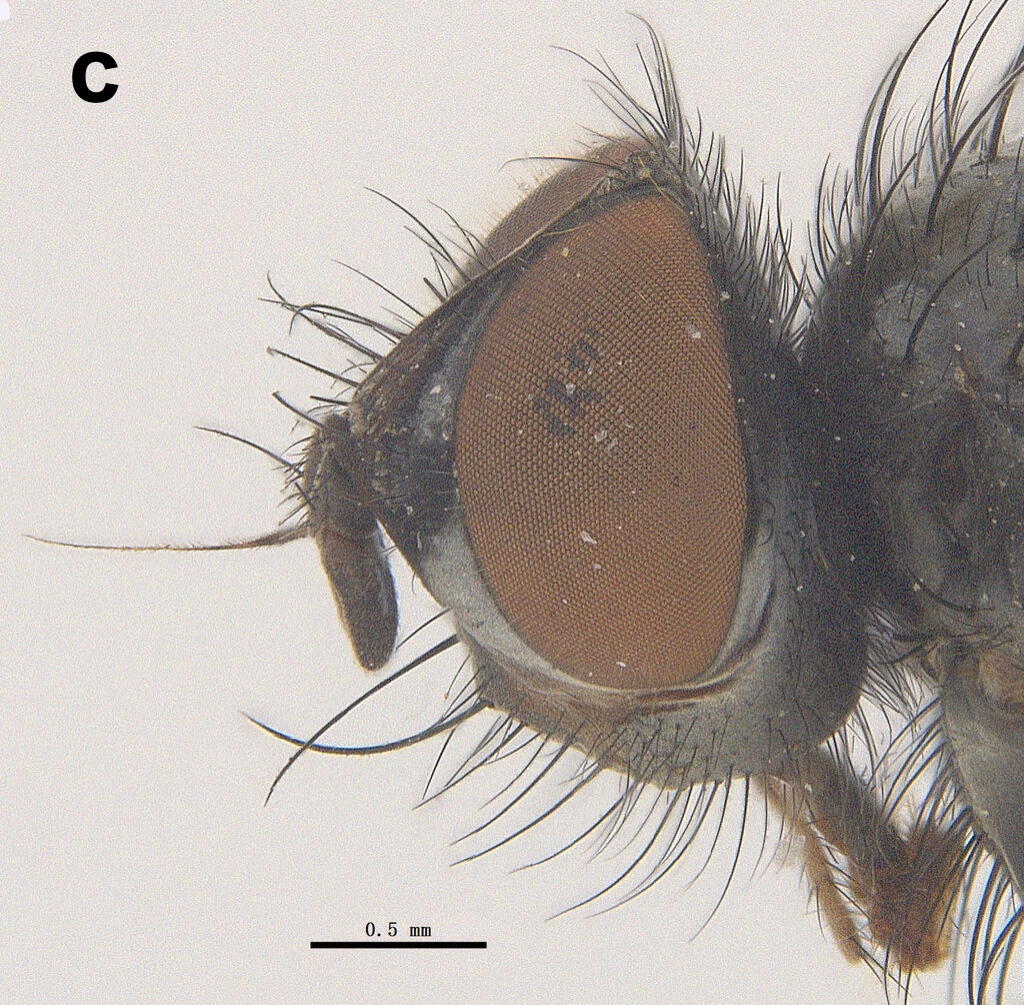
male head in lateral view.

**Figure 1d.**
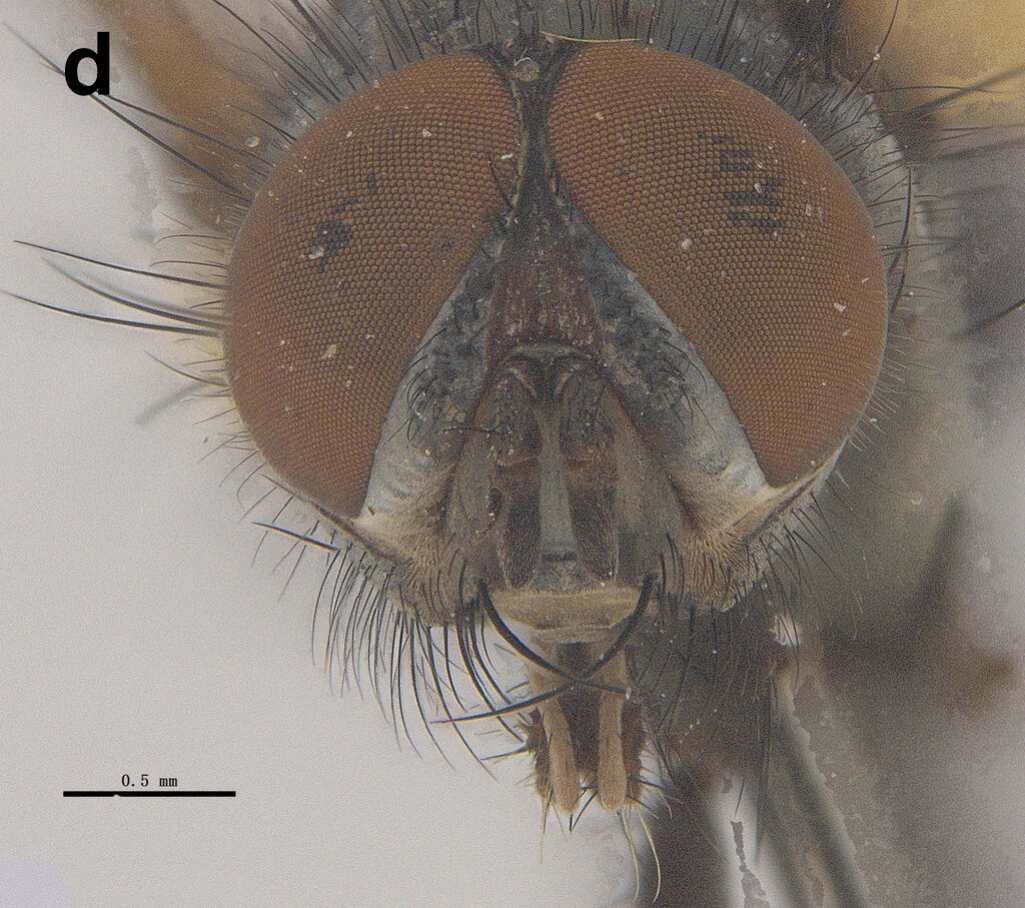
male head in anterior view.

**Figure 2a.**
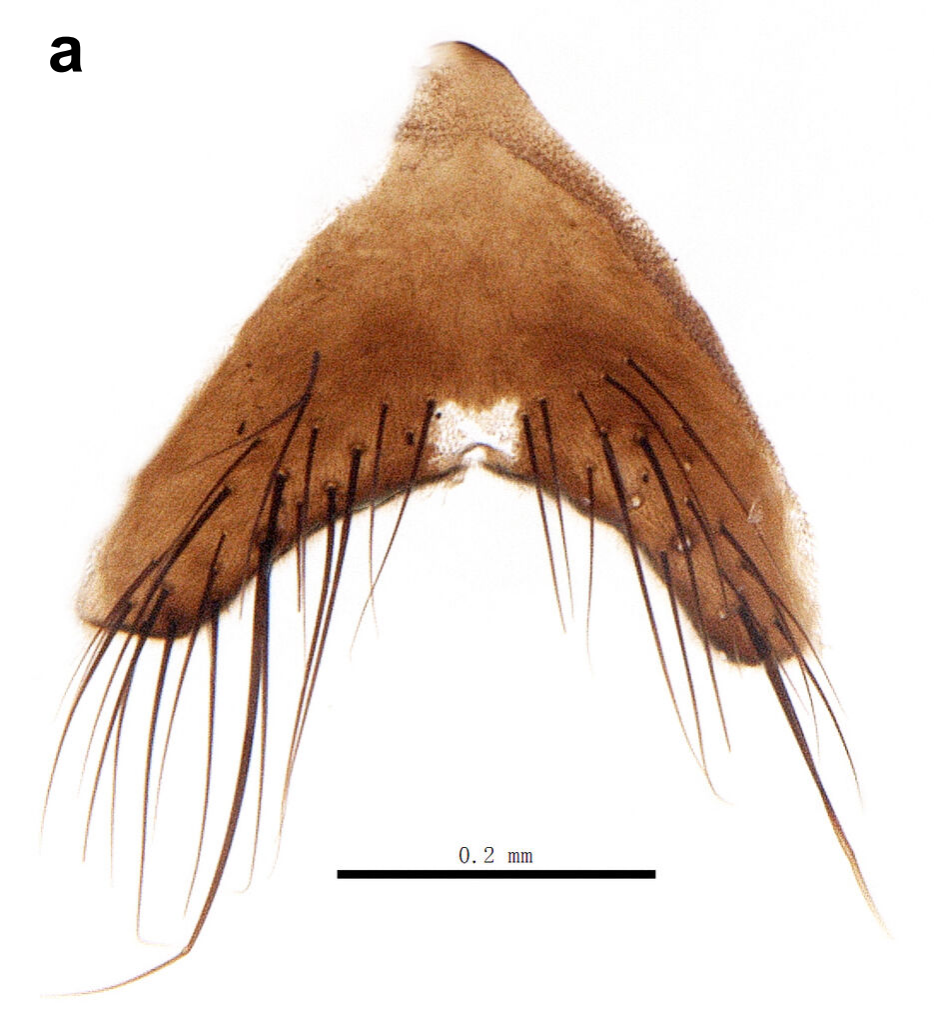
sternite 5 in ventral view.

**Figure 2b.**
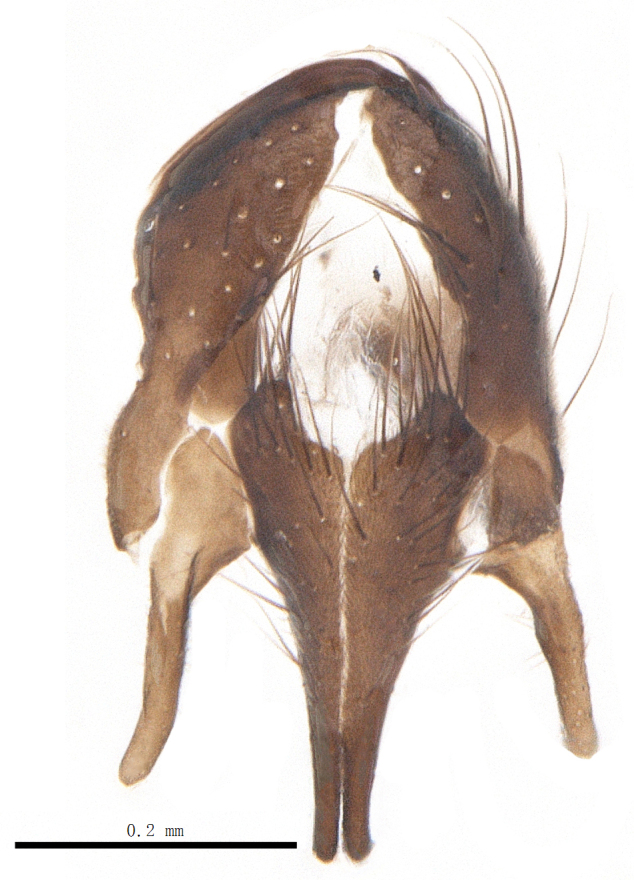
male cerci, surstyli and endium in caudal view.

**Figure 2c.**
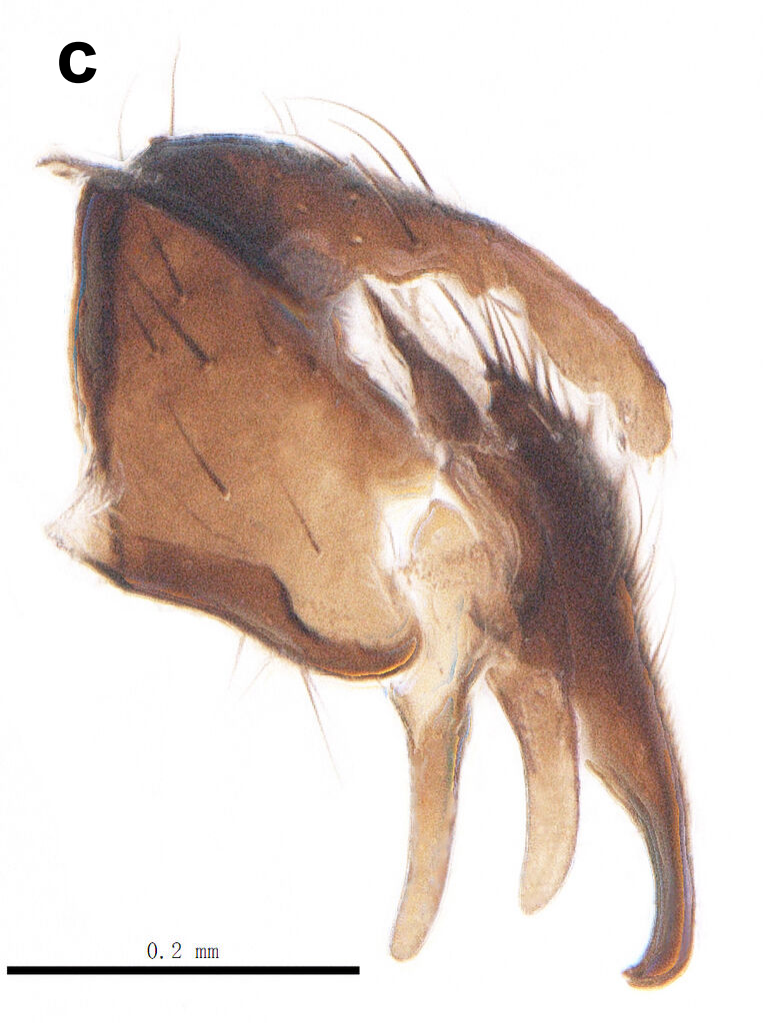
male cerci, surstyli and epandium in lateral view.

**Figure 2d.**
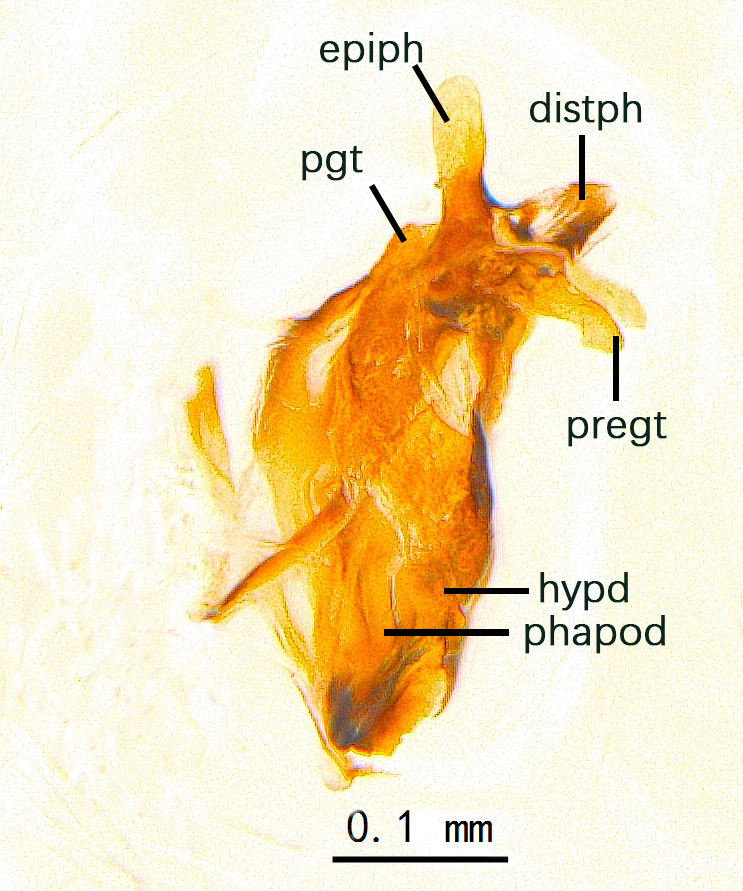
male phallus in lateral view.

**Figure 3a.**
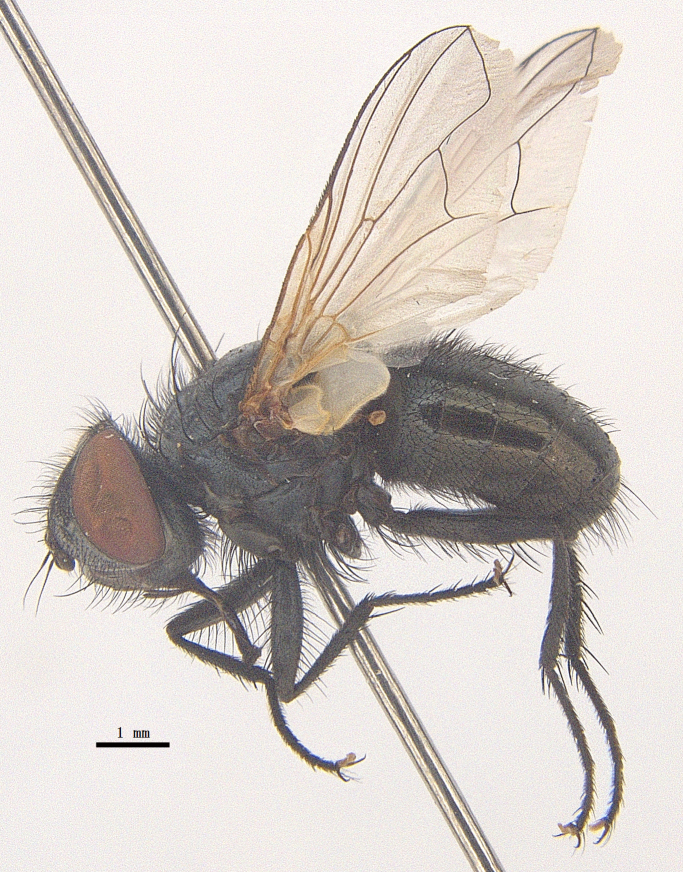
male body in lateral view.

**Figure 3b.**
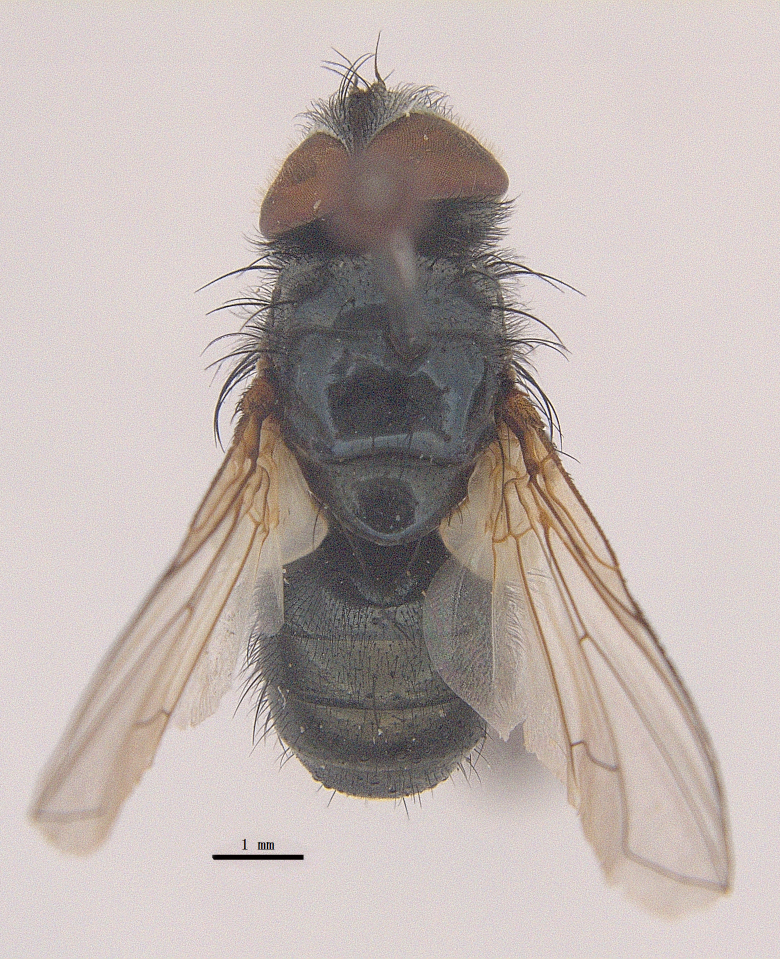
male body in dorsal view.

**Figure 3c.**
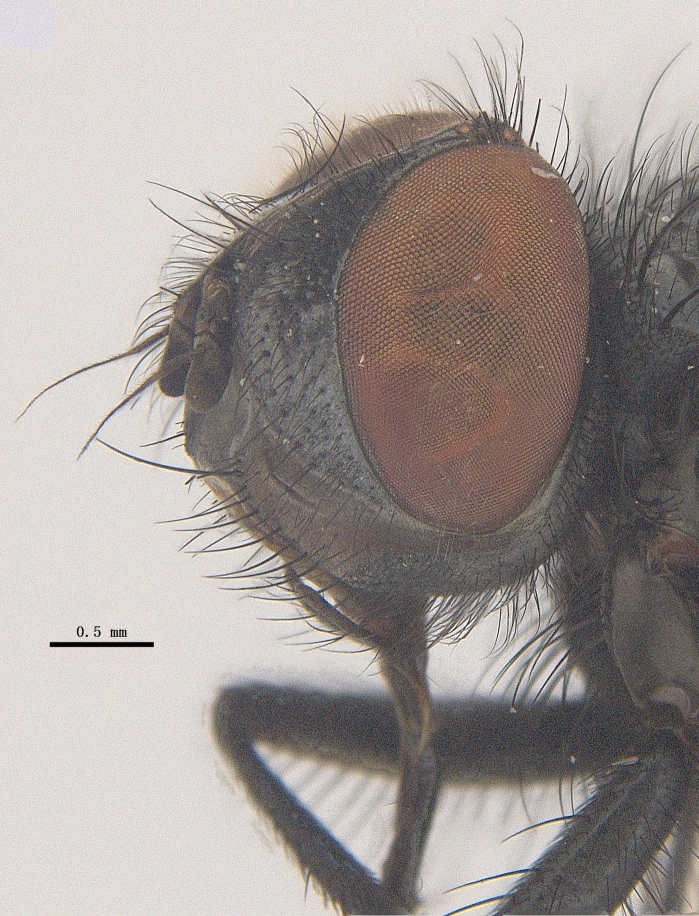
male head in lateral view.

**Figure 3d.**
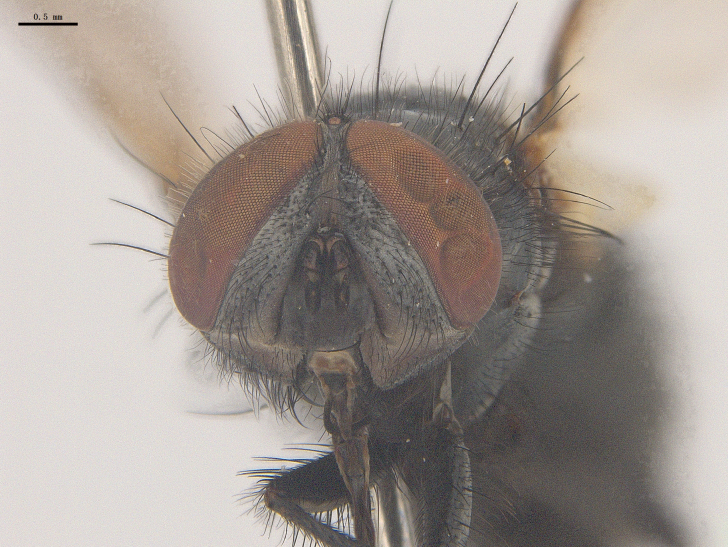
male head in anterior view.

**Figure 4a.**
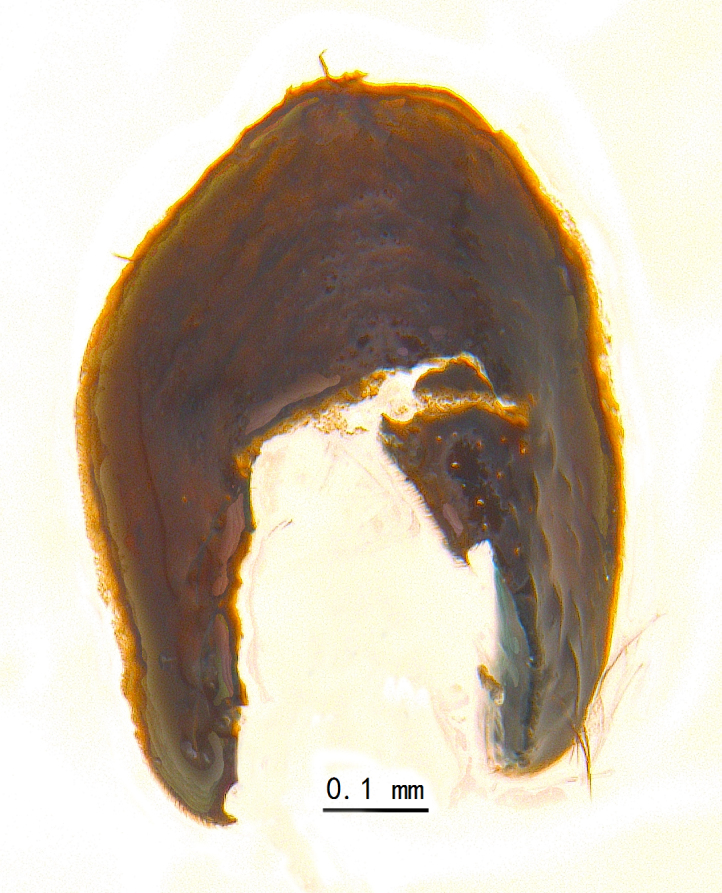
sternite 5 in ventral view.

**Figure 4b.**
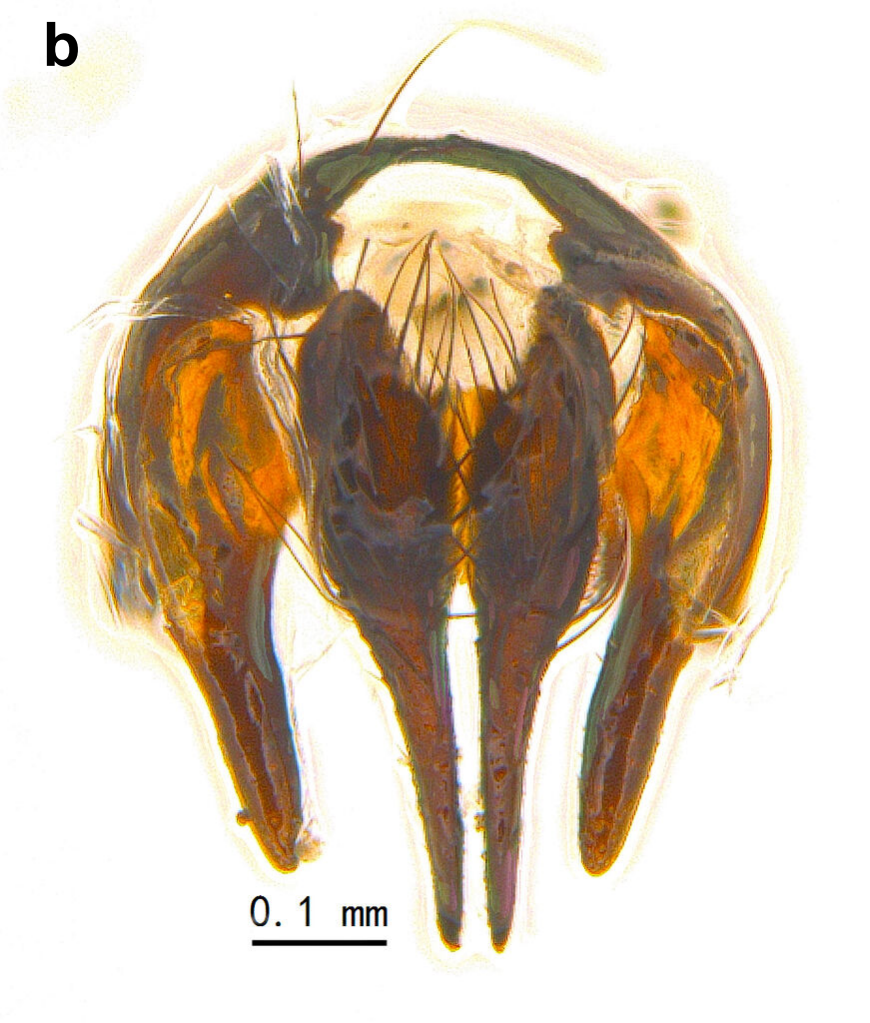
male cerci, surstyli and endium in caudal view.

**Figure 4c.**
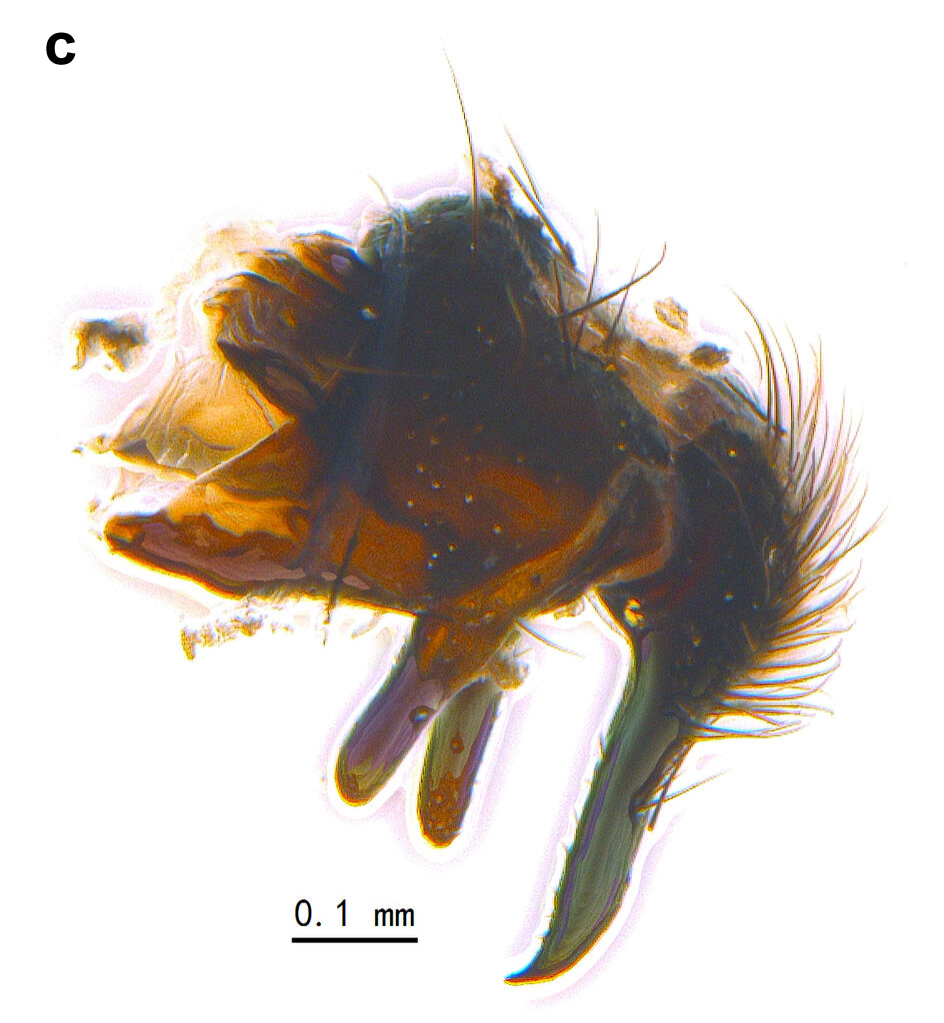
male cerci, surstyli and epandium in lateral view.

**Figure 4d.**
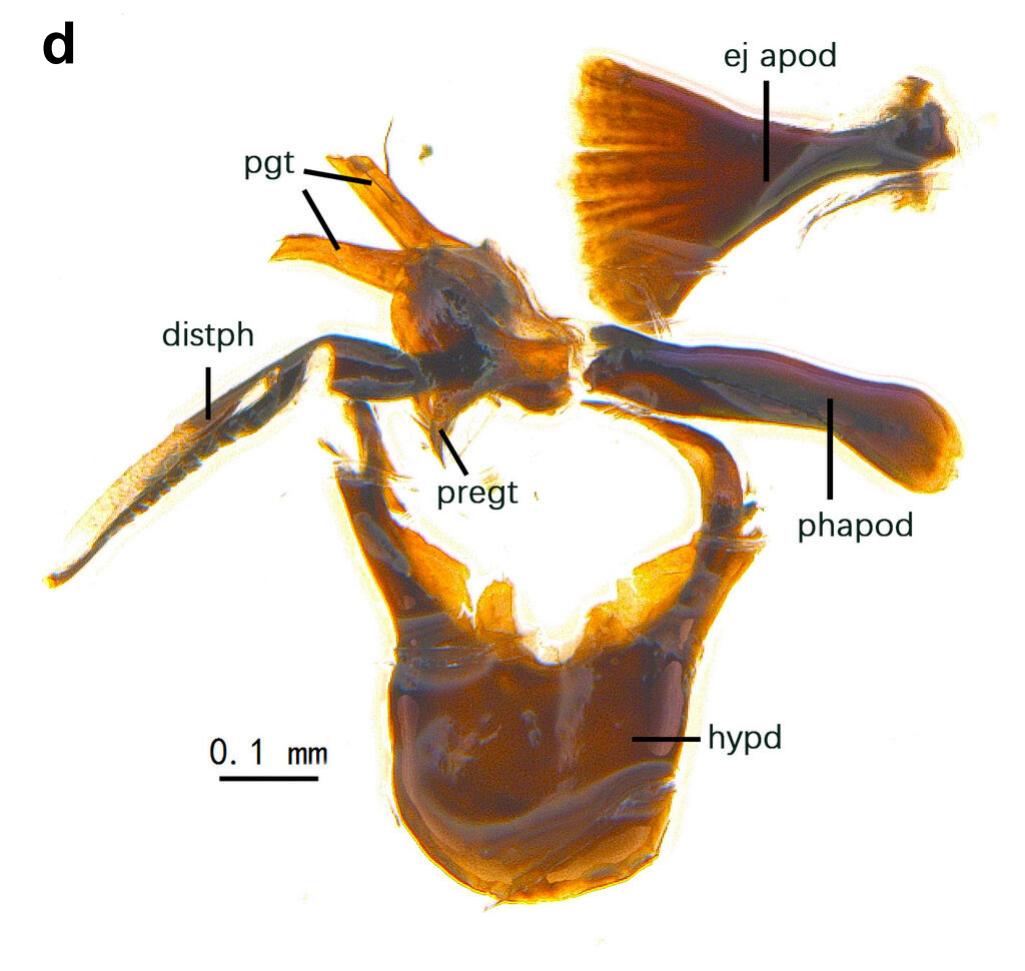
male phallus in lateral view.

**Figure 5a.**
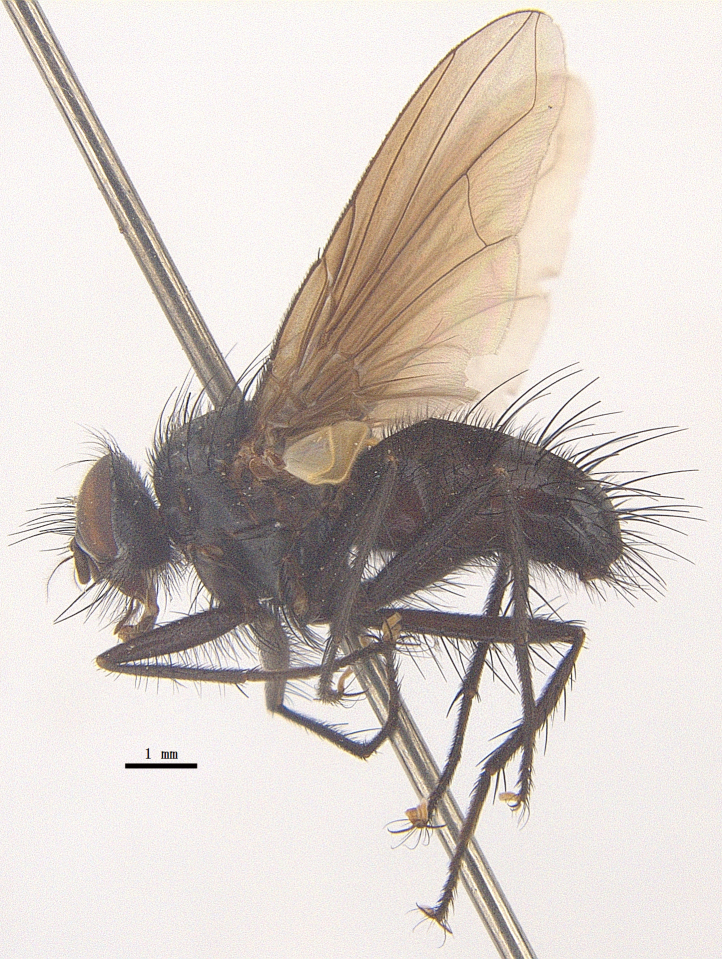
male body in lateral view.

**Figure 5b.**
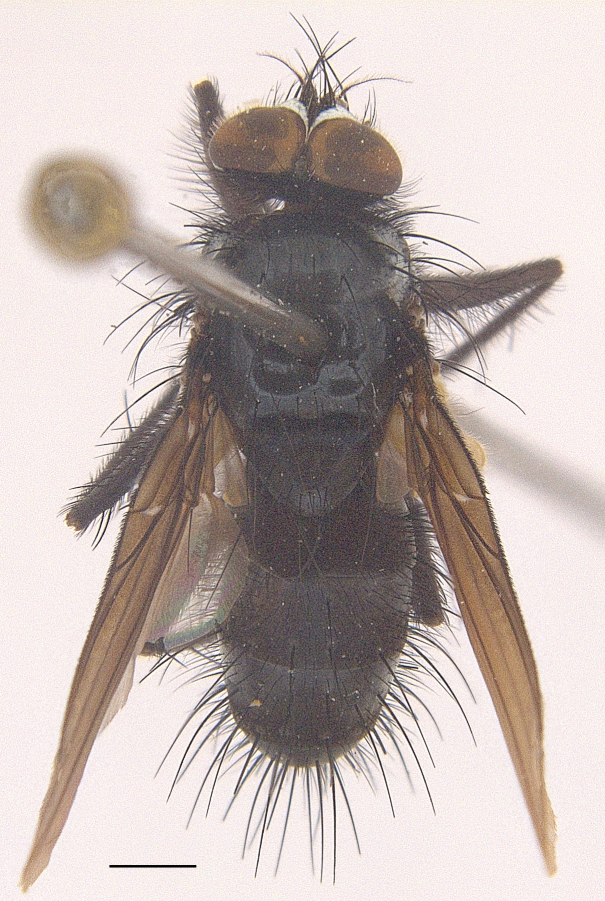
male body in dorsal view.

**Figure 5c.**
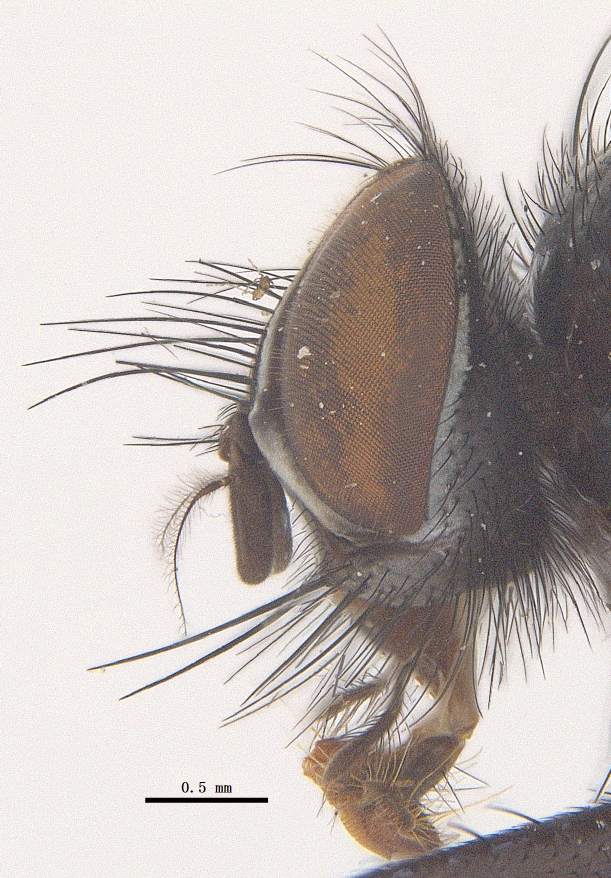
male head in lateral view.

**Figure 5d.**
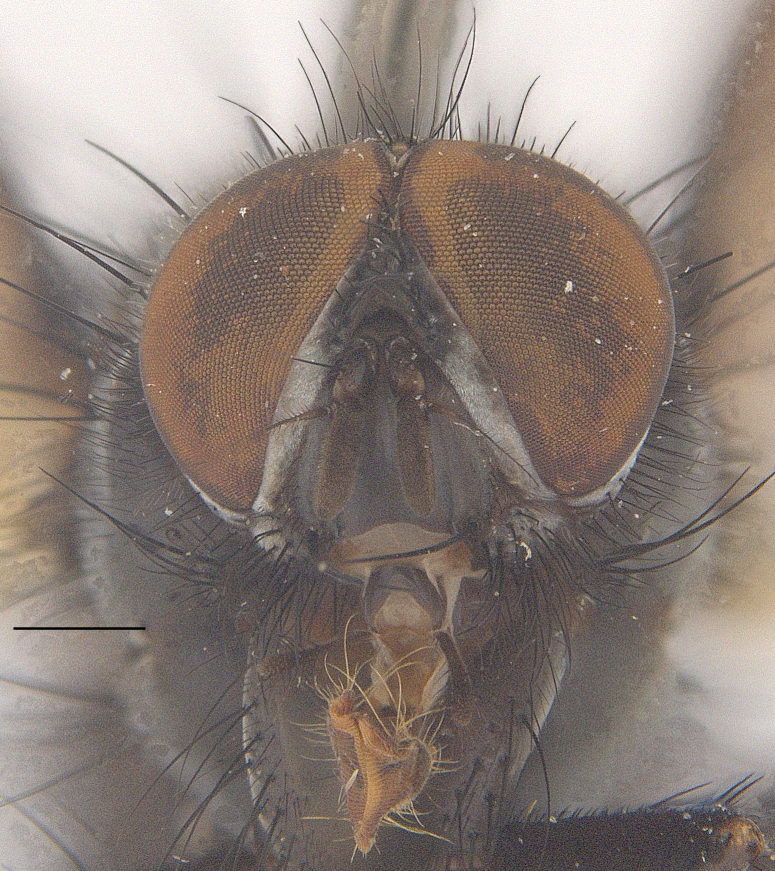
male head in anterior view.

**Figure 6a.**
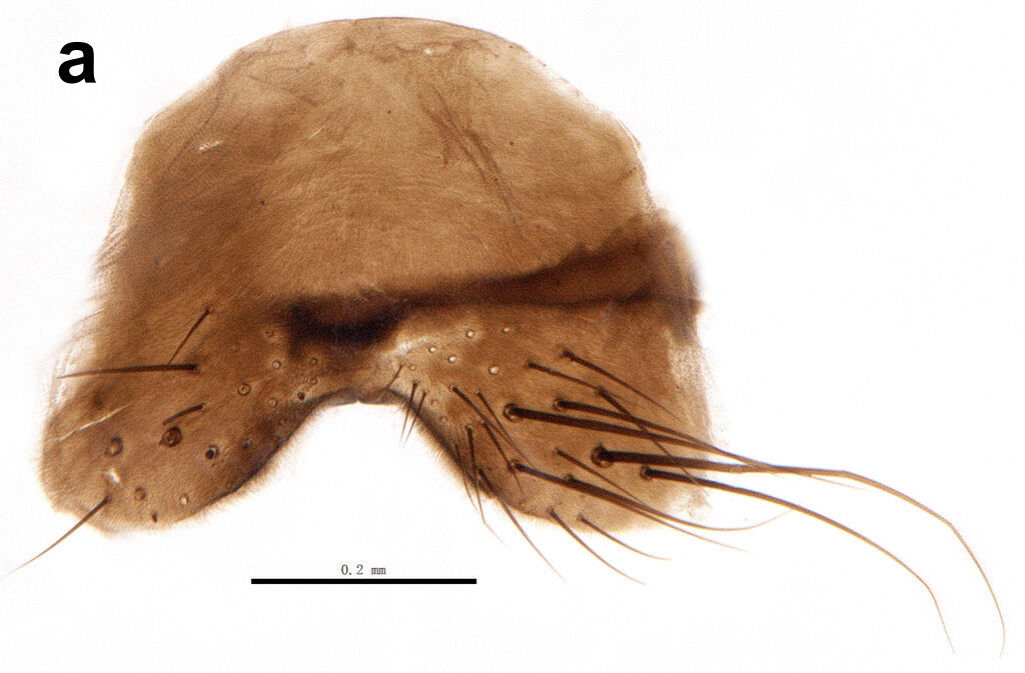
sternite 5 in ventral view.

**Figure 6b.**
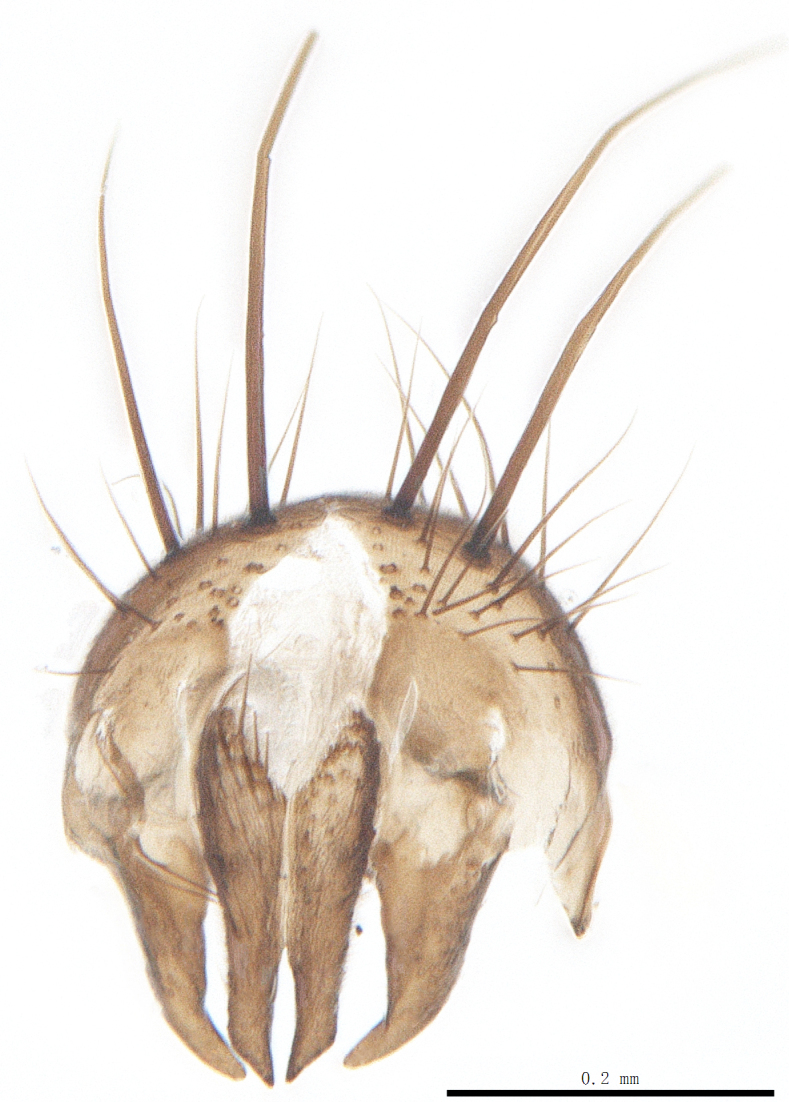
male cerci, surstyli and epandium in caudal view.

**Figure 6c.**
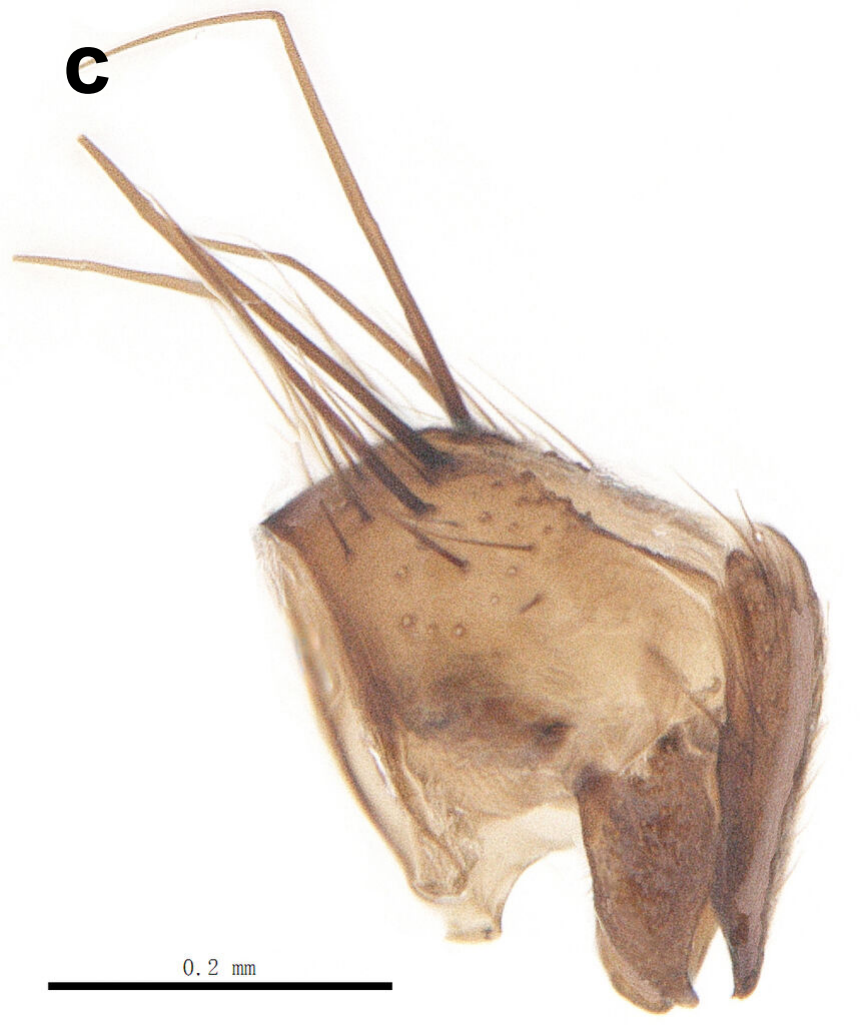
male cerci, surstyli and epandium in lateral view.

**Figure 6d.**
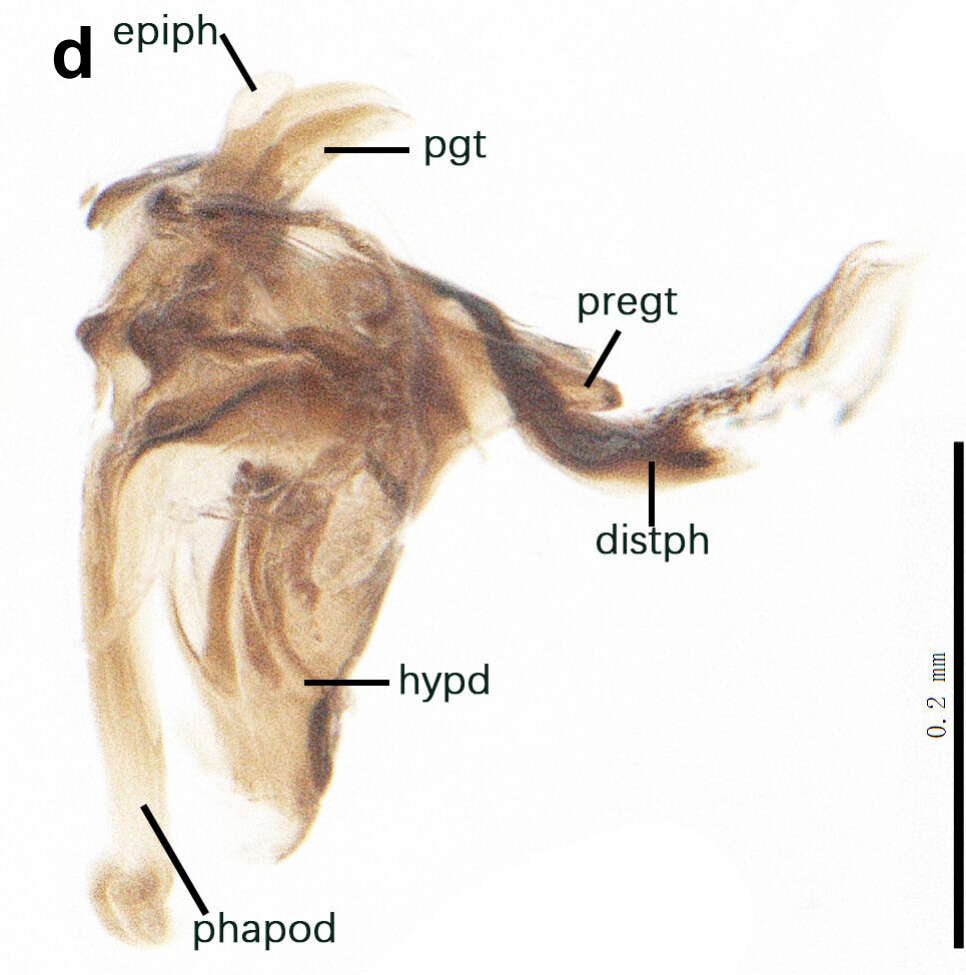
male phallus in lateral view.

**Figure 7a.**
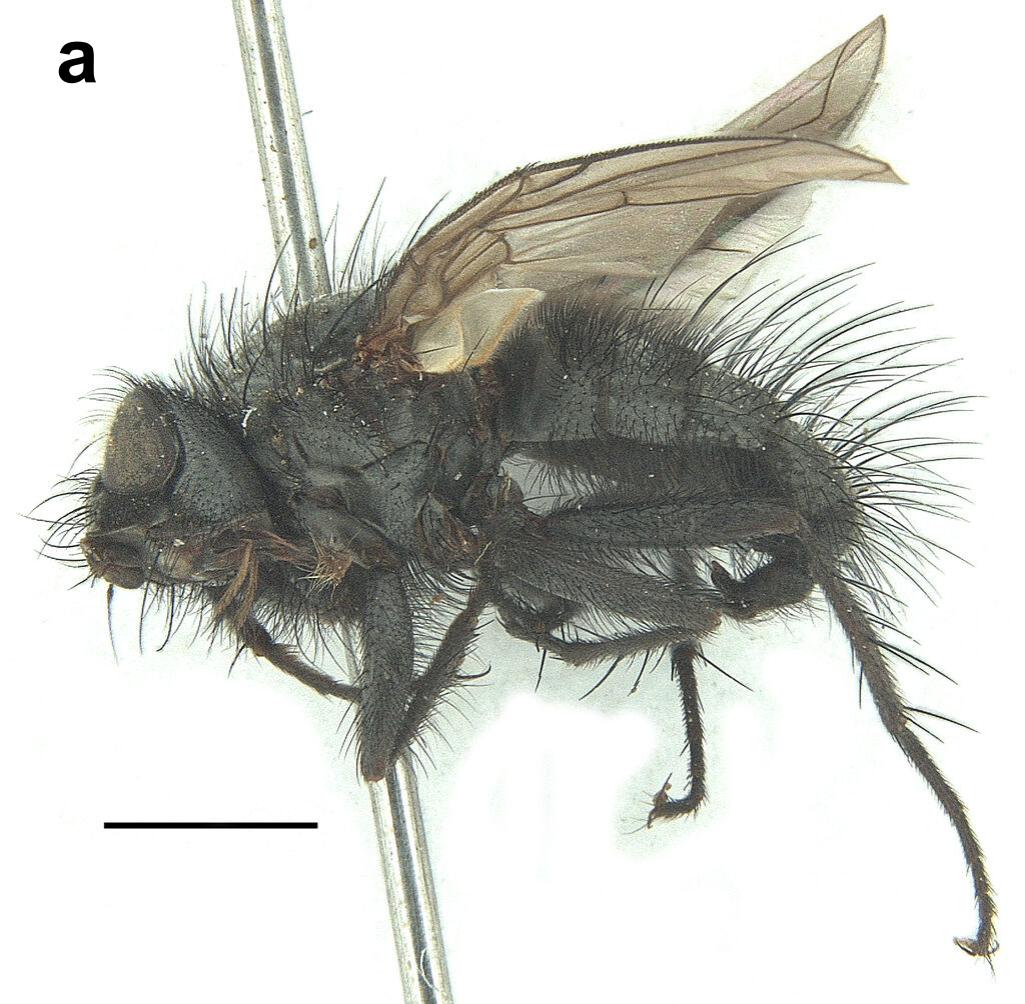
male body in lateral view.

**Figure 7b.**
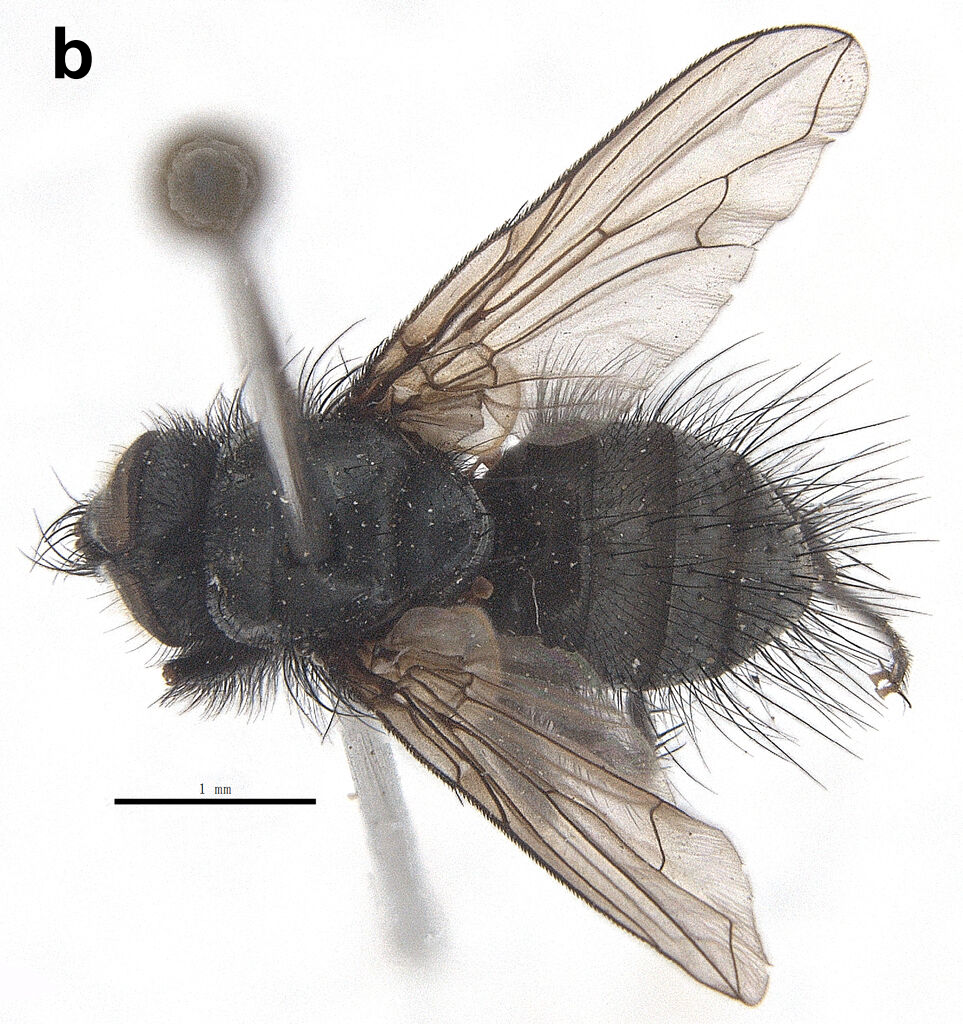
male body in dorsal view.

**Figure 7c.**
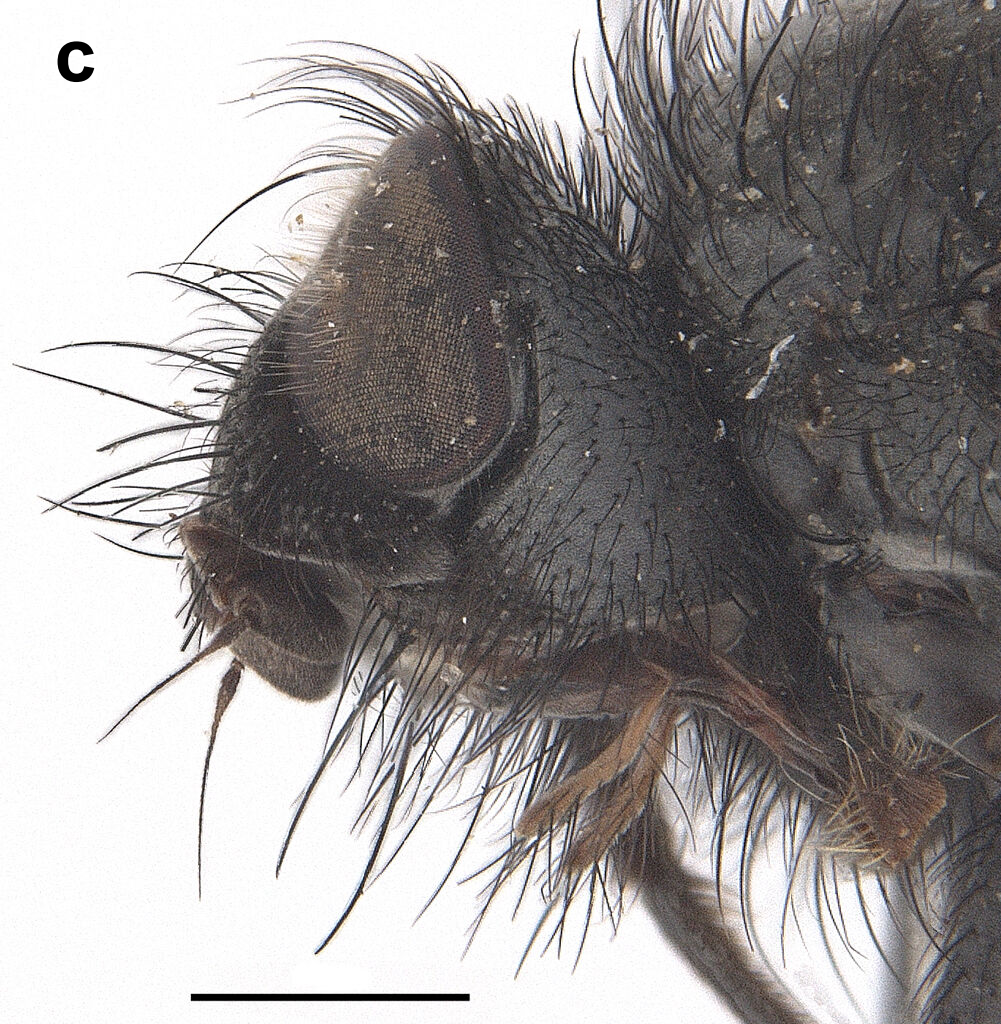
male head in lateral view.

**Figure 7d.**
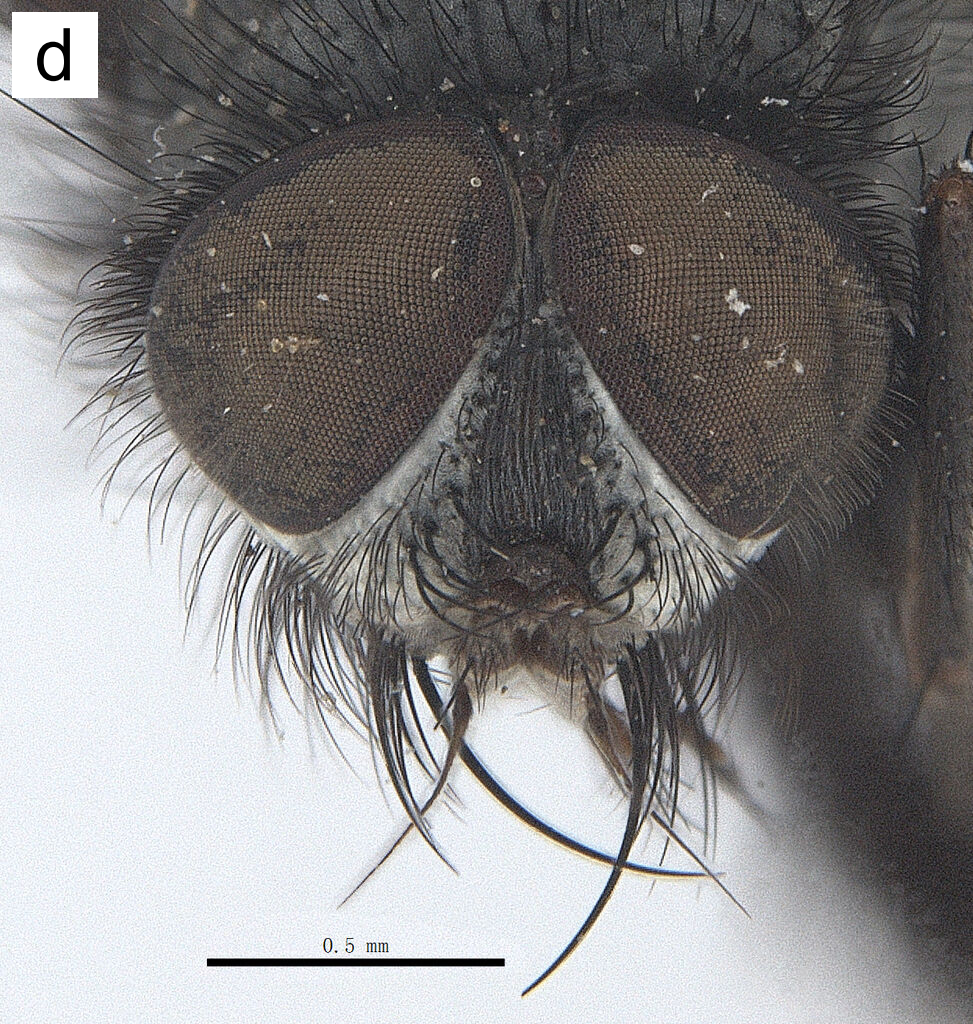
male head in anterior view.

**Figure 8a.**
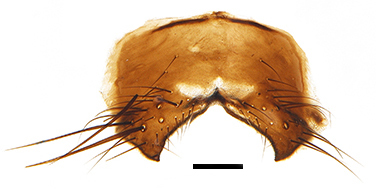
sternite 5 in ventral view.

**Figure 8b.**
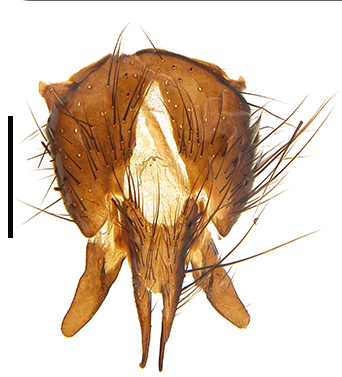
male cerci, surstyli and epandium in caudal view.

**Figure 8c.**
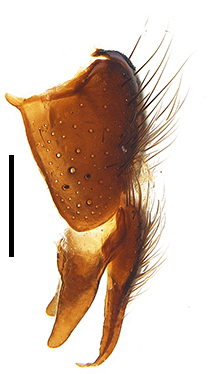
male cerci, surstyli and epandium in lateral view.

**Figure 8d.**
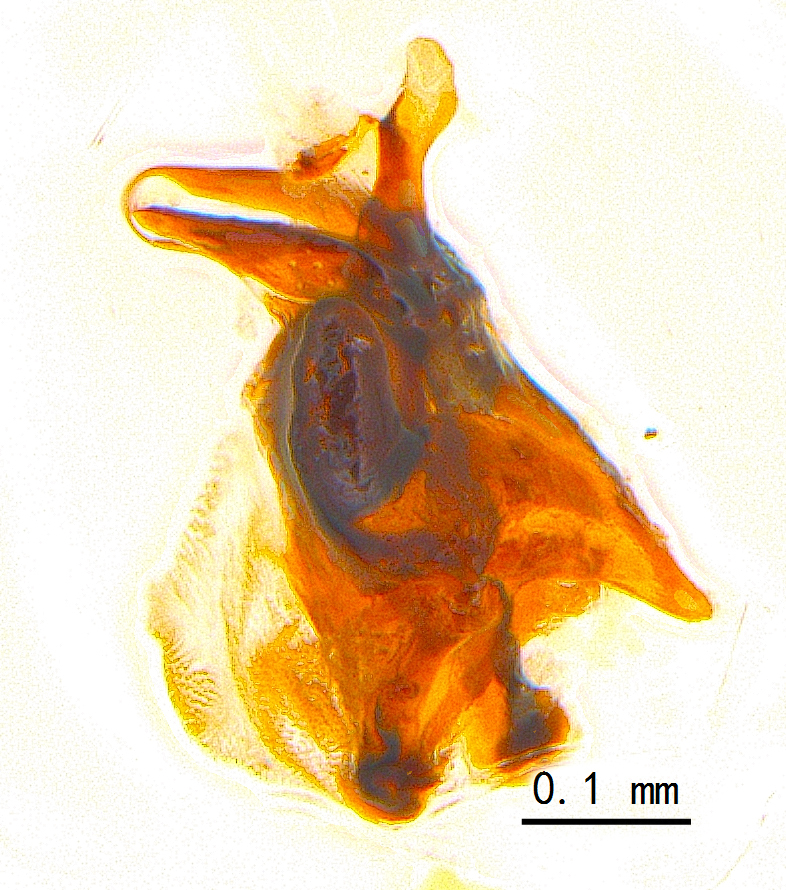
male phallus in lateral view.

**Figure 9a.**
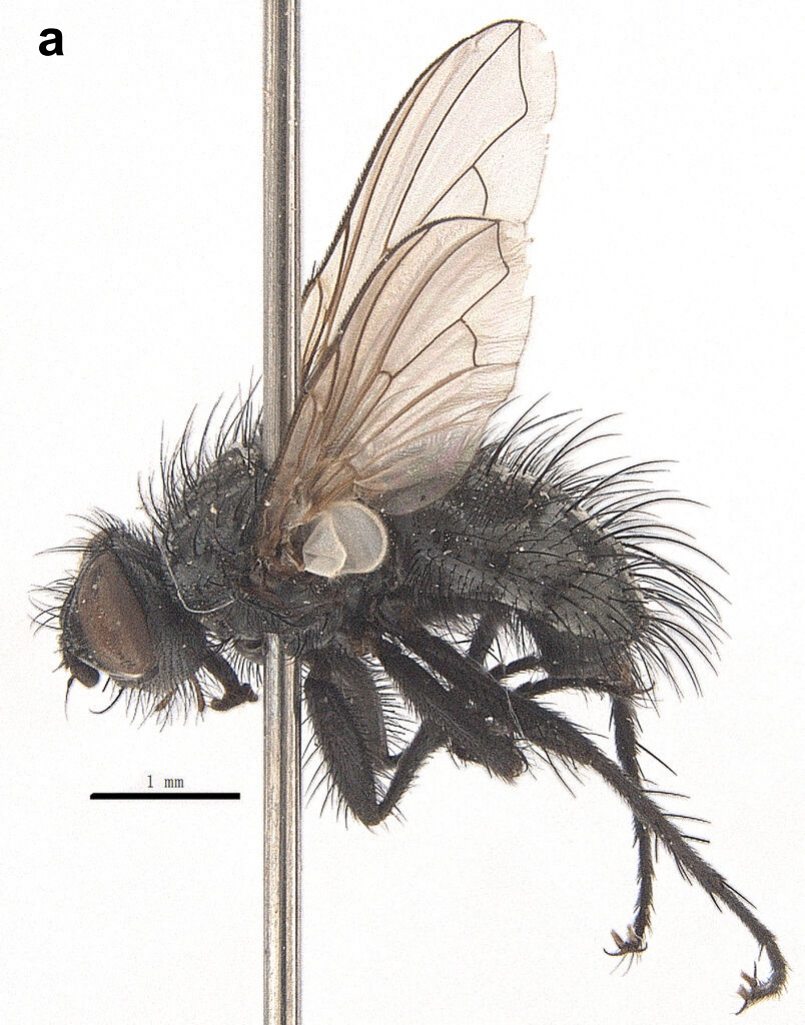
male body in lateral view.

**Figure 9b.**
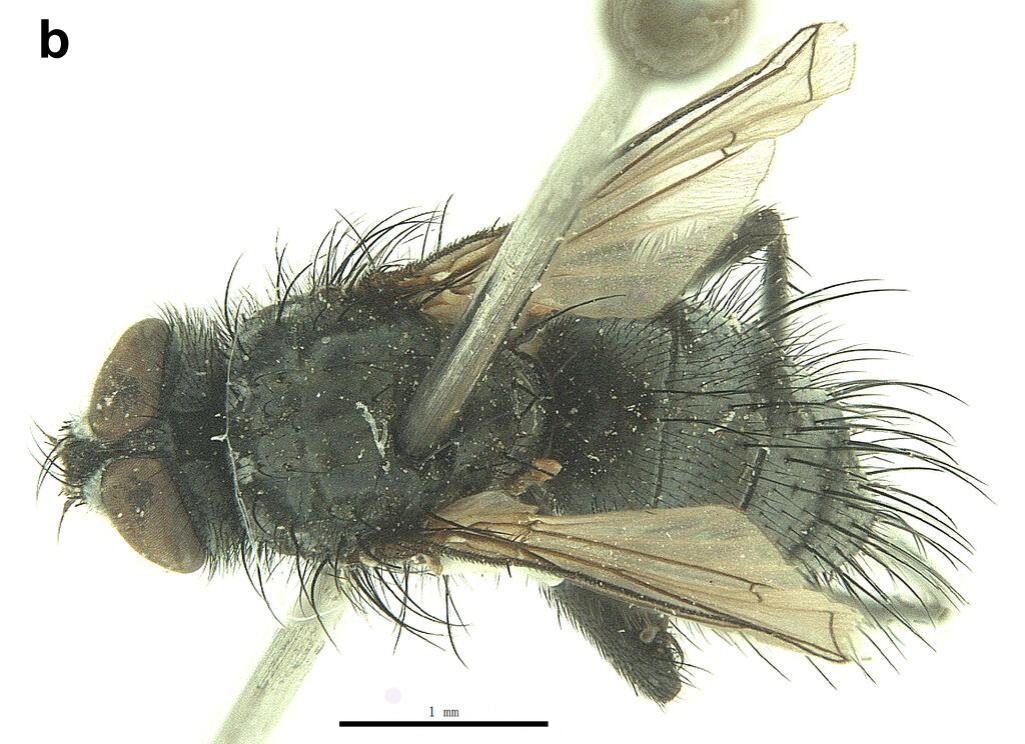
male body in dorsal view.

**Figure 9c.**
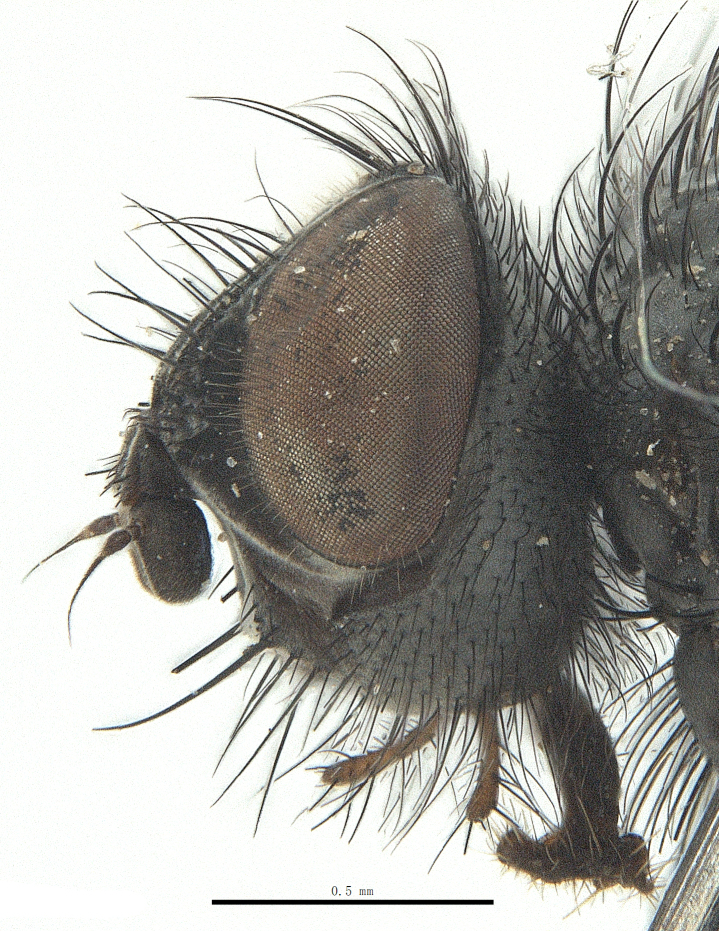
male head in lateral view.

**Figure 9d.**
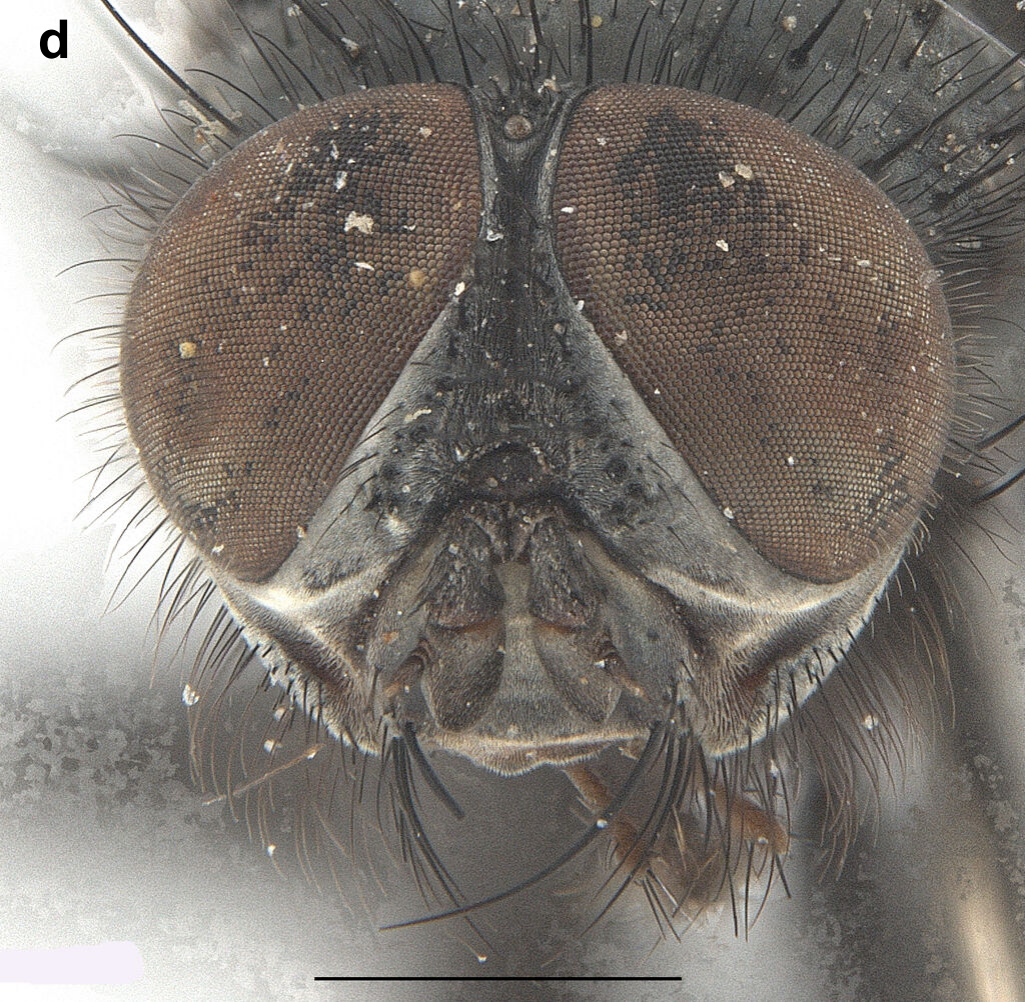
male head in anterior view.

**Figure 10a.**
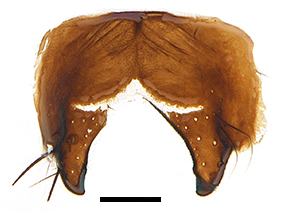
sternite 5 in ventral view.

**Figure 10b.**
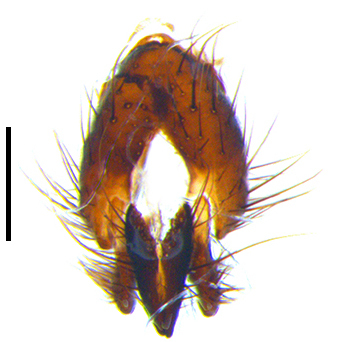
male cerci, surstyli and epandium in caudal view.

**Figure 10c.**
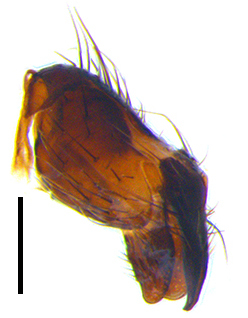
male cerci, surstyli and epandium in lateral view.

**Figure 10d.**
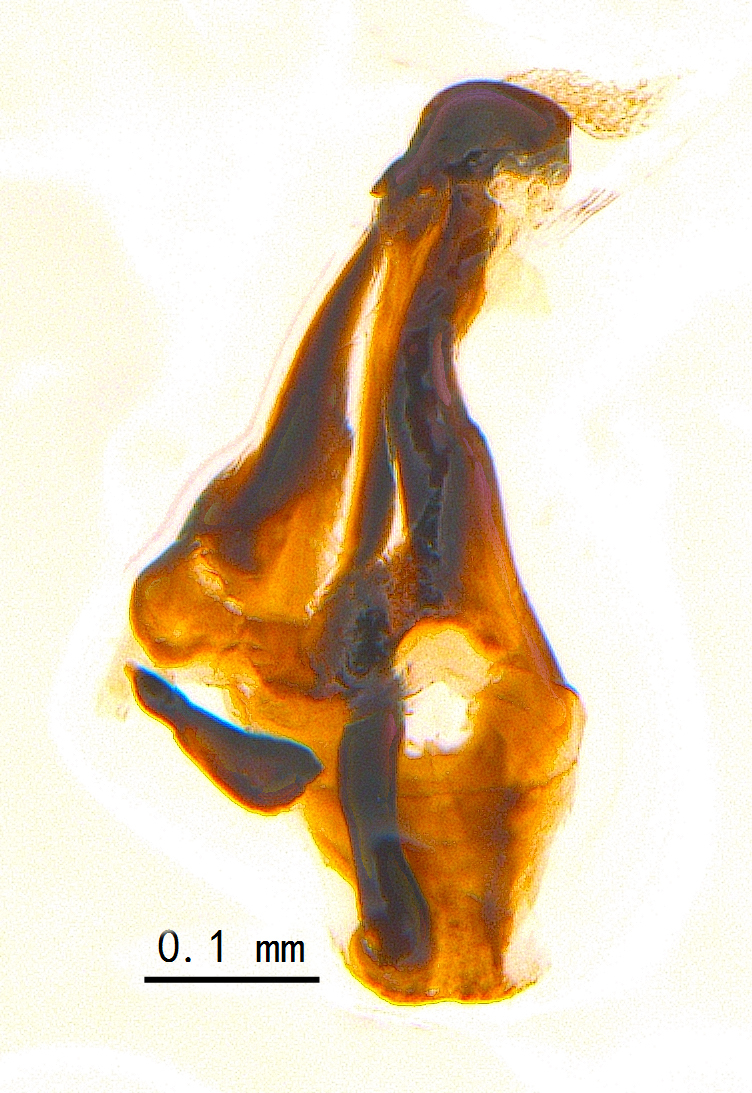
male phallus in lateral view.

**Figure 11a.**
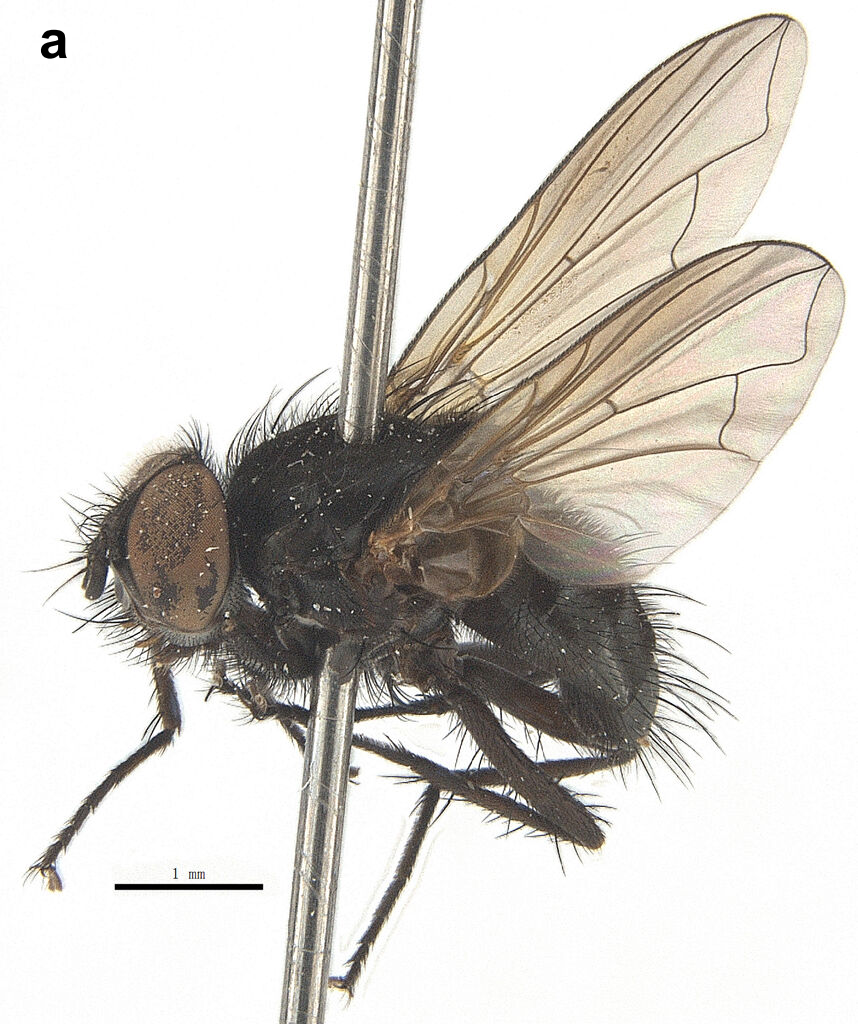
male body in lateral view.

**Figure 11b.**
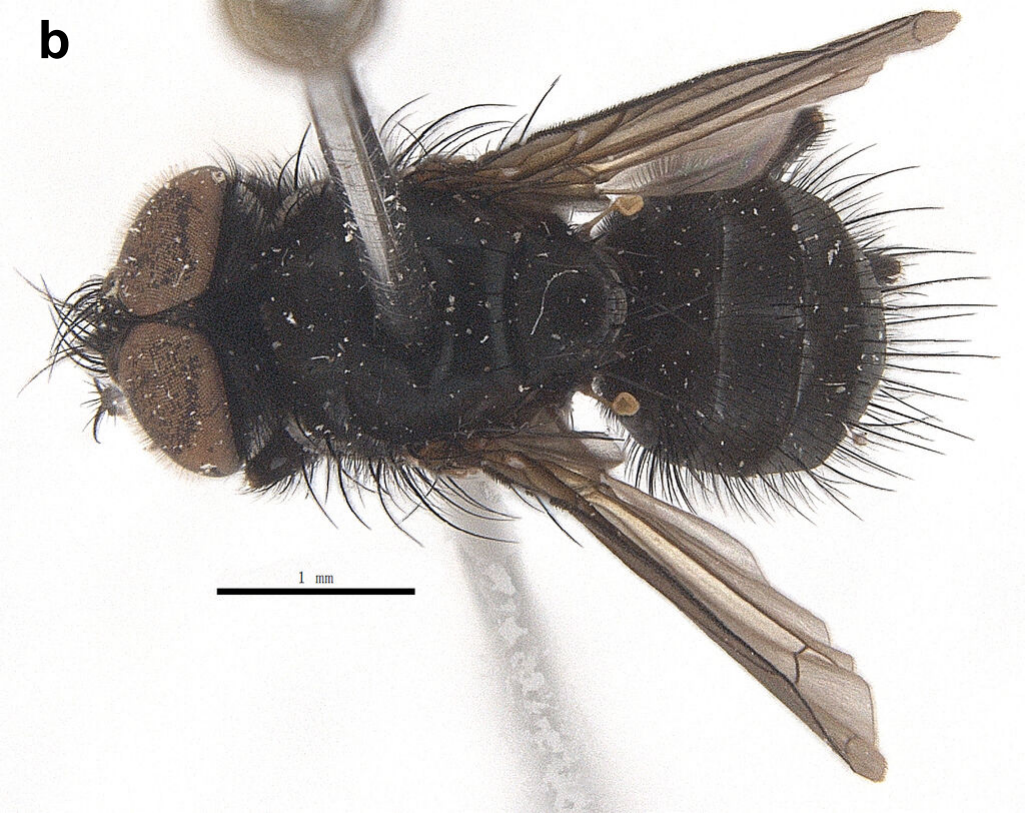
male body in dorsal view.

**Figure 11c.**
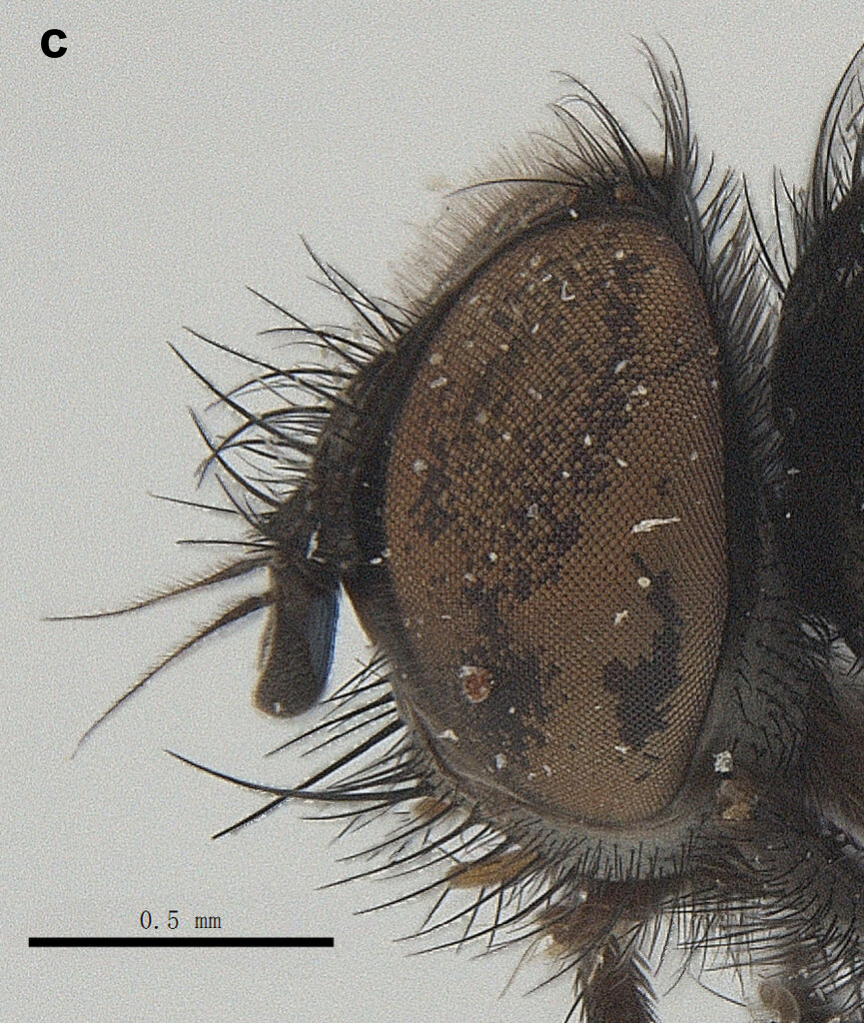
male head in lateral view.

**Figure 11d.**
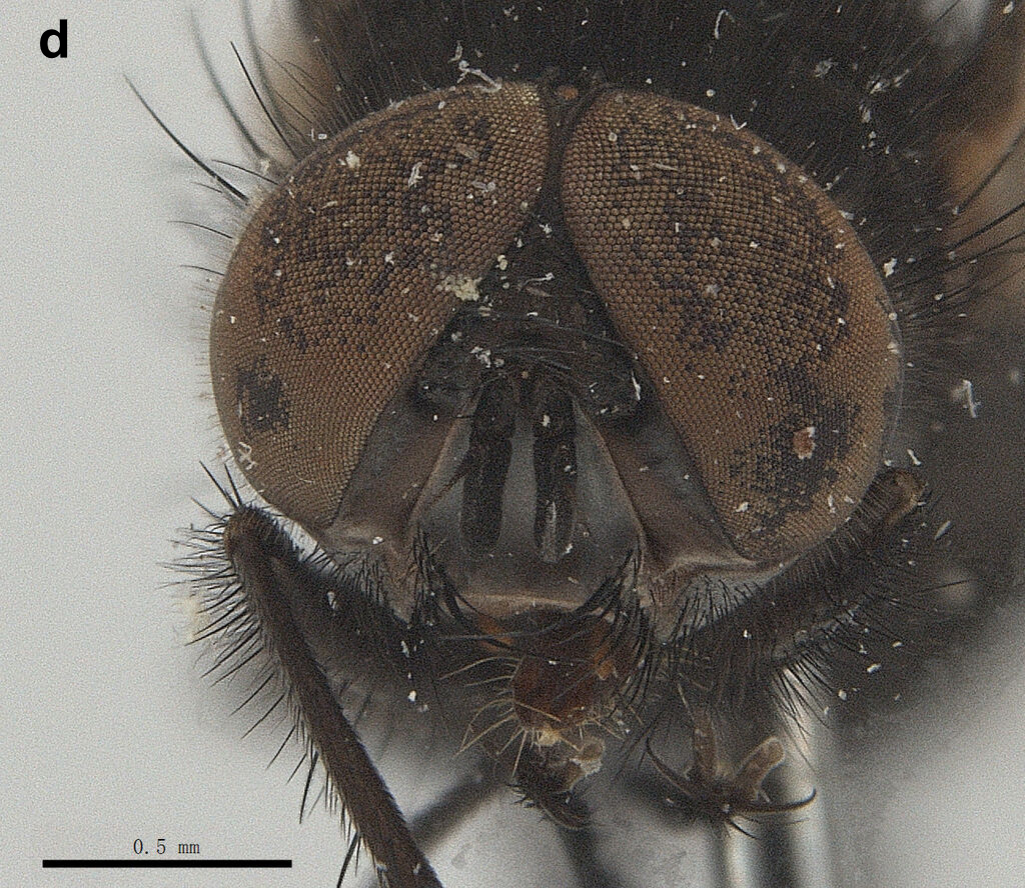
male head in anterior view.

**Figure 12a.**
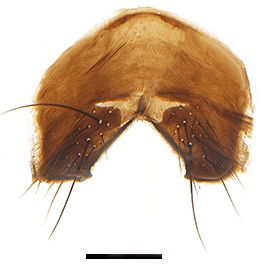
sternite 5 in ventral view.

**Figure 12b.**
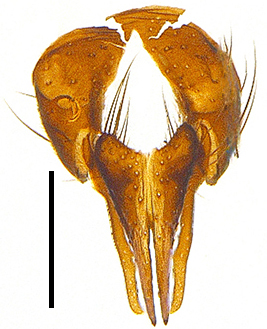
male cerci, surstyli and epandium in caudal view.

**Figure 12c.**
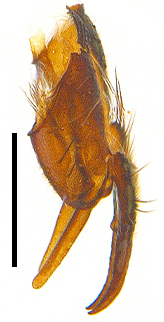
male cerci, surstyli and epandium in lateral view.

**Figure 12d.**
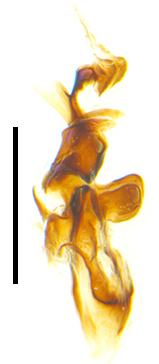
male phallus in lateral view.

**Figure 13a.**
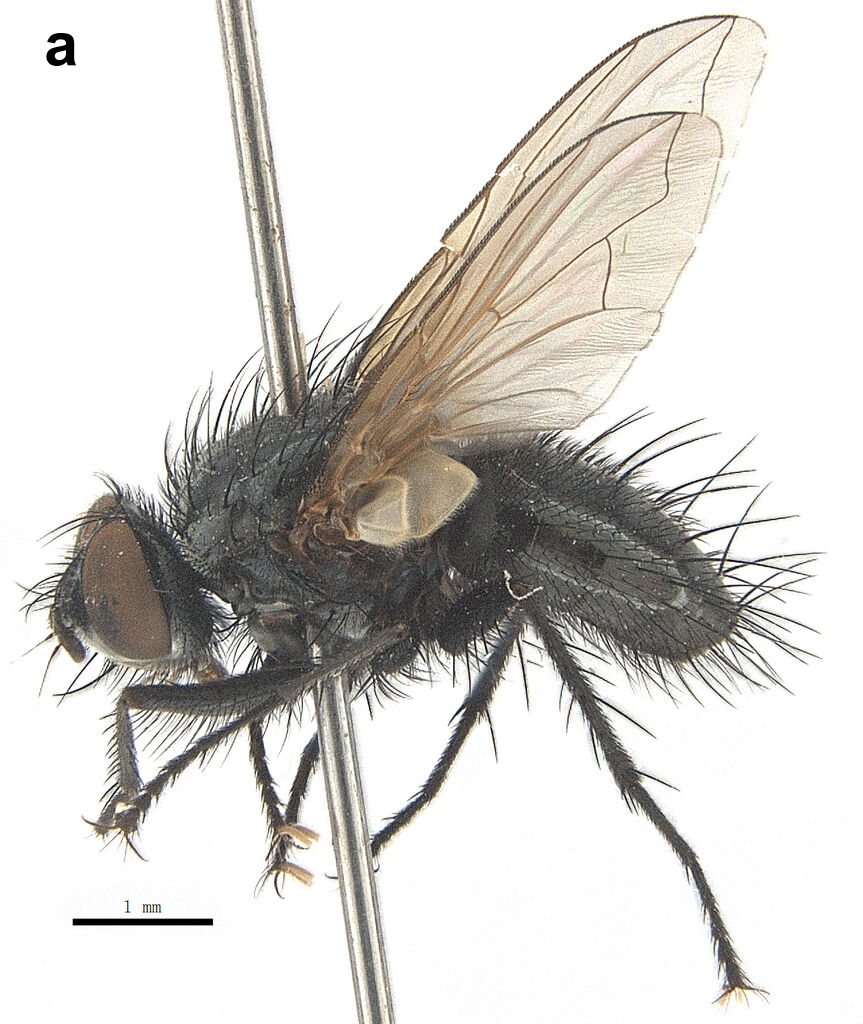
male body in lateral view.

**Figure 13b.**
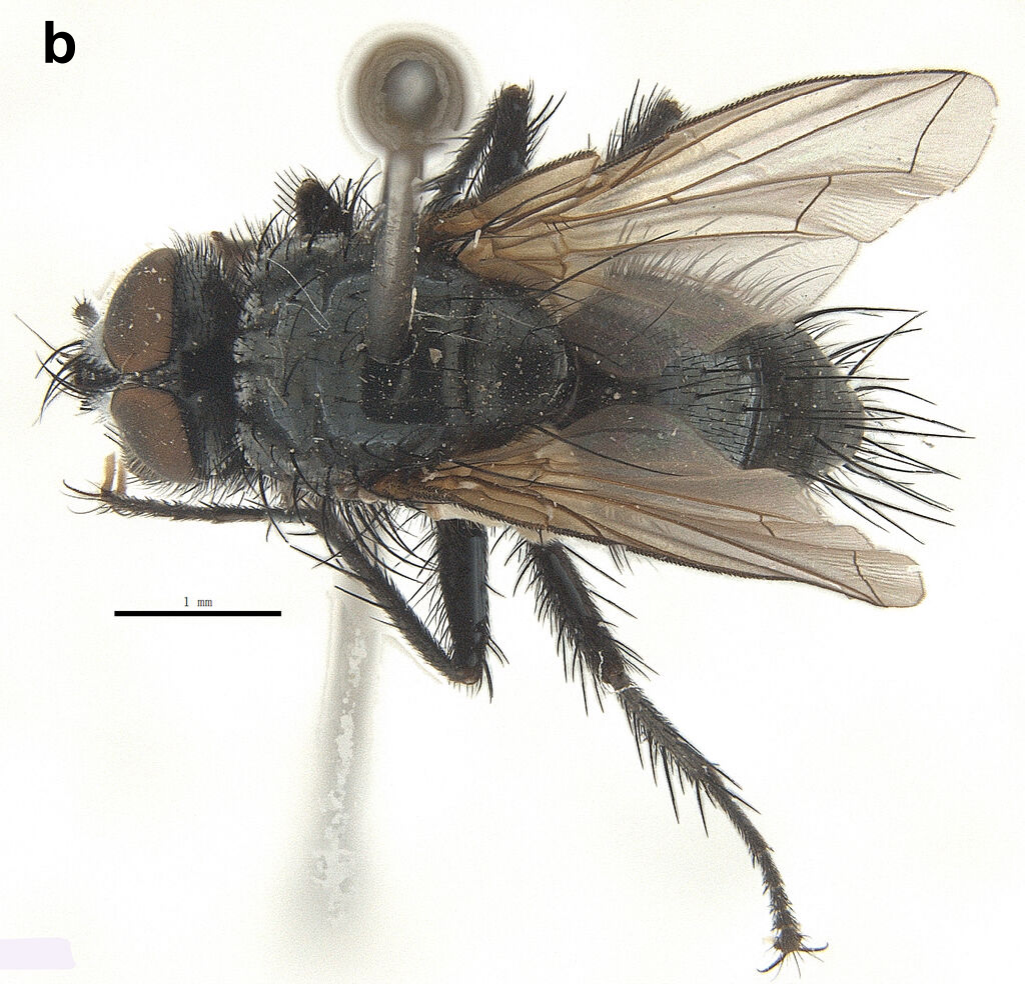
male body in dorsal view.

**Figure 13c.**
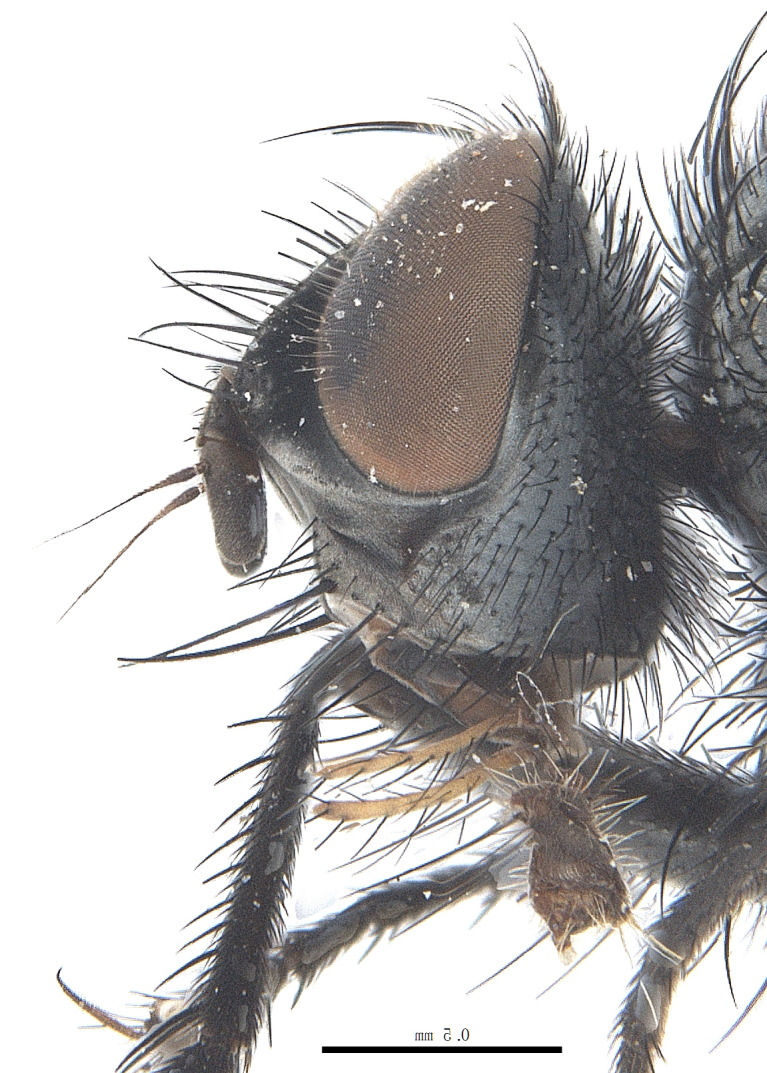
male head in lateral view.

**Figure 13d.**
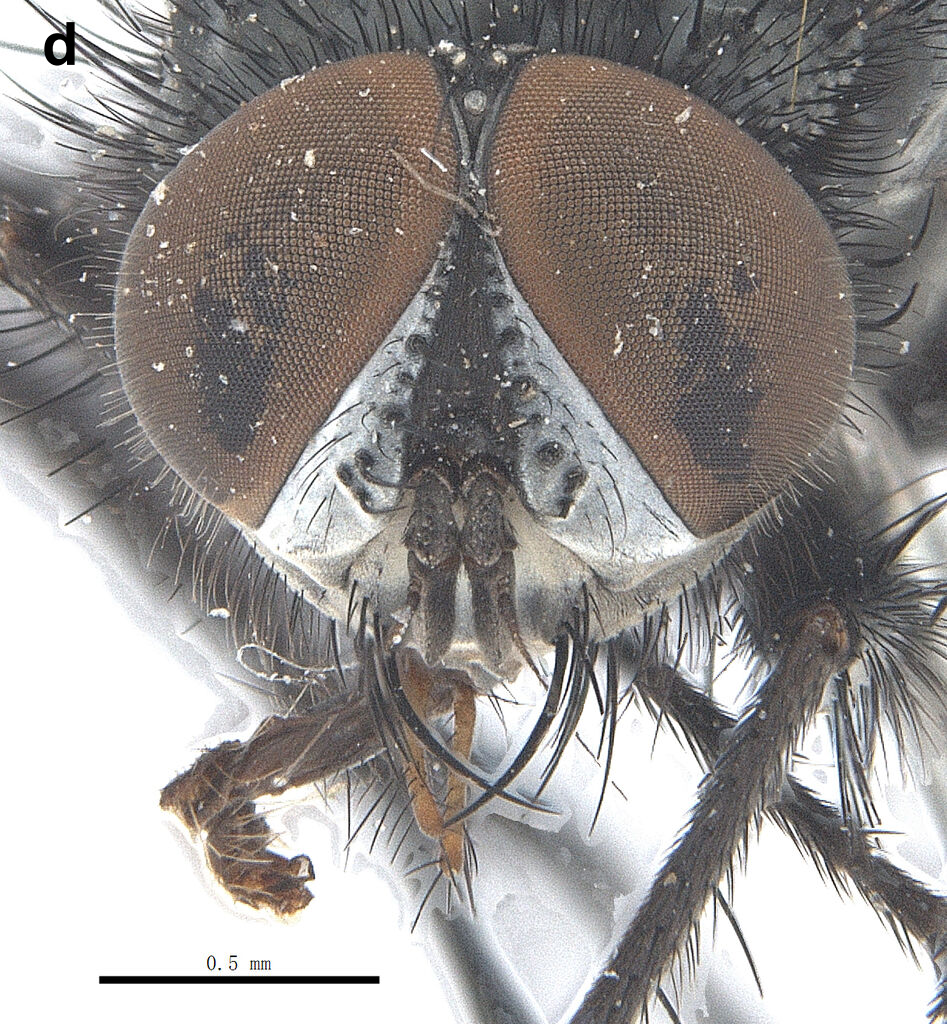
male head in anterior view.

**Figure 14a.**
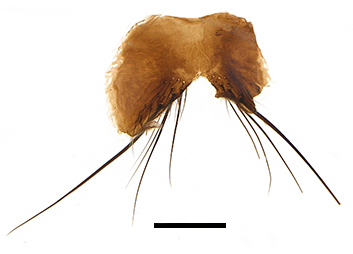
sternite 5 in ventral view.

**Figure 14b.**
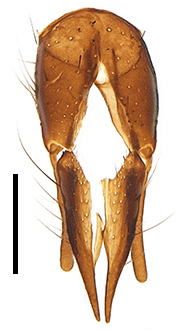
male cerci, surstyli and epandium in caudal view.

**Figure 14c.**
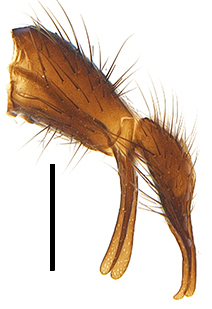
male cerci, surstyli and epandium in lateral view.

**Figure 14d.**
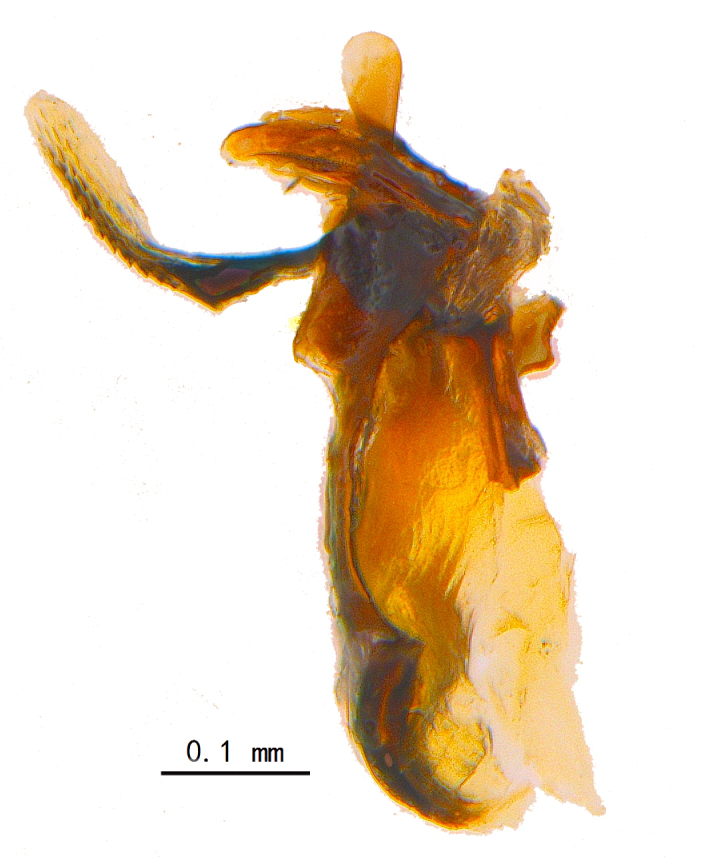
male phallus in lateral view.

**Figure 15a.**
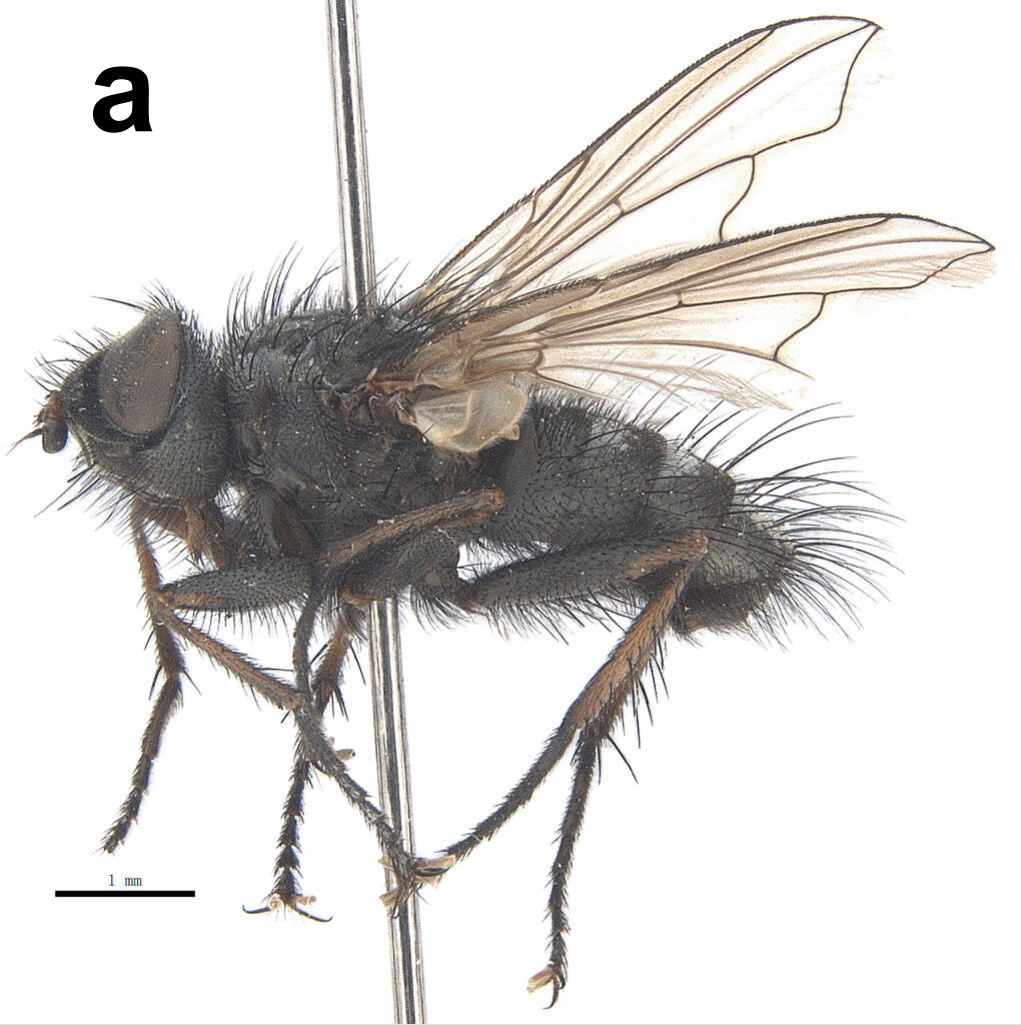
male body in lateral view.

**Figure 15b.**
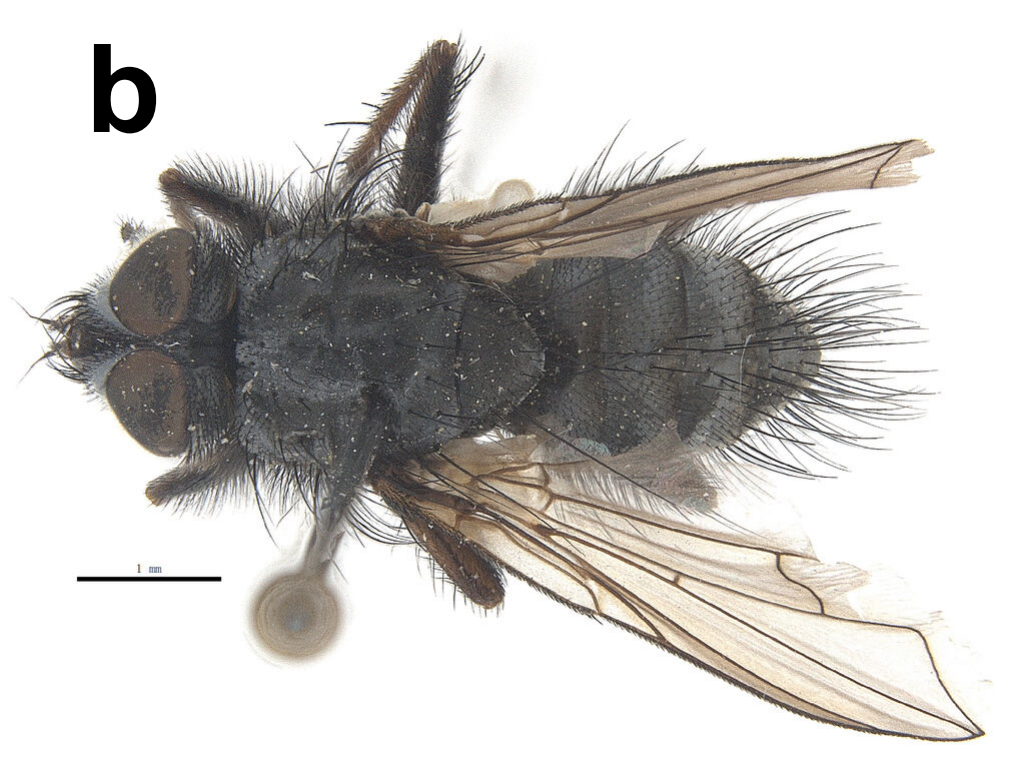
male body in dorsal view.

**Figure 15c.**
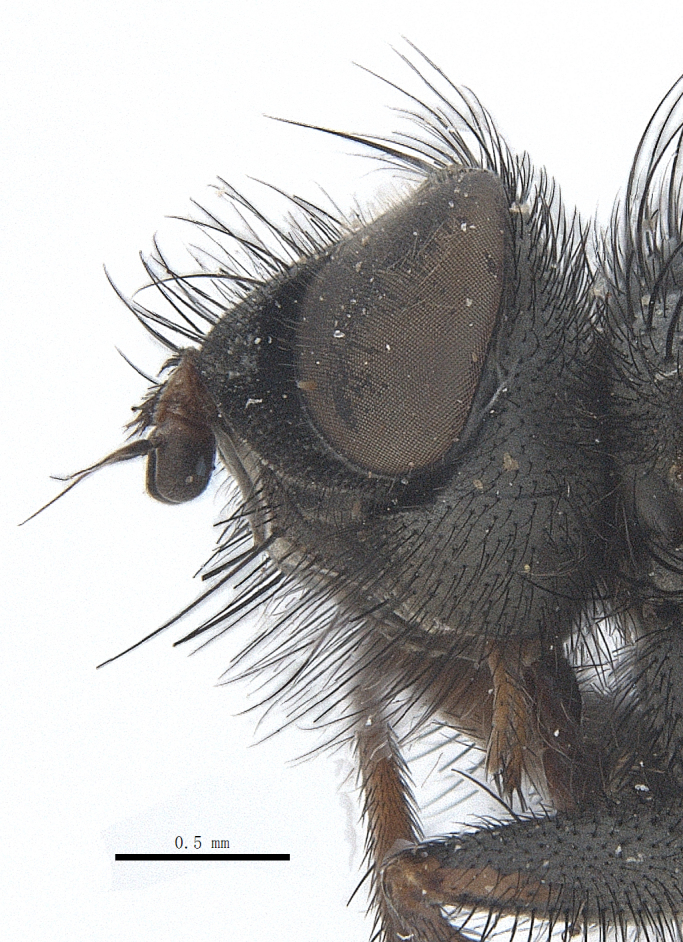
male head in lateral view.

**Figure 15d.**
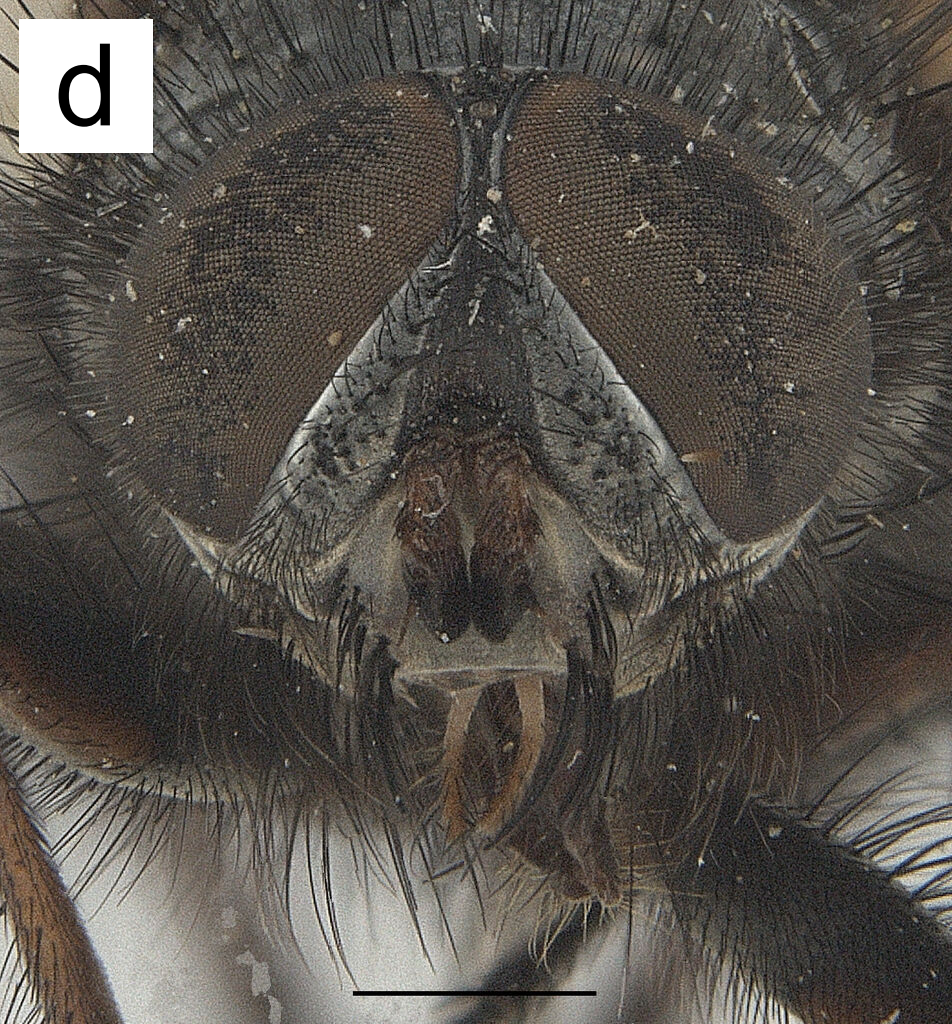
male head in anterior view.

**Figure 16a.**
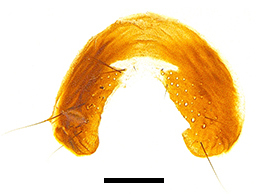
sternite 5 in ventral view.

**Figure 16b.**
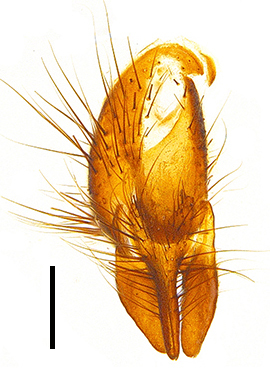
male cerci, surstyli and epandium in caudal view.

**Figure 16c.**
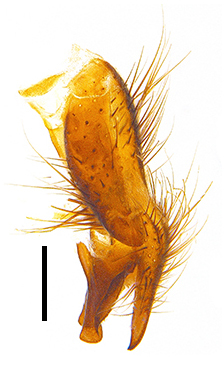
male cerci, surstyli and epandium in lateral view.

**Figure 16d.**
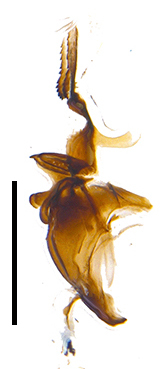
male phallus in lateral view.

**Table 1.** Comparison of three new species *of Macquartia* Robineau-Desvoidy.

***M.barkamensis* sp. n.**	***M.setifacies* sp. n.**	***M.sichuanensis* sp. n.**
Parafacial hairy at most on upper half	Parafacial hairy on whole length	Parafacial bare
Arista short hair-like	Arista short pubescent	Arista plumose
Basicosta reddish-yellow	Basicosta reddish-yellow	Basicosta black
Mid-tibia with one anterodorsal seta	Mid-tibia with three anterodorsal setae	Mid-tibia with one anterodorsal seta
Abdominal syntergite 1+2 with two median marginal and two lateral marginal setae	Abdominal syntergite 1+2 with two median marginal and two lateral marginal setae	Abdominal syntergite 1+2 with two lateral marginal setae, median marginal seta absent.
